# Comparative analysis estimates the relative frequencies of co-divergence and cross-species transmission within viral families

**DOI:** 10.1371/journal.ppat.1006215

**Published:** 2017-02-08

**Authors:** Jemma L. Geoghegan, Sebastián Duchêne, Edward C. Holmes

**Affiliations:** 1 Marie Bashir Institute for Infectious Diseases and Biosecurity, Charles Perkins Centre, School of Life and Environmental Sciences and Sydney Medical School, The University of Sydney, Sydney, New South Wales, Australia; 2 Centre for Systems Genomics, The University of Melbourne, Melbourne, Victoria, Australia; University of Bonn, GERMANY

## Abstract

The cross-species transmission of viruses from one host species to another is responsible for the majority of emerging infections. However, it is unclear whether some virus families have a greater propensity to jump host species than others. If related viruses have an evolutionary history of co-divergence with their hosts there should be evidence of topological similarities between the virus and host phylogenetic trees, whereas host jumping generates incongruent tree topologies. By analyzing co-phylogenetic processes in 19 virus families and their eukaryotic hosts we provide a quantitative and comparative estimate of the relative frequency of virus-host co-divergence versus cross-species transmission among virus families. Notably, our analysis reveals that cross-species transmission is a near universal feature of the viruses analyzed here, with virus-host co-divergence occurring less frequently and always on a subset of viruses. Despite the overall high topological incongruence among virus and host phylogenies, the *Hepadnaviridae*, *Polyomaviridae*, *Poxviridae*, *Papillomaviridae* and *Adenoviridae*, all of which possess double-stranded DNA genomes, exhibited more frequent co-divergence than the other virus families studied here. At the other extreme, the virus and host trees for all the RNA viruses studied here, particularly the *Rhabdoviridae* and the *Picornaviridae*, displayed high levels of topological incongruence, indicative of frequent host switching. Overall, we show that cross-species transmission plays a major role in virus evolution, with all the virus families studied here having the potential to jump host species, and that increased sampling will likely reveal more instances of host jumping.

## Author summary

Emerging infectious diseases are often characterized by host switching events, in which a pathogen jumps from its original host to infect a novel species. However, given the ecological and genetic barriers a virus must overcome to jump species and adapt to new hosts, it might be reasonable to assume that successful cross-species transmission is a relatively rare occurrence and that viruses are instead more likely to co-diverge with their hosts. Using a comparative co-phylogenetic analysis performed at the scale of virus family we have revealed that co-divergence is relatively infrequent among 19 diverse families of RNA and DNA viruses, such that cross-species transmission plays a central role in virus evolution. Host jumping was especially common in viruses with RNA genomes, and by drawing broad-scale comparisons our analysis reveals which virus families have a greater propensity to jump species barriers and hence successfully emerge in new hosts. Finally, our data suggest that sampling more viruses increases the likelihood of detecting host jumping events.

## Introduction

Emerging pathogens that cross the species barrier to infect new hosts can profoundly affect human and animal health, as well as wildlife and the agricultural industries. Although most emerging diseases seemingly result from such a process of cross-species transmission, it is also the case that some viruses seem to rarely jump the species barrier and instead co-diverge with their hosts over long stretches of evolutionary time. For example, long-term virus-host co-divergence has been suggested to play a key role in the evolution of vertebrate herpesviruses over periods of ~400 million years [1] and insect baculoviruses over a time-scale of ~310 million years [2]. Indeed, it has been proposed that a number of families of DNA viruses have co-diverged with their hosts over long evolutionary time-scales [3–5], and do so more frequently than RNA viruses, which in contrast display a combination of co-divergence and host switching [6]. In particular, while phylogenetic trees for some RNA viruses, such as particular retroviruses, are generally congruent with those from their hosts suggesting long-term co-divergence [7], for others, such as flaviviruses, host jumping appears to be relatively frequent [8]. In the case of flaviviruses this likely in part reflects the fact that many are transmitted by arthropod vectors and characterized by short durations of infection. The situation appears to be even more complex in cases such as the hantaviruses where there is evidence of both co-divergence and host jumping [6].

Given the evolutionary and ecological barriers a virus must overcome to cross the species barrier and successfully establish itself in a new host, it might seem reasonable to assume that successful cross-species transmission is a relatively rare occurrence [9]. Indeed, many emerging diseases are in reality ‘spill-over’ infections, in which onward transmission between members of a new host species is limited such that extinction of the novel virus occurs rapidly [5]. Nevertheless, it is possible that an increased sampling of hosts and their viruses will reveal more instances of host jumping, in turn implying that cross-species transmission is a fundamental aspect of virus evolution [8]. As a case in point, although there is strong evidence that hepadnaviruses have co-diverged with their vertebrate hosts over hundreds of millions of years [10], the recent identification of hepadnaviruses in fish and amphibians has revealed more instances of cross-species transmission, potentially including that from aquatic to terrestrial vertebrates [11].

Clearly, identifying the relative frequencies of co-divergence versus cross-species transmission is of central importance to understanding the basic mechanisms of virus evolution and disease emergence. In particular, it is important to determine whether some virus families have a greater propensity to jump hosts than others and, if so, what factors govern this pattern. Currently, however, there is no quantitative or comparative measure of the frequency of cross-species transmission versus co-divergence, so that determining whether one virus family is more likely to jump species boundaries than another is difficult to assess. One simple and powerful way to estimate these key evolutionary parameters is via ‘co-phylogenetic’ analysis that assesses the degree of phylogenetic congruence (i.e. similarity) between hosts and their parasites [12]. In particular, a clear congruence between the host and virus phylogenies provides strong evidence for a history of co-divergence, whereas phylogenetic incongruence (i.e. discordance) is compatible with cross-species transmission.

To date, co-phylogenetic studies of viruses have largely focused on the evolution of a subset of viruses within a particular virus family, and have not been performed in a comparative manner. For example, although there has been much work dedicated toward describing co-divergence in herpesviruses, these studies generally only encompass one particular host type (e.g. primates [13]) and so may fail to capture the broader picture of potential host jumps among more distantly related species. Hence, there has been no attempt to use analyses of this kind to provide a broad-scale comparative and quantitative measure of the frequency of co-divergence and cross-species transmission in virus evolution. Herein, we provide such an analysis. Specifically, using a normalized tree topology distance metric based on the Penny and Hendy distance metric that enables comparisons between pairs of virus and host trees with different numbers of tips [14], which we now term the ‘nPH85’ distance (where n = normalized), we compare phylogenies of virus families and their hosts. While this method does not explicitly model host-switching events, it does provide a simple means to compare multiple topologies of virus-host pairs, and accounts for differences in sample size and the fact that several viruses from a specific family can infect a single host species.

**Fig 1:**
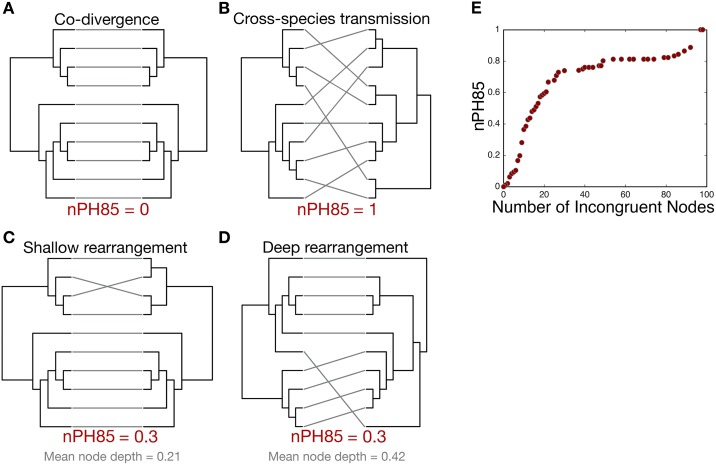
Tanglegrams of phylogenetic trees created using simulated data. Lines connect the virus with its respective host. Hence, if viruses and hosts have congruent phylogenies—indicative of strong virus-host co-divergence—then there will obviously be more horizontal than diagonal lines. Panel (A) illustrates a perfectly matched topology between virus and host trees and thus the nPH85 = 0. Panel (B) exemplifies an entirely mismatched topology between virus and host trees, where the nPH85 = 1. Data from viruses in nature will fall between these two extremes. Panels (C) and (D) illustrate two examples where the host trees have one incongruent node. Panel (C) corresponds to a shallower section of the tree than in panel (D), but the two nPH85 are the same, such that the position of the incongruence does not produce a systematic bias. Panel (E) elucidates the relationship between the nPH85 distance and the number of incongruent nodes between a pair of simulated trees with 100 tips.

To provide a quantitative measure of host switching we compared 19 virus families, incorporating viruses infecting a diverse sample of eukaryotic hosts including mammals, birds, reptiles, amphibians, fish, plants and insects. Under the measure we utilize here, when nPH85 = 0 between the virus and host trees it implies that their topologies are identical such that there is very strong evidence for co-divergence (Fig 1A). Conversely, if nPH85 = 1, there are no clades in common such that co-divergence is implausible (Fig 1B). Crucially, this metric does not depend on where the mismatched clades are located in the tree. For example, for a pair of virus and host trees that differ in one clade, the nPH85 is the same whether species jumping events were recent (i.e. shallow nodes Fig 1C) or ancient (i.e. deep nodes Fig 1D). Importantly, the nPH85 distance increases as the number of incongruent nodes (i.e. nodes that differ) between the virus and host trees increases (Fig 1E).

## Results

### A phylogenetic measure of the relative frequency of virus-host co-divergence

Our analysis considered a total of seven DNA and 12 RNA virus data sets that provided sufficient data to perform a quantitative co-phylogenetic analysis. Hence, the study relied heavily on specific selection criteria (see Materials and methods) that necessarily limited data availability. Despite these rigorous criteria, the majority of data sets encompassed a diverse collection of viruses and host species, and hence can be regarded as illustrative of the broad-scale frequency of co-divergence versus cross-species transmission. These data contained no evidence for recombination.

To determine the prevalence of host switching between different viruses, we inferred family-level viral phylogenies and compared these to phylogenies of their hosts. Importantly, our analytical approach—which utilizes the nPH85 distance—provides a relative measure of phylogenetic congruence that is directly comparable between data sets that differ in size (i.e. different number of viruses and host species). Our method assumes that viruses that have co-diverged with their hosts will share the same tree topology. In contrast, an increasing number of host jumping events should lead to greater phylogenetic incongruence. The reasoning behind this assumption is that there exists a very large number of possible phylogenetic tree topologies even for data sets with a few samples, such that similarities between a pair of virus-host trees (i.e. congruence) are highly unlikely to arise by chance. Of course, phylogenetic events other than cross-species transmission might also lead to phylogenetic incongruence and we test the validity of this assumption later in the manuscript.

**Fig 2:**
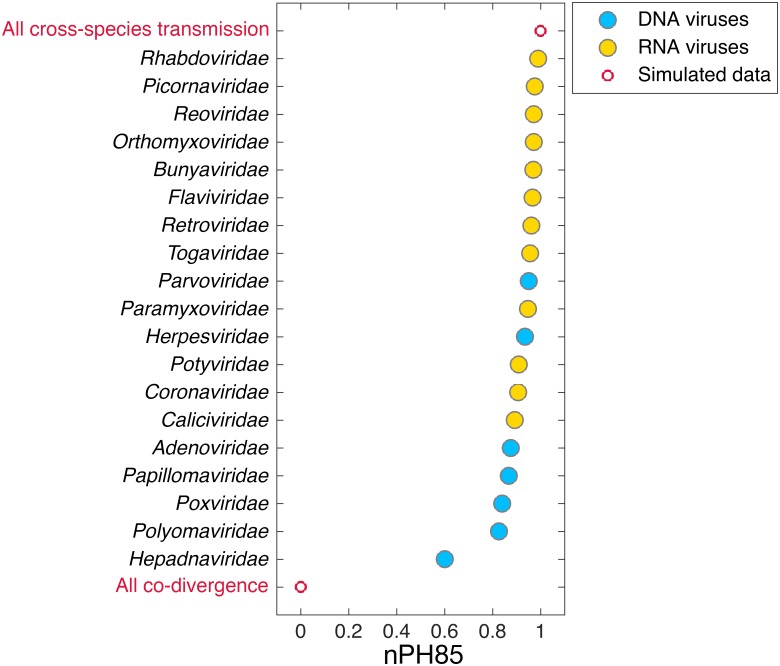
Overall normalized topological distance between two unrooted phylogenetic trees for each virus family by normalizing the Penny and Hendy [14] metric (i.e. nPH85). A range of DNA (blue) and RNA (yellow) virus families are shown. If nPH85 = 0, it is indicative of virus-host co-divergence, while nPH85 = 1 suggests frequent cross-species transmission (red). For ease of interpretation virus families are ranked by descending frequency of cross-species transmission.

Across the data set as a whole we found that all virus families displayed relatively large tree topological distances with nPH85 values of ≥0.6, suggesting that cross-species transmission is widespread, at least at the family-level (Fig 2; S3 Table). While all families showed distances at the upper end of the scale, the *Hepadnaviridae* (double-stranded DNA) had the shortest distance (nPH85 = 0.6), indicating that this family experiences more frequent co-divergence than any other studied here. At the other end of the spectrum both the *Rhabdoviridae* and *Picornaviridae* (single-stranded RNA) displayed nPH85 > 0.97, indicative of frequent host switching and hence little evidence for virus-host co-divergence.

**Fig 3:**
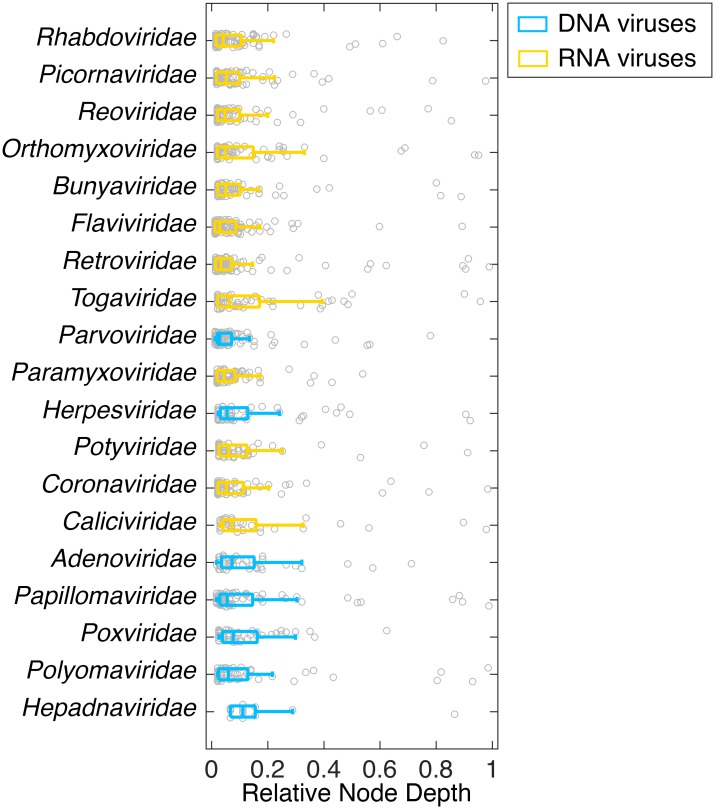
Relative node depths of incongruences between host and virus phylogenies showing the median and 25^th^ and 75^th^ percentiles (boxplots) as well as the raw data. A relative node depth close to 0 can be interpreted as the occurrence of host-switching events at the tips of the phylogenetic tree, whereas a relative node depth close to 1 suggests host-switching events at the root of the phylogenetic tree. A range of DNA (blue) and RNA (yellow) virus families are shown. For ease of interpretation virus families are ranked as in Fig 2.

We also investigated when the species jumping events occurred in the evolutionary history of the virus families. To do this, we determined whether phylogenetic incongruences tended to occur in deeper sections of the phylogeny or to more shallow nodes in the tree. Accordingly, we considered the number of nodes subtending clades in the host tree that are not present in the virus tree, a metric known as ‘node depth’. Nodes that are deep correspond to clades that are more diverse, and often older, than those clades subtended by shallower nodes. For each pair of virus-host trees we calculated the depth of every node that differed within each virus-host pair and divide each depth by the maximum node depth (Fig 3). This normalized metric, which we term ‘relative node depth’, ranges between near 0 for phylogenetic incongruences at shallow nodes, and 1 for incongruences at deeper nodes. Most incongruences corresponded to shallow nodes, which is expected because there are naturally more shallow nodes than deep nodes in phylogenetic trees. However, that incongruences were found in both shallow and deep nodes suggests that co-divergence is relatively rare in these virus families, even over long evolutionary time-scales.

**Fig 4:**
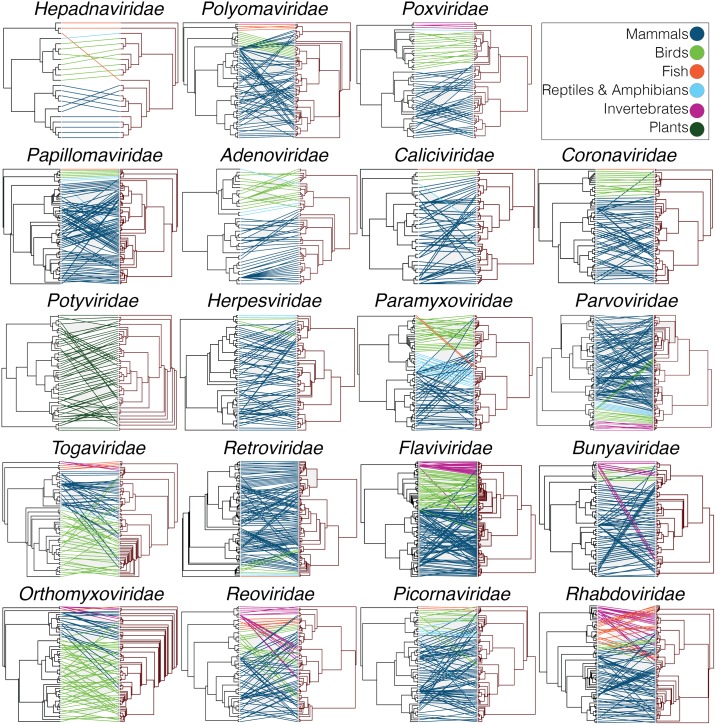
Tanglegrams of rooted phylogenetic trees for each virus family. Host trees were rooted first following their known phylogenetic history, with virus trees then rooted based on the host tree. The ‘untangle’ function was used to maximize the congruence between the host and virus phylogenies. Lines that connect the host (left) with its virus (right) are colored according to the host type (dark blue: mammals; light green: birds; light blue: reptiles and amphibians; red: fish; pink: invertebrates; dark green: plants). Phylogenies with the individual tip labels visible are shown in S1 Fig.

Tanglegrams depicting pairs of rooted phylogenetic trees display the evolutionary relationship between each virus family and their host species (Fig 4; phylogenies with the individual tip labels visible are shown in S1 Fig). Despite the obvious widespread occurrence of host jumping, a number of co-phylogenies reveal the occurrence of at least some co-divergence, as expected from the nPH85 distances. For example, the tanglegrams for the *Hepadnaviridae* and *Poxviridae* exhibit some clear matches with the evolutionary histories of their respective hosts. Most notably, their co-phylogenies show a clear segregation between distinct clades that are associated with a specific host type (mammals, birds, etc.). Conversely, the phylogenies of most RNA viruses appear to largely mismatch those of their hosts.

**Fig 5:**
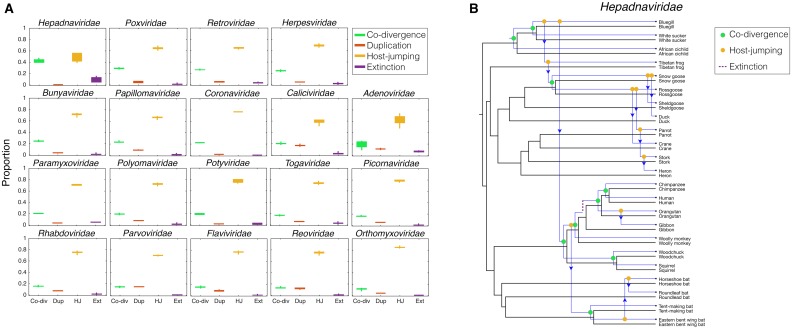
**(A)** Reconciliation analysis of each virus family using Jane [15]. Boxplots illustrate the range of the proportion of possible events. The ‘event costs’ associated with incongruences between trees were conservative towards co-divergence and defined here as: 0 for co-divergence, 1 for duplication, 1 for host-jumping and 1 for extinction. Virus families are ranked in order of highest mean co-divergence to lowest mean co-divergence. Abbreviations on the x-axis are as follows: ‘Co-div’ = co-divergence, ‘Dup’ = duplication, ‘HJ’ = host-jumping, ‘Ext’ = extinction. **(B)** Reconciliation of the *Hepadnaviridae* phylogeny with that of their vertebrate hosts, again utilizing the co-phylogenetic method implemented in Jane [15]. The figure illustrates all possible co-divergence, extinction and host-jumping events (no lineage duplication events were reconstructed in this case).

Our fundamental assumption is that incongruences between virus and host topologies imply the occurrence of cross-species transmission. To test the validity of this assumption, we reconciled the viruses with the phylogenetic history of their hosts. By associating ‘event costs’ with host-jumping, as well as with lineage duplication and extinction events, we found the range of optimal co-phylogenetic solutions for each virus family (Fig 5A). As with the analysis of topological distances, this revealed that cross-species transmission was the most common evolutionary event in all virus families studied here, with co-divergence consistently less frequent (with the possible exception of the *Hepadnaviridae*–see below), and lineage duplication and extinction playing a much more minor role. We next reconstructed the history of these evolutionary events in detail in the *Hepadnaviridae* (i.e. the most co-divergent virus family). This revealed that under the most likely co-phylogenetic scenario the proportion of cross-species transmission represents 0.57 of all events (i.e. co-divergence = 9 events; duplications = 0; extinction = 1; host-jumping = 13; Fig 5B). Since the nPH85 distance for the hepadnavirus data set was 0.6, we suggest that our method generates results consistent with the reconciliation analysis. In addition, one important disadvantage of performing full reconciliation analysis is that co-phylogenetic methods such as that implemented in Jane [15] and Tarzan [16] are not straightforward since they offer many combinations of possible events and are difficult to compare between families, especially in cases with more than ~50 viruses where there are many possible co-phylogenetic scenarios. Despite these limitations, our reconciliation analysis did reveal the possible causes of the topological incongruence between the virus and host phylogenies.

### Correlates of cross-species transmission and co-divergence

**Fig 6:**
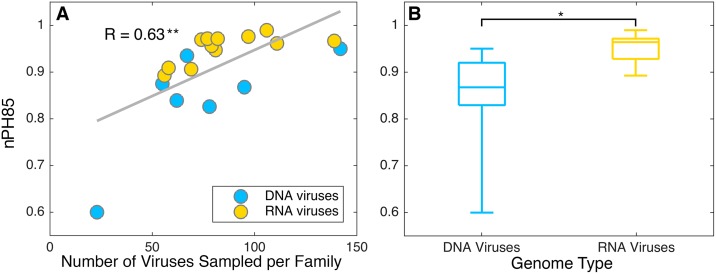
**(A)** The nPH85 distance as a function of the number of viruses per virus family. Pearson’s correlation coefficient, R, was found to be statistically significant (p<0.005). **(B)** nPH85 distances by genome type showing the median (horizontal line) and 25^th^ and 75^th^ percentiles. A t-test showed that the difference between these distances was significant (p<0.05). As before, a range of DNA (blue) and RNA (yellow) virus families are shown.

We next determined whether there was any association between the relative frequency of co-divergence and larger scale biological properties, such as the number of viruses per family and whether the viruses in question possess RNA or DNA genomes. To better display this analysis branches on the co-phylogenetic trees were colored according to host type, which comprised mammals, fish, birds, reptiles, amphibians, invertebrates, and plants (Fig 4), such that each co-phylogeny incorporated between one (i.e. *Potyviridae*) and five (i.e. *Togoviridae*) host types. Notably, we found a significant association between the number of viruses per virus family and the nPH85 (p<0.005) (Fig 6A). Importantly, because we expect no association between the number of viruses and hosts per family and the nPH85 under our tree distance metric, this result implies that sampling more viruses increases the likelihood of detecting host jumping events. In addition, we found that DNA viral families had, on average, a shorter nPH85 distance than families of RNA viruses (p<0.05) (Fig 6B). Note that there is no significant difference (p = 0.5) between the number of viruses in families of DNA viruses compared to those in RNA virus families. In this context it is striking that the five families with the shortest topological distances all possessed DNA genomes. This analysis also revealed that segmented viruses had a significantly larger nPH85 distance than non-segmented viruses (p<0.05), and that negative-sense RNA viruses had a larger nPH85 distance than positive-sense RNA viruses (p<0.005); however, the sample sizes within all these categories were small so that these results should be treated with caution. Finally, we note that although the duration of infection (for example, the division between acute versus chronic infections) is clearly a parameter that would likely affect the frequency of host jumping [3, 5], we were unfortunately unable to perform any analyses of this variable on the data available here as it tends to be host-specific rather than a general characteristic of individual virus families.

## Discussion

Understanding how viruses and their hosts co-evolve is central to revealing the nature of virus evolution and the determinants of disease emergence. In particular, we lack a quantitative understanding of whether some types of virus, such as those classified into different families or that possess genomes of different nucleic acid types, are better able to jump species boundaries compared to others. To investigate the comparative prevalence of cross-species transmission among viruses we measured the congruence between virus and host phylogenetic trees using a normalized tree topological distance-based approach (nPH85, [14]). If taxonomically related viruses have an evolutionary history of co-divergence with their hosts the virus and host phylogenetic trees should be similar in topology, whereas phylogenetic incongruence is the signature of species jumping. Overall, our analysis revealed absolute departure from co-divergence among all the virus families studied here (nPH85 ≥ 0.6 and supported by the reconciliation analysis) suggesting that cross-species transmission occurs frequently, at least at the level of virus family. Particularly striking was that even the most slowly evolving DNA viruses, which have previously been suggested to represent exemplars of virus-host co-divergence [1], exhibit relatively common cross-species transmission. Hence, at their most basic, these results indicate that viruses are often exposed to a variety of susceptible host species that provide opportunities for cross-species transmission.

Despite the overall large nPH85 distances observed among all virus families, our data also revealed that the *Hepadnaviridae*, *Polyomaviridae*, *Poxviridae*, *Papillomaviridae* and *Adenoviridae* had the shortest nPH85 distances and were thus relatively more host-specific than the other virus families analyzed here. This is supportive of earlier suggestions that some DNA viruses have a long history of co-divergence with their hosts [4], which in some cases may be a reflection of relatively long durations of infection. Indeed, long-term virus-host associations have been observed in the *Herpesviridae* [1], the *Poxviridae* [17] and the *Polyomaviridae* [18]. However, it is also important to note that we found these viruses contain more instances of host jumping than previously thought. For example, although the tanglegram shown in Fig 4 suggests co-divergence in the case of some primate hepadnaviruses, cross-species transmission seemingly occurs more frequently among those hepadnaviruses that infect birds. In addition, it was recently observed that a fish (bluegill) hepadnavirus clusters more closely with mammalian hepadnaviruses than to other fish viruses [11] (see Figs 4 and 5B). Similarly, early studies of RNA viruses suggested that virus-host co-divergence was important in the evolution of two members of the *Flaviviridae* that infect primates—the pegiviruses and hepaciviruses, [19–21]. However, more recent phylogenetic analyses of expanded data sets have revealed multiple cross-species transmissions events, including the recent emergence of hepaciviruses in domestic dogs, horses and donkeys [22], and a newly described pegiviruses in rodents, bats and horses [23].

Despite the obvious caveat of sample size, it seems that RNA viruses generally experience more frequent cross-species transmission than their DNA counterparts. Indeed, the RNA viral families analyzed here had an overall mean nPH85 distance of 0.95, compared to DNA viruses with a mean of 0.84. This may, in part, be due to the fact that RNA viruses are generally characterized by very high rates of mutation and replication [24]. Intuitively, high rates of evolutionary change should confer more rapid adaptation to new environments, which, coupled with the frequency of exposure to new hosts, will facilitate host-switching. In addition, many RNA viruses are characterized by short durations of infection that will limit the opportunities for virus-host co-divergence [4]. An informative exception among RNA viruses are the simian foamy viruses (SFV), in which hosts may develop long-term latent infections and the virus has been associated with long-term co-divergence [25]. Indeed, it is notable that among the *Retroviridae* analyzed here those assigned to SFV seem to display relatively similar evolutionary histories to those of their primate hosts (see S1 Fig).

It is also possible that successful cross-species transmission occurs more frequently among phylogenetically related hosts, likely because it is easier to infect and replicate in genetically similar hosts that share less divergent cell receptors [26]. In addition, related hosts may sometimes inhabit the same geographic region, increasing the probability of cross-species transmission through more frequent exposure [13]. Indeed, a useful generality in studies of disease emergence is that the closer the phylogenetic relationship between hosts, then, given appropriate exposure, the more likely that a pathogen will be able to jump between them, in turn leading to preferential host switching [27]. If true, so that cross-species transmission results in a viral phylogeny that mirrors that of their hosts, then any phylogeny-based approach such as that utilized here will underestimate the true frequency of host jumping. As a case in point, although there is a general concordance between the phylogenies of simian immunodeficiency virus (SIV) and their primate hosts, in which four species of African green monkey harbor distinct forms of SIV that is clearly suggestive of co-divergence [19], it has been argued that the evolutionary history of SIV may also have been shaped by preferential host switching [28], although these mechanisms are not mutually exclusive. In contrast, incomplete lineage sorting among closely related viruses may produce a false signal for cross-species transmission when co-divergence has in fact occurred [19]. In addition, because there is growing evidence that viruses can have complex evolutionary histories with genes derived from multiple sources [29], it is important to note that our virus phylogenies are necessarily gene trees rather than species trees. It is therefore possible that other virus gene trees will exhibit a stronger topological match with host phylogenies than those presented here, and hence provide more evidence for co-divergence. Finally, while our analysis was only based on robust phylogenetic patterns, because nodes that were topologically uncertain were excluded from the analysis, it is possible that our virus trees contain topological errors reflecting the use of sometimes small numbers of highly divergent sequences.

Another important aspect of assessing virus-host co-divergence is that the evolutionary time-scales of viruses and their hosts are consistent [30]. Although such a comparison is valuable, it is problematic for the present study because high rates of evolution lead to substitutional saturation in virus genomes at a much faster rate than in cellular organisms. Indeed, it is likely that many of the cross-species transmission events implied here have occurred on time-scales of many millions of years. As a result, temporal signal is rapidly lost, precluding accurate estimates of their long-term evolutionary time-scales, even though the topology is often accurately recovered [31]. We therefore suggest that simpler topological comparisons such as those performed here may be a more informative way to proceed in family-level studies of cross-species transmission versus co-divergence.

Overall, we have observed frequent cross-species transmission across the virus families studied here, with relatively little evidence for virus-host co-divergence. Hence, our study suggests that, at the virus family scale in the data analyzed here, host switching plays a major role in the evolution and diversification of viruses and, importantly, that it can occur in viruses of all types. Interestingly, we found that increased sampling of viruses from different host species reveals more frequent species jumping events among viral families. As such, the discovery of new viruses is likely to reveal more instances of cross-species transmission. Undoubtedly, the analysis presented here should be extended to a wider range of data sets as they become available, particularly because increased taxon sampling results in a larger tree space and increases the statistical power of these analyses.

## Materials and methods

### Data collection

Gene sequence data of viruses were obtained from GenBank (Table 1; see S1 Table for all GenBank accession numbers). Following a broad and comprehensive survey of all virus genomic data available on GenBank, a total of 19 family-level virus data sets passed our selection criteria and were included in the analysis. These selection criteria, which are independent of whether the viruses have evolved by co-divergence or cross-species transmission, were: (i) the availability of virus sequence data that included a wide range of distinct and diverse virus species that is representative of the virus genera currently available; (ii) the availability of data with informative genomic regions that can be used to reveal evolutionary relationships (e.g. the RNA-dependent RNA polymerase—see Table 1) and that were not so divergent as to prevent reliable sequence alignment; and (iii) the virus sequence data met a minimum length requirement of 100 amino acids following alignment and the removal of any ambiguously aligned regions. The virus families that passed these selection criteria were the *Adenoviridae*, *Bunyaviridae*, *Caliciviridae*, *Coronaviridae*, *Flaviviridae*, *Hepadnaviridae*, *Herpesviridae*, *Orthomyxoviridae*, *Papillomaviridae*, *Paramyxoviridae*, *Parvoviridae*, *Picornaviridae*, *Polyomaviridae*, *Potyviridae*, *Poxviridae*, *Reoviridae*, *Retroviridae*, *Rhabdoviridae* and *Togaviridae*. Each data set contained between 23–142 viruses from a diverse range of eukaryotic hosts, including mammals, birds, reptiles, amphibians, fish, invertebrates, and plants. For the purposes of this study we regarded a virus isolated from a particular host species as a distinct virus sample worthy of analysis: for example, rabies virus isolated from a human host was deemed distinct from rabies virus isolated from a canine host. The resulting virus and host data sets included in this study comprised a diverse sample of the available data (see S2 Table for a summary of the virus and host diversity). Most data sets contained more viruses than those from their corresponding hosts because they included multiple viruses from a family that can infect the same host.

**Table 1:** Summary of the virus data used in this study. The best-fit amino acid substitution models were selected according to the Bayesian Information Criterion.

Virus Family	Genome Type	Genetic Region for Phylogenetic Analysis	Number of Sequences in Data Set	Amino Acid Sequence Length Range Before Trimming	Amino Acid Sequence Length Range After Trimming	Amino Acid Substitution Model
***Adenoviridae***	DNA	Polymerase	55	843–1341	381–484	LG+I+Γ
***Bunyaviridae***	RNA	RdRp	74	149–4050	113–834	LG+Γ+F
***Caliciviridae***	RNA	RdRp	56	150–2357	113–709	LG+I+Γ
***Coronaviridae***	RNA	RdRp	69	210–2733	210–1757	LG+I+Γ+F
***Flaviviridae***	RNA	Polyprotein (contains polymerase)	139	496–3993	159–1165	LG+I+Γ+F
***Hepadnaviridae***	DNA	Polymerase	23	601–899	528–612	LG+I+Γ+F
***Herpesviridae***	DNA	Polymerase	67	155–1247	151–622	LG+Γ
***Orthomyxoviridae***	RNA	PB1 subunit	77	708–777	374–417	LG+Γ
***Papillomaviridae***	DNA	E1 gene	95	444–693	335–405	LG+I+Γ+F
***Paramyxoviridae***	RNA	Large polymerase	81	145–2501	145–1680	LG+Γ+F
***Parvoviridae***	DNA	VP1	142	145–991	116–341	LG+Γ
***Picornaviridae***	RNA	Polyprotein (contains polymerase)	97	398–2816	213–1385	LG+I+Γ+F
***Polyomaviridae***	DNA	VP1	78	277–505	261–379	LG+Γ+F
***Potyviridae***	RNA	NIb polyprotein (contains RdRp)	58	258–355	197–221	LG+Γ
***Poxviridae***	DNA	Polymerase	62	178–1190	178–672	LG+Γ+F
***Reoviridae***	RNA	RdRp (λ3 and VP1)	82	642–1435	274–550	LG+I+Γ
***Retroviridae***	RNA	Pol	111	137–1246	124–863	RtREV+I+Γ
***Rhabdoviridae***	RNA	Large polymerase	106	889–2196	383–786	LG+Γ+F
***Togaviridae***	RNA	Non-structural polyprotein (contains RdRp)	79	1637–2593	1036–1103	LG+Γ

### Phylogenetic analysis

For each virus family nucleotide sequences were first translated to amino acid data using Seqotron v.1.0.1 [32], aligned with MUSCLE v.3.8 [33], and poorly aligned regions then eliminated using trimAl [34], ensuring that all remaining sequences were at least 100 amino acids in length (Table 1). Amino acid sequences were aligned because there is widespread substitutional saturation at the nucleotide level. Although our data sets utilize single genes, we ensured that they were free of inter-specific virus recombination using RAT [35].

To estimate phylogenetic trees for the virus data sets we selected the optimal amino acid substitution model identified using the Bayesian Information Criterion as implemented in Modelgenerator v0.85 [36] and analyzed the data using PhyML v3.1 [37], employing the SPR branch-swapping tree search algorithm (see Table 1 for the substitution models used). We assessed the support for individual nodes using the approximate likelihood ratio test (aLRT) implemented in PhyML v3.1 [38], with aLRT values ranging between 0 (no support) and 1 (strong support). Studies involving simulations and empirical data have demonstrated that this statistic has similar false-positive rates to other metrics, such as the non-parametric bootstrap [39].

Cladograms were constructed for all host species from which the viruses of interest were isolated. In each case the host tree topologies used were the most up-to-date available in the literature [40–44]. For the vector-borne viruses studied here, in which viruses pass between arthropods and vertebrates, the appropriate vertebrate species were assigned as the hosts. In contrast, for insect-specific viruses, where there is no evidence for vertebrate involvement, the relevant invertebrate species were assigned as the hosts. Since there were often multiple viruses that infected the same host species, multiple lineages within a single host (i.e. polytomies) were added to the host phylogenetic tree to ensure the number of hosts equaled that of the virus tree. The addition of these polytomies does not influence the nPH85 distance metric (described in detail below) because the distance between a polytomous clade and one that is fully resolved is zero [14].

All virus and host phylogenetic trees and virus sequence alignments are available at github.com/jemmageoghegan.

### Analysis of virus-host co-divergence

We measured the extent of virus-host co-divergence (and by exclusion host-jumping) by comparing, in a quantitative manner, the tree topologies for viruses and their corresponding hosts. To this end we calculated a normalized PH85 tree topological distance [14], referred to here as the ‘nPH85’ distance (this function has been included in NELSI v0.1 [45]). Specifically, the nPH85 distance, which utilizes two phylogenetic trees as its input, describes the number of bipartitions (clades) that are not shared between two tree topologies. Importantly, it does not depend on the nodes where the topological differences occur in the tree (Fig 1). In addition, this metric considers the tree topology of unrooted trees, but not the branch lengths of the tree. First, the PH85 metric is calculated as the topological distance between a pair of unrooted trees. It can be understood in terms of the following:

$$\left({\text{ T}}_{1}\cap {\text{T}}_{2}\right)\prime ,$$

where T_1_ and T_2_ are the clades contained within the host and virus trees, respectively. Let the expression T_1_ ∩ T_2_ denote the clades that are shared between both trees so that (T_1_ ∩ T_2_)′ corresponds to the clades that are not shared between the pair (i.e. those that are unique to each tree). The actual PH85 distance is twice the number of unique clades. To normalize this metric we divide PH85 by the maximum distance by considering the two tree topologies, randomizing the tips for one of the trees 1000 times, and calculating PH85 for each replicate (where 1000 randomizations was shown to be robust even for very large trees; see S2 Fig). The largest value of the 1000 randomizations is approximately the maximum PH85 distance in tree topologies. Therefore, nPH85 ranges between 0, for identical trees, and 1, for trees that have no clades in common (Fig 1). The advantages of this method over other tree distance metrics is that it is comparable for pairs of trees with different numbers of tips, it maintains the backbone of the tree (i.e. the tree structure remains constant, unlike in [46]), and it is comparable for trees with polytomous nodes. To address phylogenetic uncertainty, we collapsed all nodes with aLRT of less than 0.8, which corresponds to a false-positive rate of <0.1 [39]. In such cases, we randomly resolved the polytomies 100 times and calculated the nPH85. Accordingly, we report the overall normalized topology distance, as well as the mean and 95% percentile range of values (S3 Table).

To determine whether host jumping occurred more often toward the root or tips of the trees, we calculated the relative node depth for incongruent nodes between virus-host pairs of trees (see Fig 1C and 1D). This metric counts the number of nodes contained within each clade in the host tree that are not present in the virus tree. Because this number can depend on the size of the tree, we divide each of the node depths by the largest value in the tree. Accordingly, this metric is decreased if incongruent clades correspond to shallow nodes (Fig 1C) compared to deep nodes (Fig 1D). For example, the maximum node depth is 1 if a pair of trees differs in the deepest node and approaches 0 if they differ only in very shallow nodes.

An important assumption of the current study is that incongruence between virus and host topologies is a result of cross-species transmission. In some instances, however, it might be possible to explain the lack of virus-host co-evolutionary history through multiple instances of lineage duplication and extinction, without such host-switching events. To address this issue, we reconciled the co-phylogenetic relationship between viruses and their hosts. In particular, we determined the optimal solutions for co-phylogenetic reconstruction for all families, including the possibility of lineage duplication and extinction, using the Jane co-phylogenetic software package [15]. This uses a polynomial time dynamic programming algorithm in conjunction with a genetic algorithm to find optimal solutions to reconcile co-phylogenies. Although this is a simple heuristic method, it is able to generate results on relatively large data sets (although it is most effective for trees with less that ~40–50 tips). Importantly, we used ‘event costs’ associated with incongruences between trees that were conservative towards co-divergence and defined here as: 0 for co-divergence, 1 for duplication, 1 for host-jumping and 1 for extinction. Utilizing this reconciliation, we also examined the evolution of the *Hepadnaviridae* in more detail as this family contains the best evidence for co-divergence (see Results).

Finally, to assist in visualization of these data, tanglegrams for each virus family were constructed using TreeMap v3.0 [47]. Lines between the trees connect the host (left) with its virus (right). We utilized the ‘untangle’ function, which rotates the branches of one tree, to minimize the number of crosses lines. If viruses and hosts have congruent topologies then the number of crossed lines, and hence cross-species transmission events, will obviously be reduced.

## Supporting information

S1 Fig The same co-phylogenies as depicted in Fig 4 with the individual taxon labels visible. Common names for host species are used and virus names identify the host where appropriate.
(PDF)

S2 Fig The number of randomizations required to obtain the maximum topological distance (black lines) for the *Hepadnaviridae* and the *Parvoviridae* phylogenies, which represent the minimum and maximum number of viruses in our data sets, respectively. The red, dashed line illustrates the PH85 distance of the non-randomized data, while the black, solid line is the PH85 distance after randomizing the data after *n* randomizations.
(TIF)

S1 Table GenBank accession numbers for the virus and host genetic sequence data utilized here. (DOCX)

S2 Table Summary of the virus and host diversity included and excluded in this study. Virus genera were excluded either due to lack of available data or because we were unable to obtain a reliable alignment of sufficient length for phylogenetic analysis (i.e. at least 100 amino acids after trimAl pruning).
(DOCX)

S3 Table Overall nPH85 distances, means and 95% percentiles between two unrooted phylogenetic trees for each virus family determined using the normalized Penny and Hendy [14] topological distance method, implemented in in NELSI v0.1 [45]. The overall nPH85 distances are illustrated in Fig 2 in the main text.
(DOCX)
