# Supplementary material for: Comparative analysis estimates the relative frequencies of co-divergence and cross-species transmission within viral families
Source: PLoS Pathog. 2017 Feb 8;13(2):e1006215. doi: 10.1371/journal.ppat.1006215 (PMC5319820; doi:10.1371/journal.ppat.1006215)

Adenoviridae

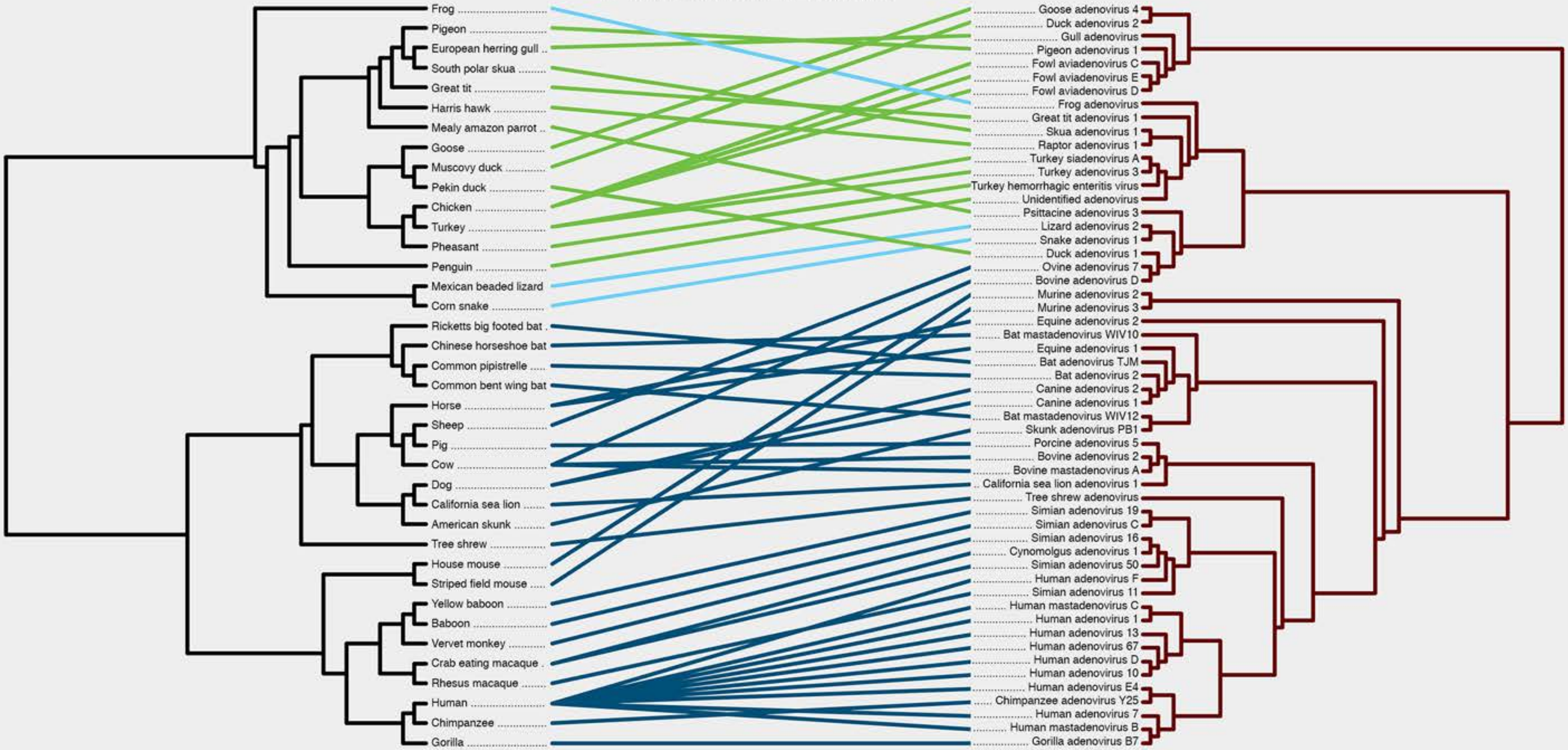

Bunyaviridae

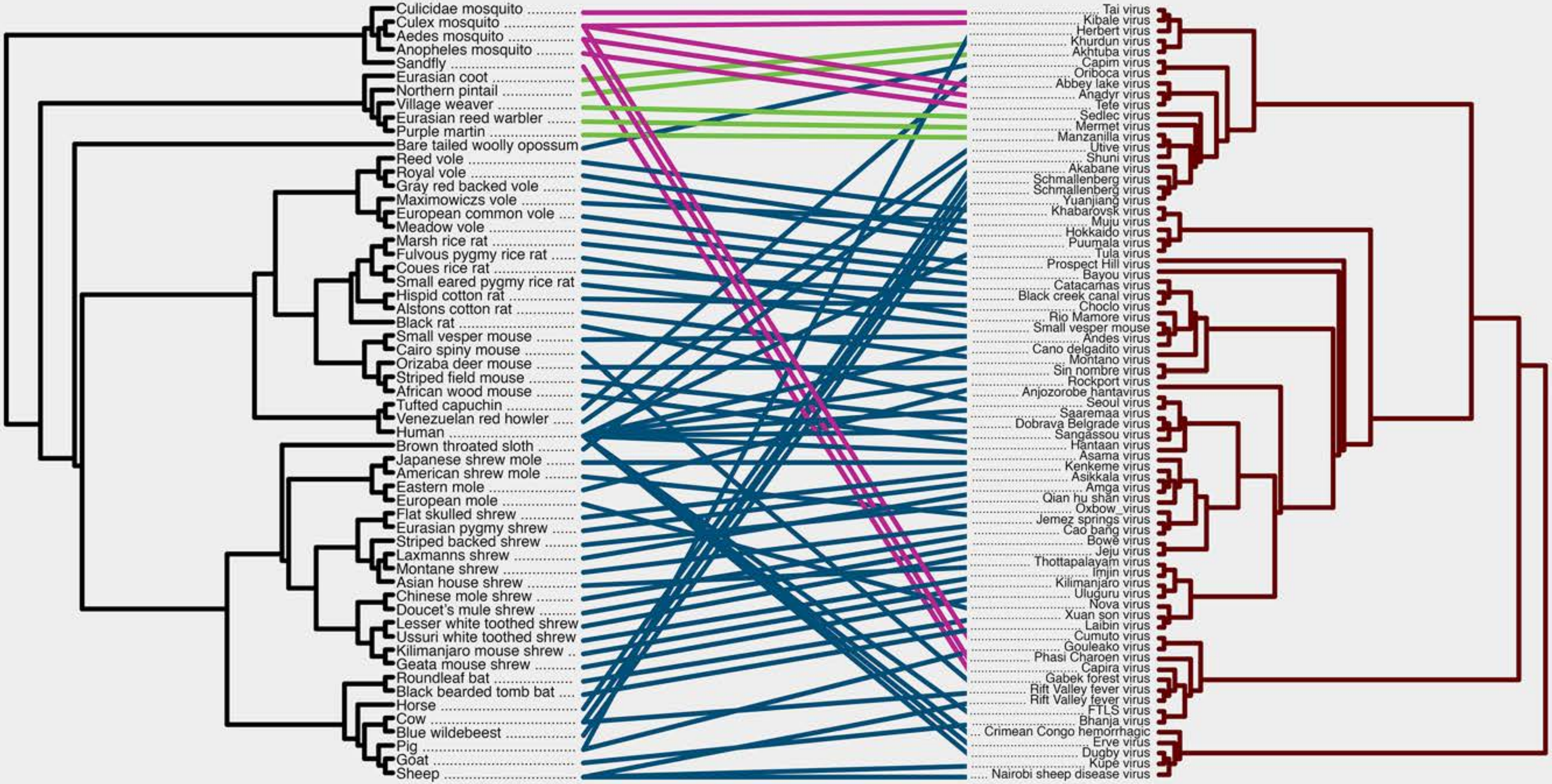

Caliciviridae

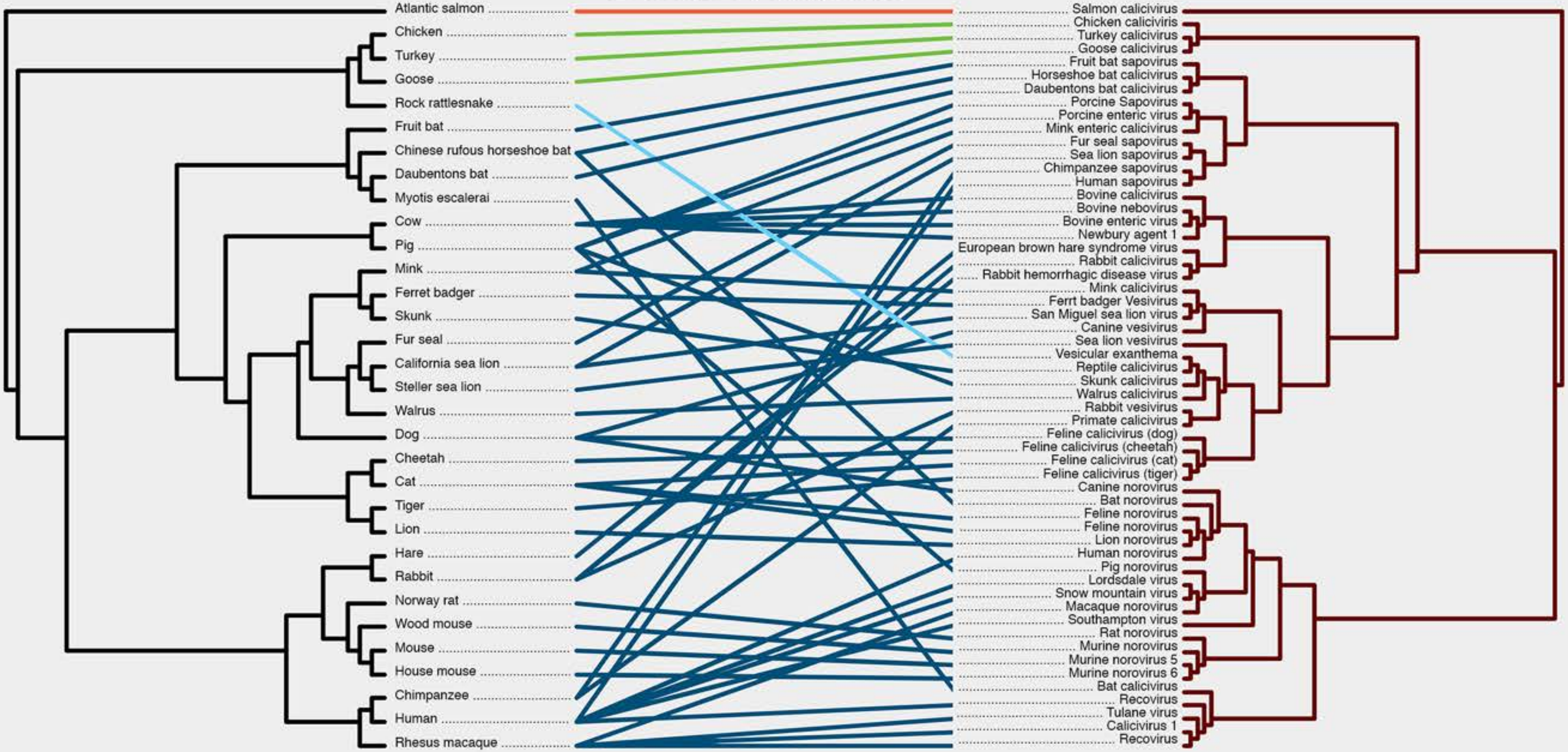

Coronaviridae

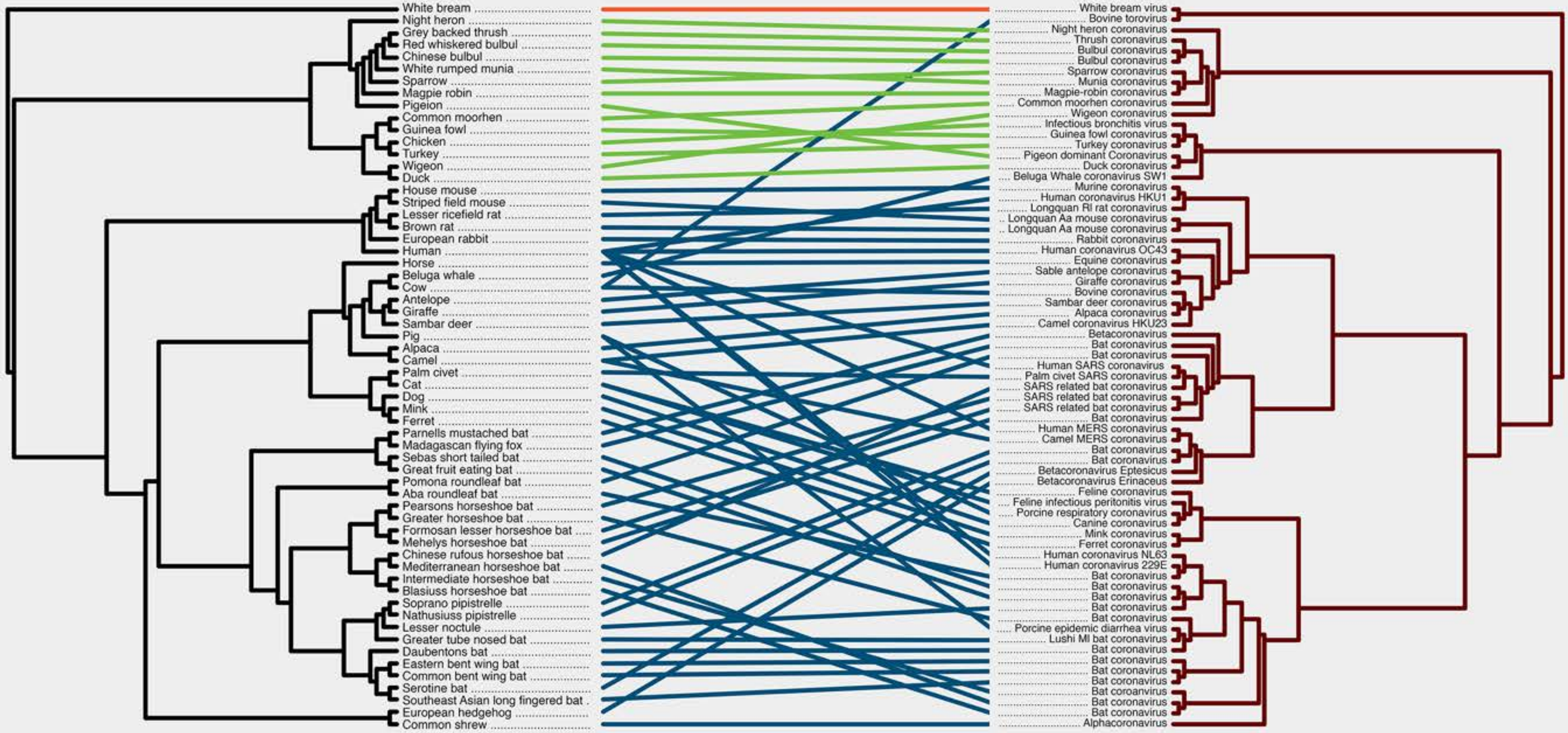

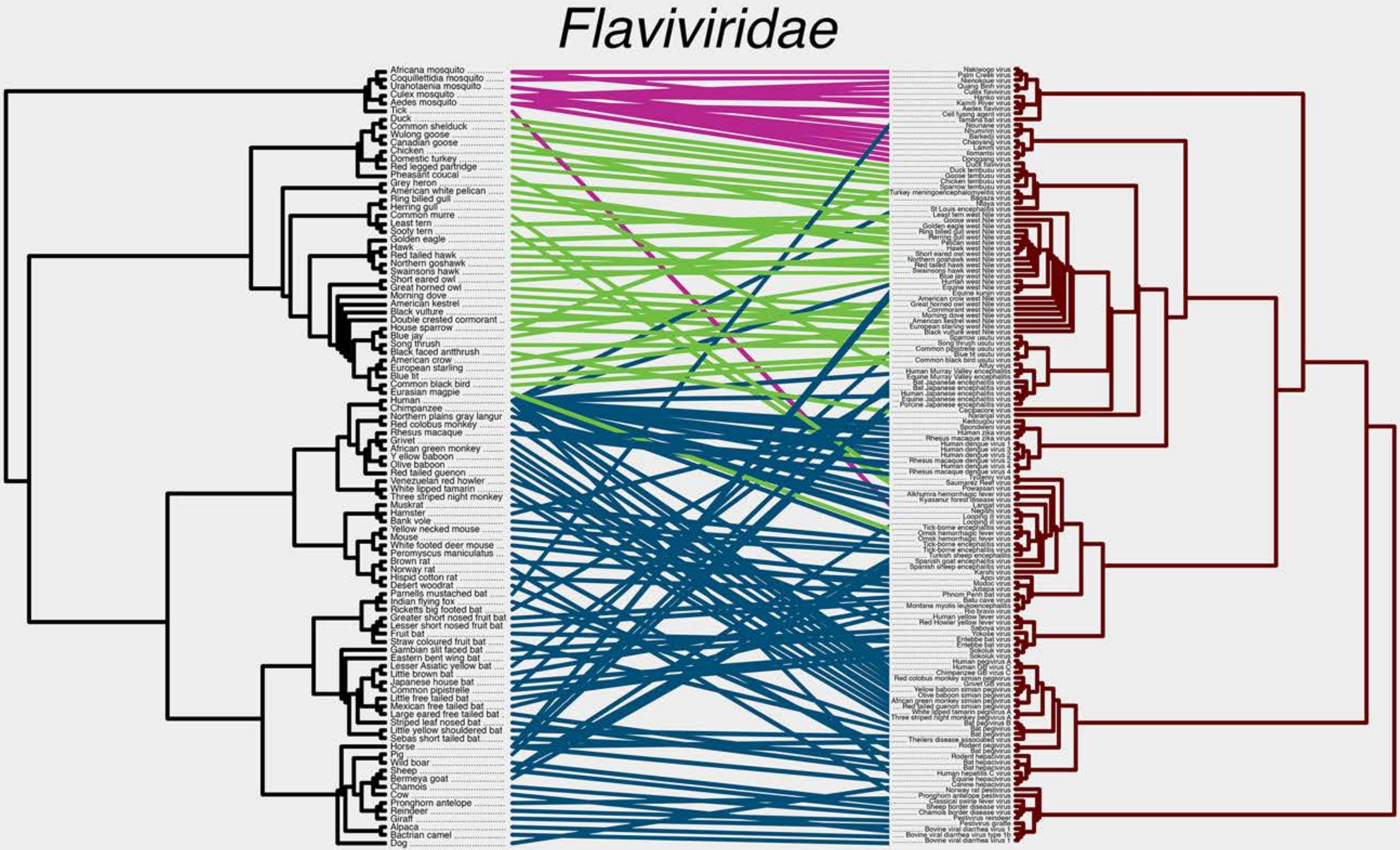

Flaviviridae
Africana mosquito
Coquillettidia mosquito
Uranotaenia mosquito
Culex mosquito
Aedes mosquito
Tick
Duck
Common shelduck
Wulong goose
Canadian goose
Chicken
Domestic turkey
Red legged partridge
Pheasant coucal
Grey heron
American white pelican
Ring billed gull
Herring gull
Common murre
Least tern
Sooty tern
Golden eagle
Hawk
Red tailed hawk
Northern goshawk
Swainsons hawk
Short eared owl
Great horned owl
Morning dove
American kestrel
Black vulture
Double crested cormorant
House sparrow
Blue jay
Song thrush
Black faced antthrush
American crow
European starling
Blue tit
Common black bird
Eurasian magpie
Human
Chimpanzee
Northern plains gray langur
Red colobus monkey
Rhesus macaque
Grivet
African green monkey
Y ellow baboon
Olive baboon
Red tailed guenon
Venezuelan red howler
White lipped tamarin
Three striped night monkey
Muskrat
Hamster
Bank vole
Yellow necked mouse
Mouse
White footed deer mouse
Peromyscus maniculatus
Brown rat
Norway rat
Hispid cotton rat
Desert woodrat
Parnells mustached bat
Indian flying fox
Ricketts big footed bat
Greater short nosed fruit bat
Lesser short nosed fruit bat
Fruit bat
Straw coloured fruit bat
Gambian slit faced bat
Eastern bent wing bat
Lesser Asiatic yellow bat
Little brown bat
Japanese house bat
Common pipistrelle
Little free tailed bat
Mexican free tailed bat
Large eared free tailed bat
Striped leaf nosed bat
Little yellow shouldered bat
Sebas short tailed bat
Horse
Pig
Wild boar
Sheep
Bermeya goat
Chamois
Cow
Pronghorn antelope
Reindeer
Giraff
Alpaca
Bactrian camel
Dog
Nakiwogo virus
Palm Creek virus
Nienokoue virus
Quang Binh virus
Culex flavivirus
Hanko virus
Kamiti River virus
Aedes flavivirus
Cell fusing agent virus
Tamana bat virus
Nounane virus
Nhumirim virus
Barkedji virus
Chaoyang virus
Lammi virus
Ilomantsi virus
Donggang virus
Duck flavivirus
Duck tembusu virus
Goose tembusu virus
Chicken tembusu virus
Sparrow tembusu virus
Turkey meningoencephalomyelitis virus
Bagaza virus
Ntaya virus
St Louis encephalitis virus
Least tern west Nile virus
Goose west Nile virus
Golden eagle west Nile virus
Ring billed gull west Nile virus
Herring gull west Nile virus
Pelican west Nile virus
Hawk west Nile virus
Short eared owl west Nile virus
Northern goshawk west Nile virus
Red tailed hawk west Nile virus
Swainsons hawk west Nile virus
Blue jay west Nile virus
Human west Nile virus
Equine west Nile virus
Equine kunjin virus
American crow west Nile virus
Great horned owl west Nile virus
Cormorant west Nile virus
Morning dove west Nile virus
American kestrel west Nile virus
European starling west Nile virus
Black vulture west Nile virus
Sparrow usutu virus
Song thrush usutu virus
Common pipistrelle usutu virus
Blue tit usutu virus
Common black bird usutu virus
Alfuy virus
Human Murray Valley encephalitis
Equine Murray Valley encephalitis
Bat Japanese encephalitis virus
Bat Japanese encephalitis virus
Human Japanese encephalitis virus
Equine Japanese encephalitis virus
Porcine Japanese encephalitis virus
Cacipacore virus
Naranjal virus
Kedougou virus
Spondweni virus
Human zika virus
Rhesus macaque zika virus
Human dengue virus 1
Human dengue virus 3
Human dengue virus 2
Rhesus macaque dengue virus 2
Human dengue virus 4
Rhesus macaque dengue virus 4
Tyuleniy virus
Saumarez Reef virus
Powassan virus
Alkhumra hemorrhagic fever virus
Kyasanur forest disease virus
Langat virus
Negishi virus
Looping ill virus
Looping ill virus
Tick-borne encephalitis virus
Omsk hemorrhagic fever virus
Omsk hemorrhagic fever virus
Tick-borne encephalitis virus
Tick-borne encephalitis virus
Turkish sheep encephalitis
Spanish goat encephalitis virus
Spanish sheep encephalitis virus
Karshi virus
Apoi virus
Modoc virus
Jutiapa virus
Phnom Penh Bat virus
Batu cave virus
Montana myotis leukoencephalitis
Rio bravo virus
Human yellow fever virus
Red Howler yellow fever virus
Saboya virus
Yokose virus
Entebbe bat virus
Entebbe bat virus
Sokoluk virus
Sokoluk virus
Human pegivirus A
Human GB virus C
Chimpanzee GB virus C
Red colobus monkey simian pegivirus
Grivet GB virus
Yellow baboon simian pegivirus
Olive baboon simian pegivirus
African green monkey simian pegivirus
Red tailed guenon simian pegivirus
White lipped tamarin pegivirus A
Three striped night monkey pegivirus A
Bat pegivirus B
Bat pegivirus
Bat pegivirus
Theilers disease associated virus
Rodent pegivirus
Bat pegivirus
Rodent hepacivirus
Bat hepacivirus
Bat hepacivirus
Human hepatitis C virus
Equine hepacivirus
Canine hepacivirus
Norway rat pestivirus
Pronghorn antelope pestivirus
Classical swine fever virus
Sheep border disease virus
Chamois border disease virus
Pestivirus reindeer
Pestivirus giraffe
Bovine viral diarrhea virus 1
Bovine viral diarrhea virus type 1b
Bovine viral diarrhea virus 1

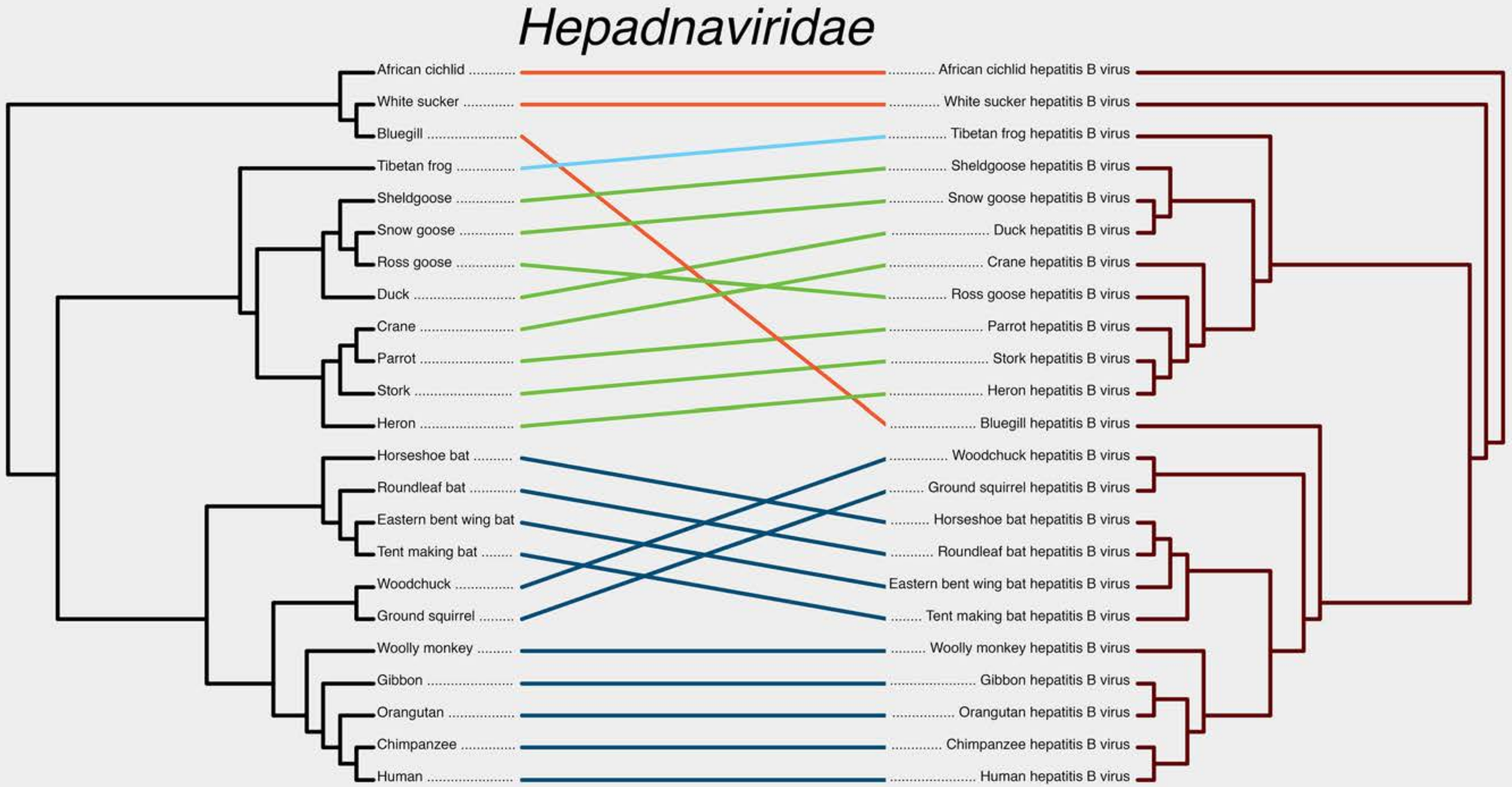

Hepadnaviridae
African cichlid
White sucker
Bluegill
Tibetan frog
Sheldgoose
Snow goose
Ross goose
Duck
Crane
Parrot
Stork
Heron
Horseshoe bat
Roundleaf bat
Eastern bent wing bat
Tent making bat
Woodchuck
Ground squirrel
Woolly monkey
Gibbon
Orangutan
Chimpanzee
Human
African cichlid hepatitis B virus
White sucker hepatitis B virus
Tibetan frog hepatitis B virus
Sheldgoose hepatitis B virus
Snow goose hepatitis B virus
Duck hepatitis B virus
Crane hepatitis B virus
Ross goose hepatitis B virus
Parrot hepatitis B virus
Stork hepatitis B virus
Heron hepatitis B virus
Bluegill hepatitis B virus
Woodchuck hepatitis B virus
Ground squirrel hepatitis B virus
Horseshoe bat hepatitis B virus
Roundleaf bat hepatitis B virus
Eastern bent wing bat hepatitis B virus
Tent making bat hepatitis B virus
Woolly monkey hepatitis B virus
Gibbon hepatitis B virus
Orangutan hepatitis B virus
Chimpanzee hepatitis B virus
Human hepatitis B virus

Herpesviridae

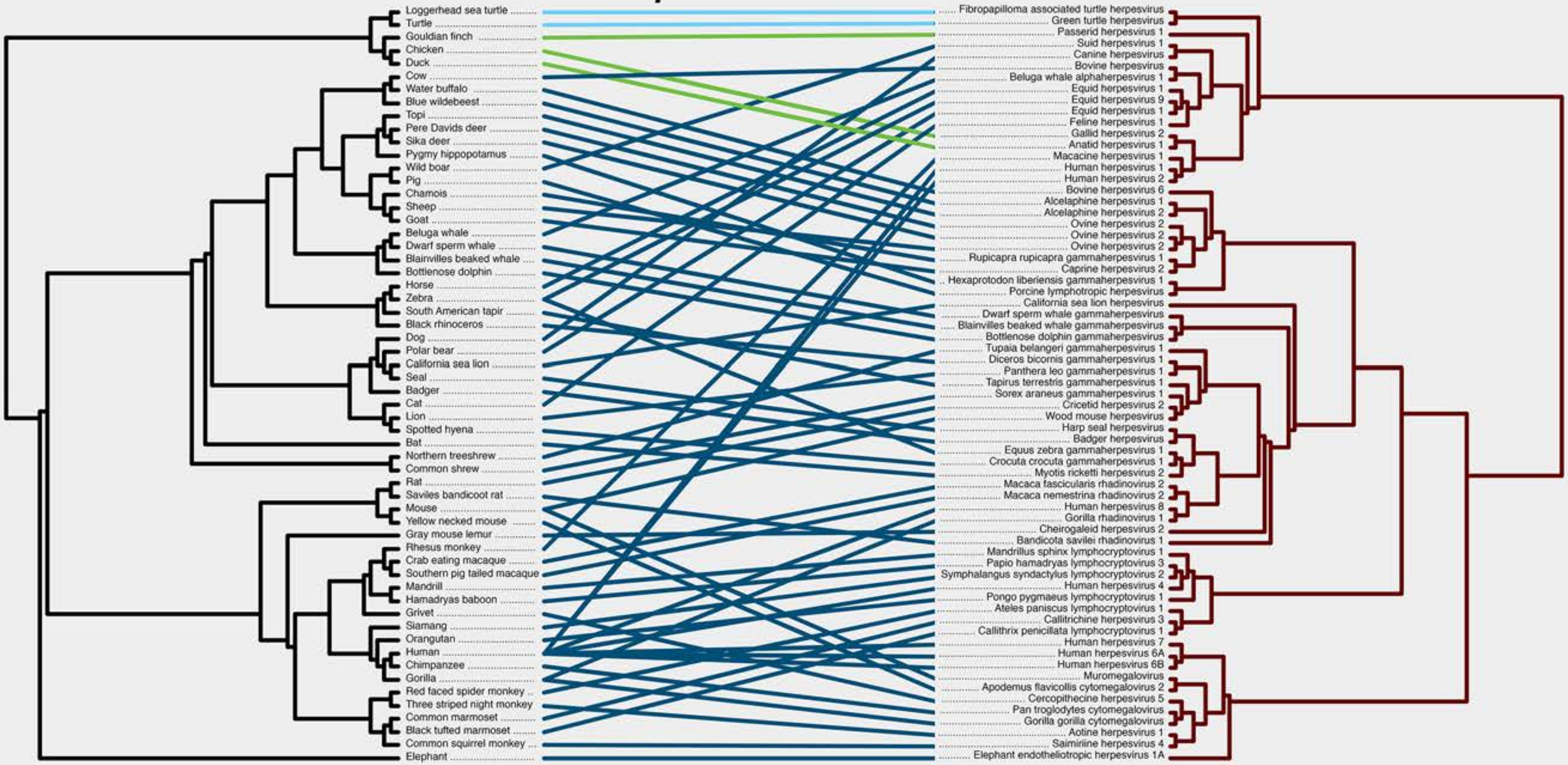

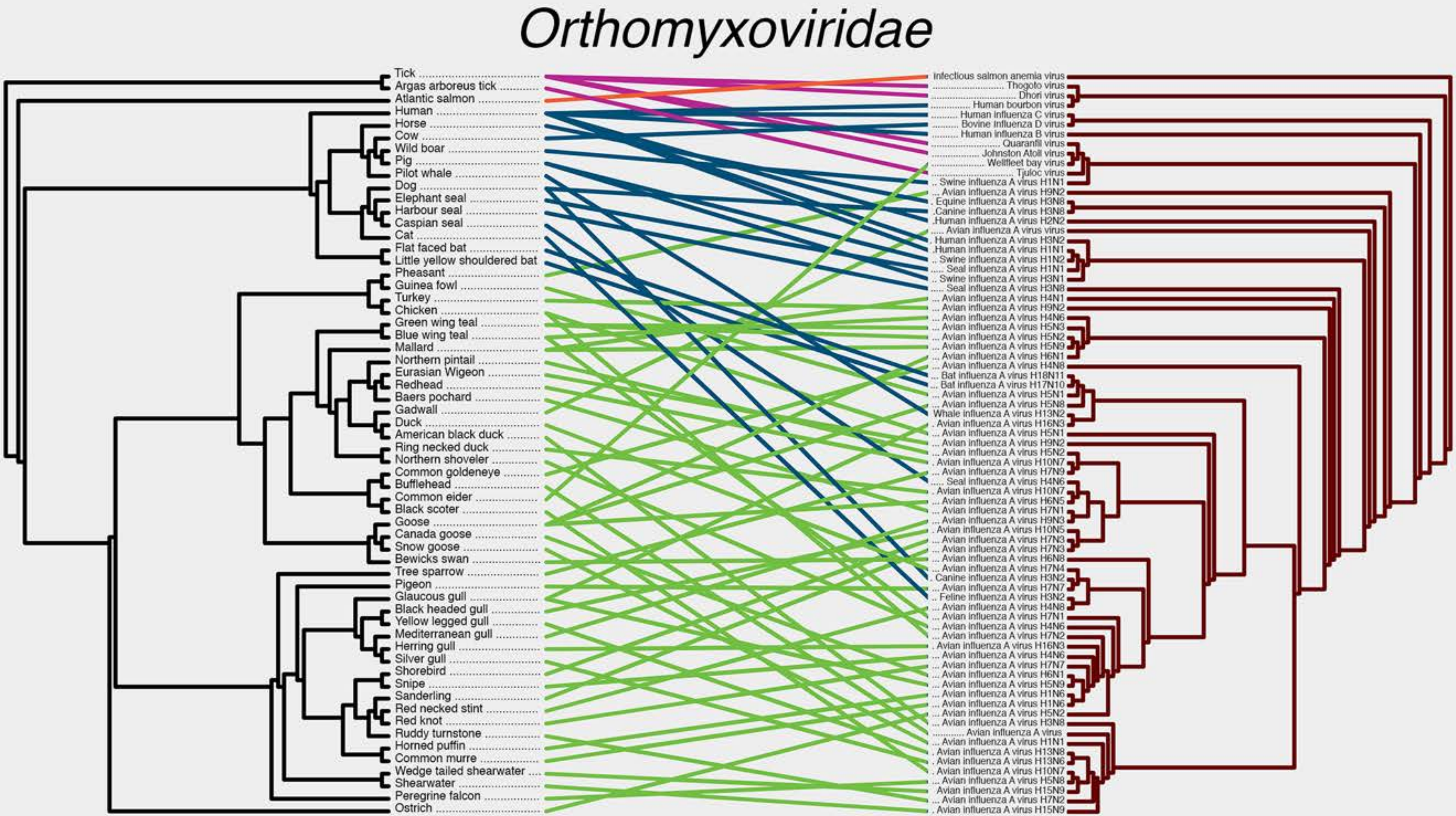

Orthomyxoviridae
Tick
Argas arboreus tick
Atlantic salmon
Human
Horse
Cow
Wild boar
Pig
Pilot whale
Dog
Elephant seal
Harbour seal
Caspian seal
Cat
Flat faced bat
Little yellow shouldered bat
Pheasant
Guinea fowl
Turkey
Chicken
Green wing teal
Blue wing teal
Mallard
Northern pintail
Eurasian Wigeon
Redhead
Baers pochard
Gadwall
Duck
American black duck
Ring necked duck
Northern shoveler
Common goldeneye
Bufflehead
Common eider
Black scoter
Goose
Canada goose
Snow goose
Bewicks swan
Tree sparrow
Pigeon
Glaucous gull
Black headed gull
Yellow legged gull
Mediterranean gull
Herring gull
Silver gull
Shorebird
Snipe
Sanderling
Red necked stint
Red knot
Ruddy turnstone
Horned puffin
Common murre
Wedge tailed shearwater
Shearwater
Peregrine falcon
Ostrich
Infectious salmon anemia virus
Thogoto virus
Dhori virus
Human bourbon virus
Human influenza C virus
Bovine influenza D virus
Human influenza B virus
Quaranfil virus
Johnston Atoll virus
Wellfleet bay virus
Tjuloc virus
Swine influenza A virus H1N1
Avian influenza A virus H9N2
Equine influenza A virus H3N8
Canine influenza A virus H3N8
Human influenza A virus H2N2
Avian influenza A virus virus
Human influenza A virus H3N2
Human influenza A virus H1N1
Swine influenza A virus H1N2
Seal influenza A virus H1N1
Swine influenza A virus H3N1
Seal influenza A virus H3N8
Avian influenza A virus H4N1
Avian influenza A virus H9N2
Avian influenza A virus H4N6
Avian influenza A virus H5N3
Avian influenza A virus H5N2
Avian influenza A virus H5N9
Avian influenza A virus H6N1
Avian influenza A virus H4N8
Bat influenza A virus H18N11
Bat influenza A virus H17N10
Avian influenza A virus H5N1
Avian influenza A virus H5N8
Whale influenza A virus H13N2
Avian influenza A virus H16N3
Avian influenza A virus H5N1
Avian influenza A virus H9N2
Avian influenza A virus H5N2
Avian influenza A virus H10N7
Avian influenza A virus H7N9
Seal influenza A virus H4N6
Avian influenza A virus H10N7
Avian influenza A virus H6N5
Avian influenza A virus H7N1
Avian influenza A virus H9N3
Avian influenza A virus H10N5
Avian influenza A virus H7N3
Avian influenza A virus H7N3
Avian influenza A virus H6N8
Avian influenza A virus H7N4
Canine influenza A virus H3N2
Avian influenza A virus H7N7
Feline influenza A virus H3N2
Avian influenza A virus H4N8
Avian influenza A virus H7N1
Avian influenza A virus H4N6
Avian influenza A virus H7N2
Avian influenza A virus H16N3
Avian influenza A virus H4N6
Avian influenza A virus H7N7
Avian influenza A virus H6N1
Avian influenza A virus H5N9
Avian influenza A virus H1N6
Avian influenza A virus H1N6
Avian influenza A virus H5N2
Avian influenza A virus H3N8
Avian influenza A virus
Avian influenza A virus H1N1
Avian influenza A virus H13N8
Avian influenza A virus H13N6
Avian influenza A virus H10N7
Avian influenza A virus H5N8
Avian influenza A virus H15N9
Avian influenza A virus H7N2
Avian influenza A virus H15N9

Papillomaviridae

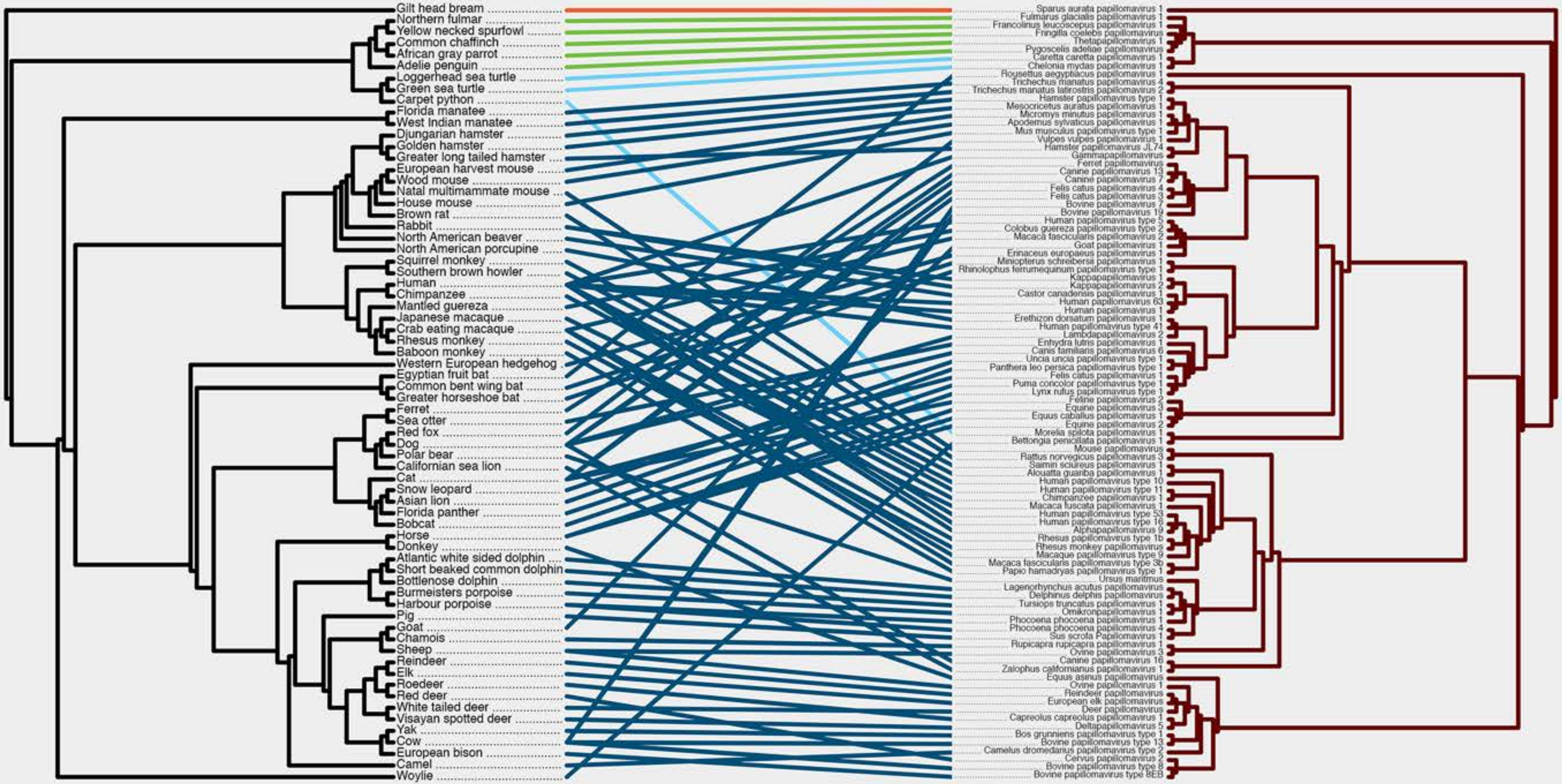

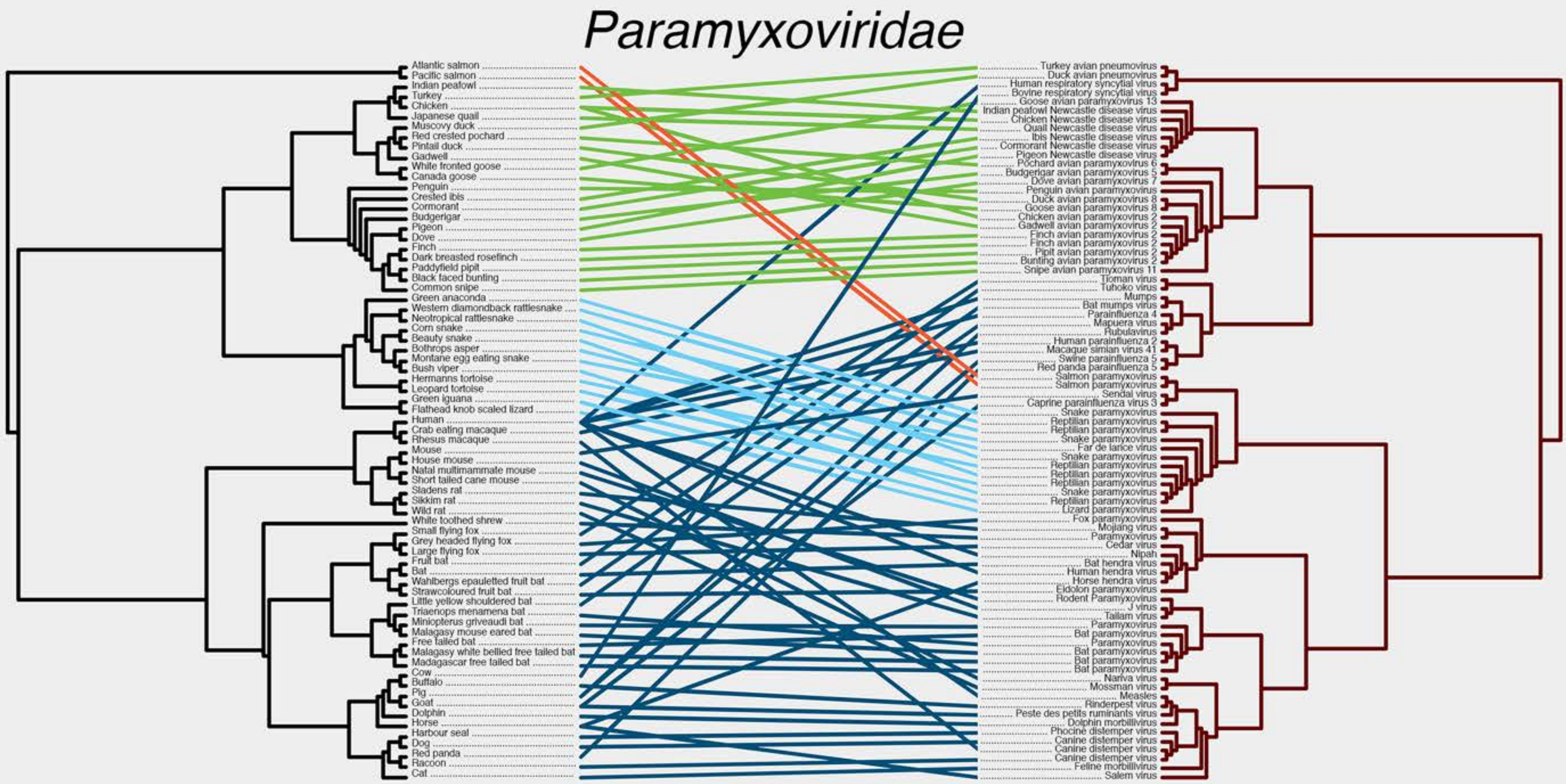

Paramyxoviridae
Atlantic salmon
Pacific salmon
Indian peafowl
Turkey
Chicken
Japanese quail
Muscovy duck
Red crested pochard
Pintail duck
Gadwell
White fronted goose
Canada goose
Penguin
Crested ibis
Cormorant
Budgerigar
Pigeon
Dove
Finch
Dark breasted rosefinch
Paddyfield pipit
Black faced bunting
Common snipe
Green anaconda
Western diamondback rattlesnake
Neotropical rattlesnake
Corn snake
Beauty snake
Bothrops asper
Montane egg eating snake
Bush viper
Hermanns tortoise
Leopard tortoise
Green iguana
Flathead knob scaled lizard
Human
Crab eating macaque
Rhesus macaque
Mouse
House mouse
Natal multimammate mouse
Short tailed cane mouse
Sladens rat
Sikkim rat
Wild rat
White toothed shrew
Small flying fox
Grey headed flying fox
Large flying fox
Fruit bat
Bat
Wahlbergs epauletted fruit bat
Strawcoloured fruit bat
Little yellow shouldered bat
Triaenops menamena bat
Miniopterus griveaudi bat
Malagasy mouse eared bat
Free tailed bat
Malagasy white bellied free tailed bat
Madagascar free tailed bat
Cow
Buffalo
Pig
Goat
Dolphin
Horse
Harbour seal
Dog
Red panda
Racoon
Cat
Turkey avian pneumovirus
Duck avian pneumovirus
Human respiratory syncytial virus
Bovine respiratory syncytial virus
Goose avian paramyxovirus 13
Indian peafowl Newcastle disease virus
Chicken Newcastle disease virus
Quail Newcastle disease virus
Ibis Newcastle disease virus
Cormorant Newcastle disease virus
Pigeon Newcastle disease virus
Pochard avian paramyxovirus 6
Budgerigar avian paramyxovirus 5
Dove avian paramyxovirus 7
Penguin avian paramyxovirus
Duck avian paramyxovirus 8
Goose avian paramyxovirus 8
Chicken avian paramyxovirus 2
Gadwell avian paramyxovirus 2
Finch avian paramyxovirus 2
Finch avian paramyxovirus 2
Pipit avian paramyxovirus 2
Bunting avian paramyxovirus 2
Snipe avian paramyxovirus 11
Tioman virus
Tuhoko virus
Mumps
Bat mumps virus
Parainfluenza 4
Mapuera virus
Rubulavirus
Human parainfluenza 2
Macaque simian virus 41
Swine parainfluenza 5
Red panda parainfluenza 5
Salmon paramyxovirus
Salmon paramyxovirus
Sendai virus
Caprine parainfluenza virus 3
Snake paramyxovirus
Reptilian paramyxovirus
Reptilian paramyxovirus
Snake paramyxovirus
Fer de lance virus
Snake paramyxovirus
Reptilian paramyxovirus
Reptilian paramyxovirus
Reptilian paramyxovirus
Snake paramyxovirus
Reptilian paramyxovirus
Lizard paramyxovirus
Fox paramyxovirus
Mojiang virus
Paramyxovirus
Cedar virus
Nipah
Bat hendra virus
Human hendra virus
Horse hendra virus
Eidolon paramyxovirus
Rodent Paramyxovirus
J virus
Tailam virus
Paramyxovirus
Bat paramyxovirus
Paramyxovirus
Bat paramyxovirus
Bat paramyxovirus
Bat paramyxovirus
Nariva virus
Mossman virus
Measles
Rinderpest virus
Peste des petits ruminants virus
Dolphin morbillivirus
Phocine distemper virus
Canine distemper virus
Canine distemper virus
Canine distemper virus
Feline morbillivirus
Salem virus

Parvoviridae

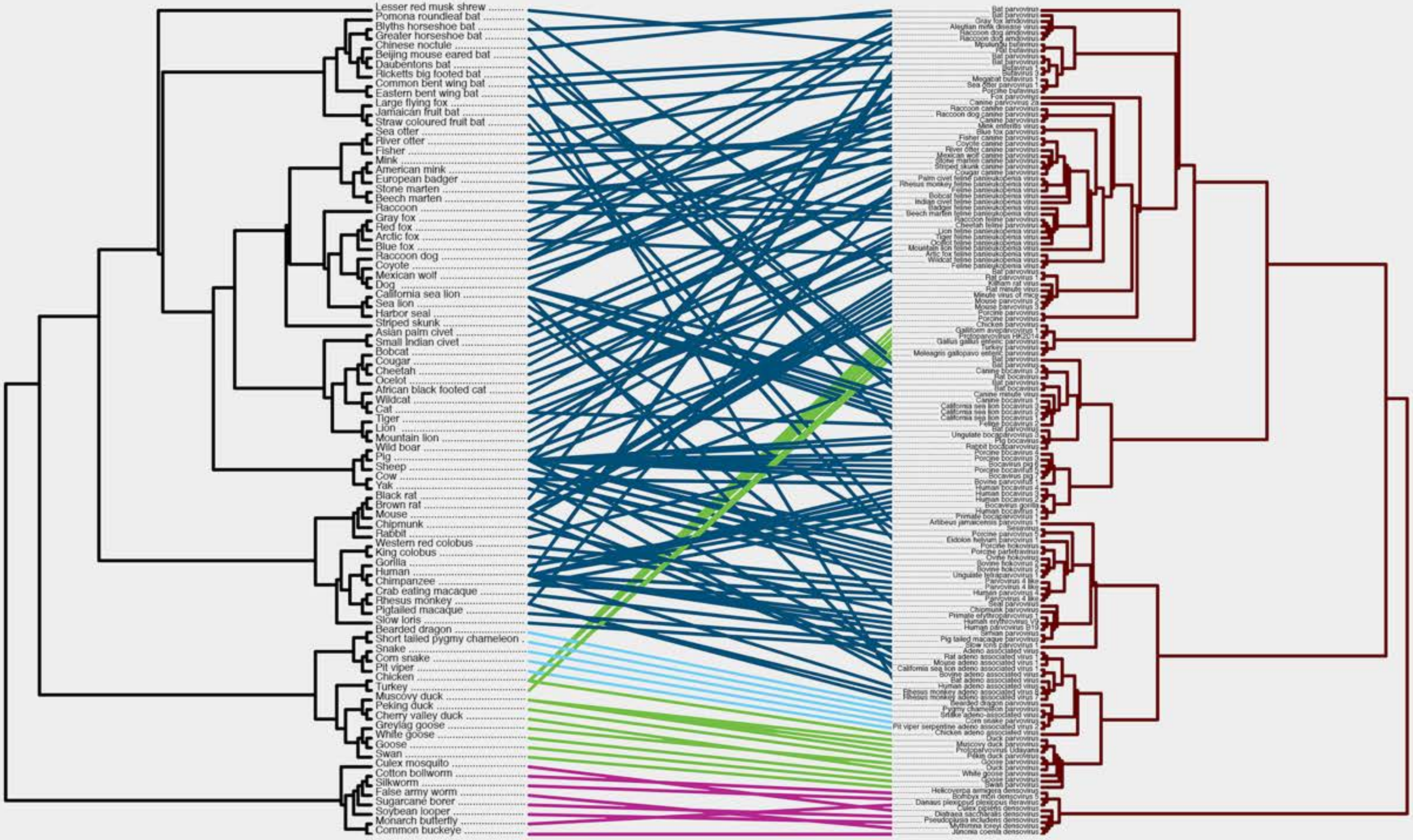

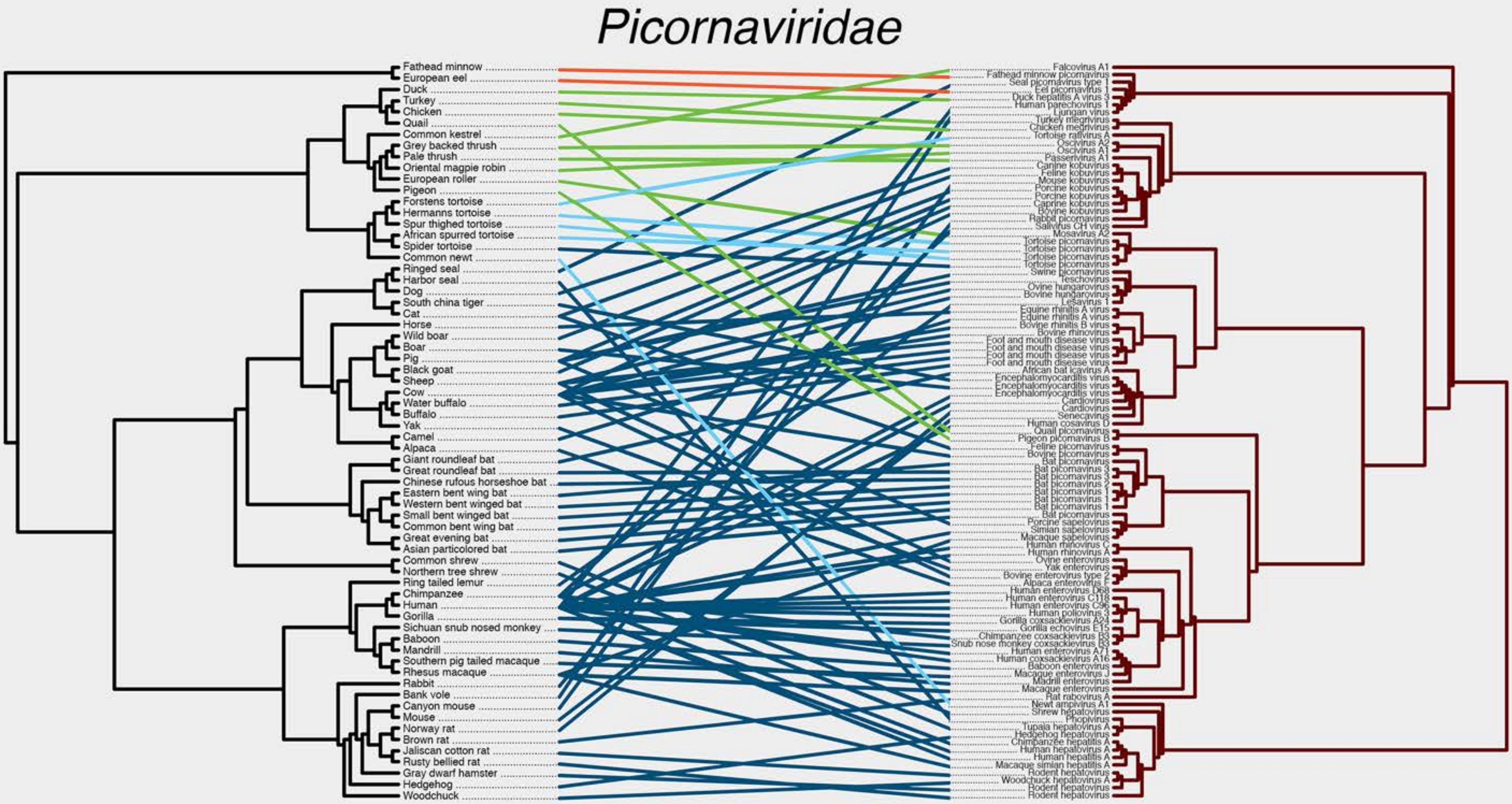

Picornaviridae
Fathead minnow
European eel
Duck
Turkey
Chicken
Quail
Common kestrel
Grey backed thrush
Pale thrush
Oriental magpie robin
European roller
Pigeon
Forstens tortoise
Hermanns tortoise
Spur thighed tortoise
African spurred tortoise
Spider tortoise
Common newt
Ringed seal
Harbor seal
Dog
South china tiger
Cat
Horse
Wild boar
Boar
Pig
Black goat
Sheep
Cow
Water buffalo
Buffalo
Yak
Camel
Alpaca
Giant roundleaf bat
Great roundleaf bat
Chinese rufous horseshoe bat
Eastern bent wing bat
Western bent winged bat
Small bent winged bat
Common bent wing bat
Great evening bat
Asian particolored bat
Common shrew
Northern tree shrew
Ring tailed lemur
Chimpanzee
Human
Gorilla
Sichuan snub nosed monkey
Baboon
Mandrill
Southern pig tailed macaque
Rhesus macaque
Rabbit
Bank vole
Canyon mouse
Mouse
Norway rat
Brown rat
Jaliscan cotton rat
Rusty bellied rat
Gray dwarf hamster
Hedgehog
Woodchuck
Falcovirus A1
Fathead minnow picornavirus
Seal picornavirus type 1
Eel picornavirus 1
Duck hepatitis A virus 3
Human parechovirus 1
Ljungan virus
Turkey megrivirus
Chicken megrivirus
Tortoise rafivirus A
Oscivirus A2
Oscivirus A1
Passerivirus A1
Canine kobuvirus
Feline kobuvirus
Mouse kobuvirus
Porcine kobuvirus
Porcine kobuvirus
Caprine kobuvirus
Bovine kobuvirus
Rabbit picornavirus
Salivirus CH virus
Mosavirus A2
Tortoise picornavirus
Tortoise picornavirus
Tortoise picornavirus
Tortoise picornavirus
Swine picornavirus
Teschovirus
Ovine hungarovirus
Bovine hungarovirus
Lesavirus 1
Equine rhinitis A virus
Equine rhinitis A virus
Bovine rhinitis B virus
Bovine rhinovirus
Foot and mouth disease virus
Foot and mouth disease virus
Foot and mouth disease virus
Foot and mouth disease virus
African bat icavirus A
Encephalomyocarditis virus
Encephalomyocarditis virus
Encephalomyocarditis virus
Cardiovirus
Cardiovirus
Senecavirus
Human cosavirus D
Quail picornavirus
Pigeon picornavirus B
Feline picornavirus
Bovine picornavirus
Bat picornavirus
Bat picornavirus 3
Bat picornavirus 3
Bat picornavirus 2
Bat picornavirus 1
Bat picornavirus 1
Bat picornavirus 1
Bat picornavirus
Porcine sapelovirus
Simian sapelovirus
Macaque sapelovirus
Human rhinovirus C
Human rhinovirus A
Ovine enterovirus
Yak enterovirus
Bovine enterovirus type 2
Alpaca enterovirus F
Human enterovirus D68
Human enterovirus C118
Human enterovirus C96
Human poliovirus 3
Gorilla coxsackievirus A24
Gorilla echovirus E15
Chimpanzee coxsackievirus B3
Snub nose monkey coxsackievirus B3
Human enterovirus A71
Human coxsackievirus A16
Baboon enterovirus
Macaque enterovirus J
Madrill enterovirus
Macaque enterovirus
Rat rabovirus A
Newt ampivirus A1
Shrew hepatovirus
Phopivirus
Tupaia hepatovirus A
Hedgehog hepatovirus
Chimpanzee hepatitis A
Human hepatovirus A
Human hepatitis A
Macaque simian hepatitis A
Rodent hepatovirus
Woodchuck hepatovirus A
Rodent hepatovirus
Rodent hepatovirus

Polyomaviridae

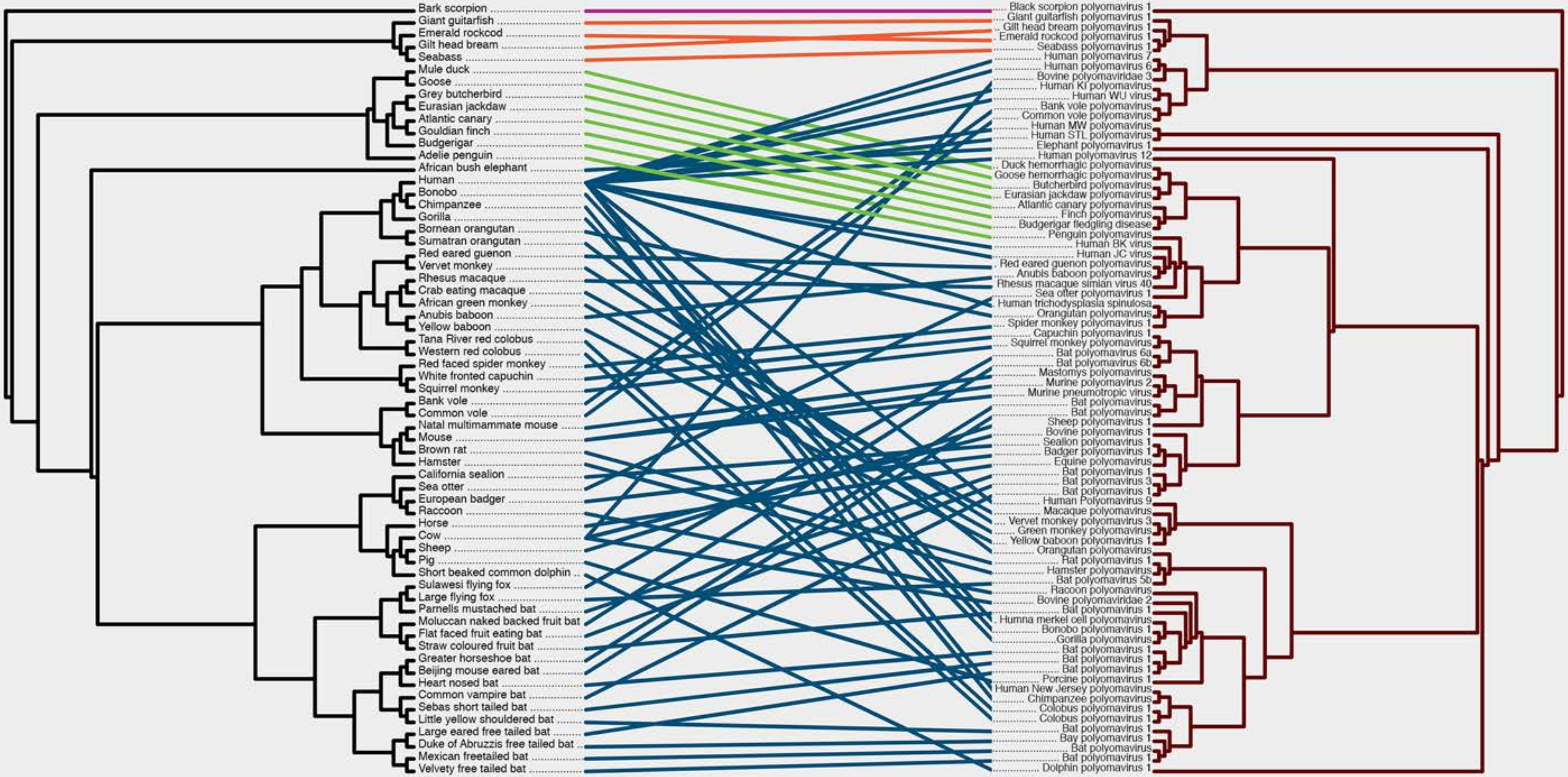

Potyviridae

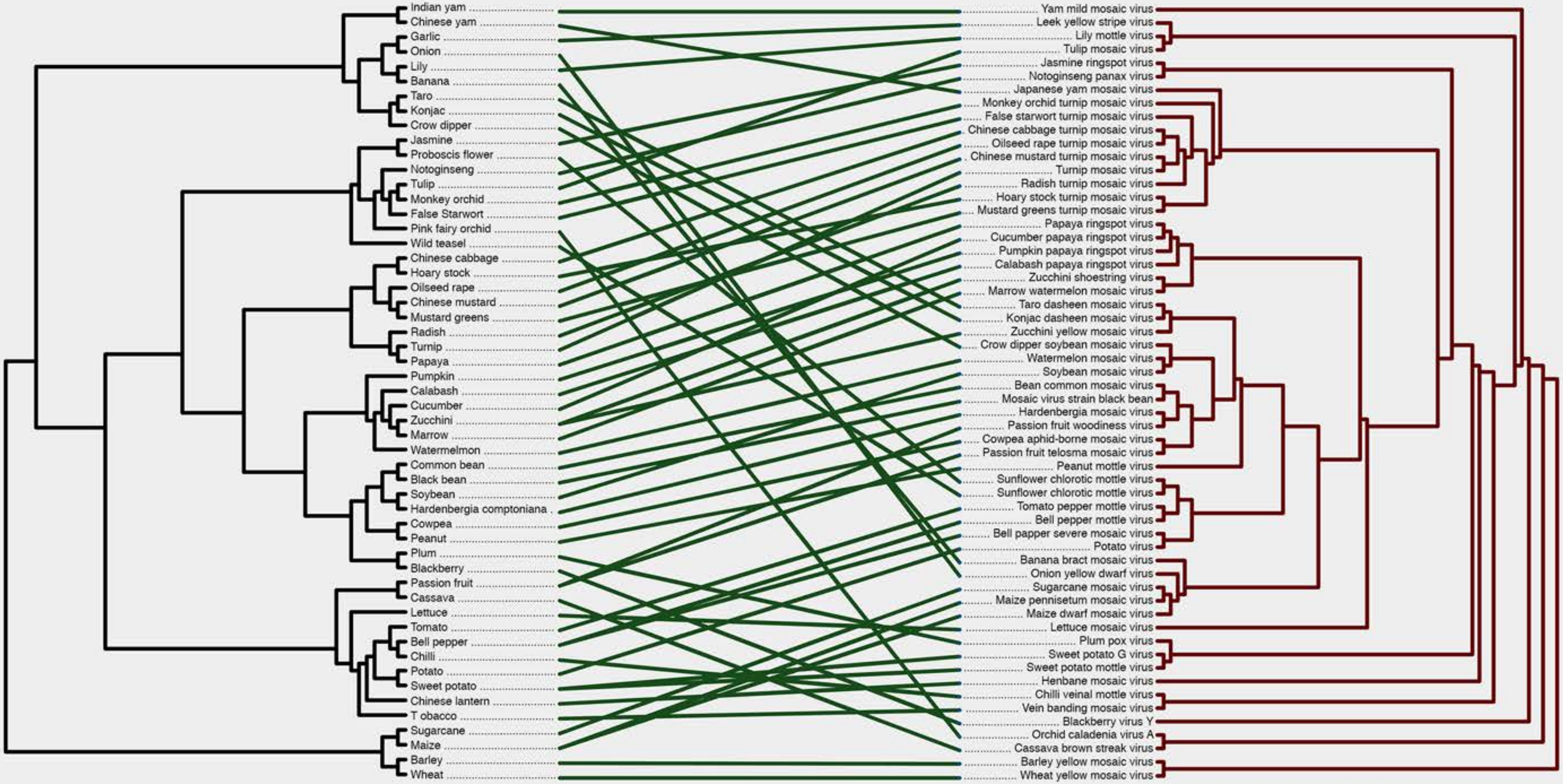

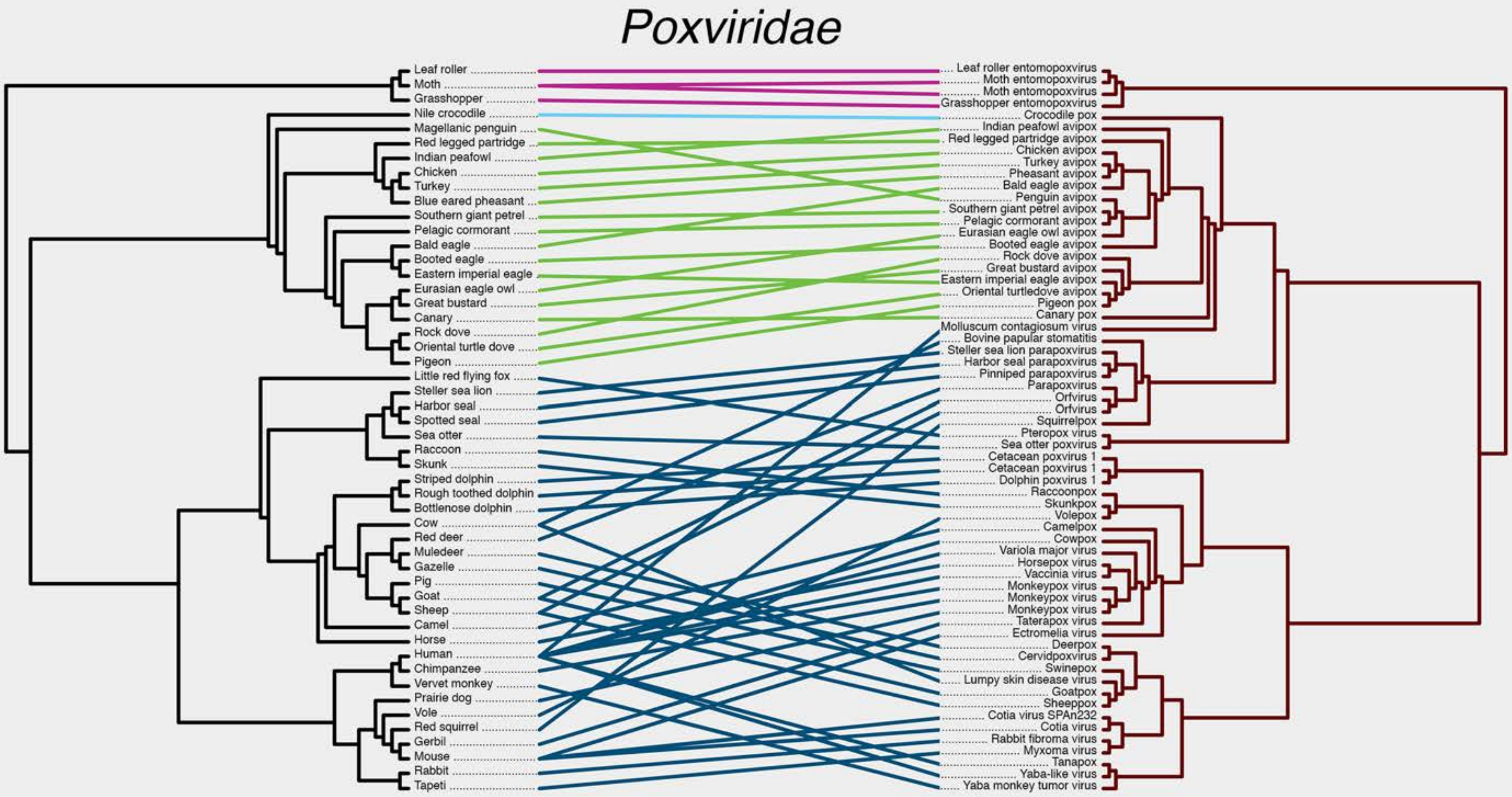
Poxviridae
Leaf roller
Moth
Grasshopper
Nile crocodile
Magellanic penguin
Red legged partridge
Indian peafowl
Chicken
Turkey
Blue eared pheasant
Southern giant petrel
Pelagic cormorant
Bald eagle
Booted eagle
Eastern imperial eagle
Eurasian eagle owl
Great bustard
Canary
Rock dove
Oriental turtle dove
Pigeon
Little red flying fox
Steller sea lion
Harbor seal
Spotted seal
Sea otter
Raccoon
Skunk
Striped dolphin
Rough toothed dolphin
Bottlenose dolphin
Cow
Red deer
Muledeer
Gazelle
Pig
Goat
Sheep
Camel
Horse
Human
Chimpanzee
Vervet monkey
Prairie dog
Vole
Red squirrel
Gerbil
Mouse
Rabbit
Tapeti
Leaf roller entomopoxvirus
Moth entomopoxvirus
Moth entomopoxvirus
Grasshopper entomopoxvirus
Crocodile pox
Indian peafowl avipox
Red legged partridge avipox
Chicken avipox
Turkey avipox
Pheasant avipox
Bald eagle avipox
Penguin avipox
Southern giant petrel avipox
Pelagic cormorant avipox
Eurasian eagle owl avipox
Booted eagle avipox
Rock dove avipox
Great bustard avipox
Eastern imperial eagle avipox
Oriental turtledove avipox
Pigeon pox
Canary pox
Molluscum contagiosum virus
Bovine papular stomatitis
Steller sea lion parapoxvirus
Harbor seal parapoxvirus
Pinniped parapoxvirus
Parapoxvirus
Orfvirus
Orfvirus
Squirrelpox
Pteropox virus
Sea otter poxvirus
Cetacean poxvirus 1
Cetacean poxvirus 1
Dolphin poxvirus 1
Raccoonpox
Skunkpox
Volepox
Camelpox
Cowpox
Variola major virus
Horsepox virus
Vaccinia virus
Monkeypox virus
Monkeypox virus
Monkeypox virus
Taterapox virus
Ectromelia virus
Deerpox
Cervidpoxvirus
Swinepox
Lumpy skin disease virus
Goatpox
Sheeppox
Cotia virus SPAn232
Cotia virus
Rabbit fibroma virus
Myxoma virus
Tanapox
Yaba-like virus
Yaba monkey tumor virus

Reoviridae

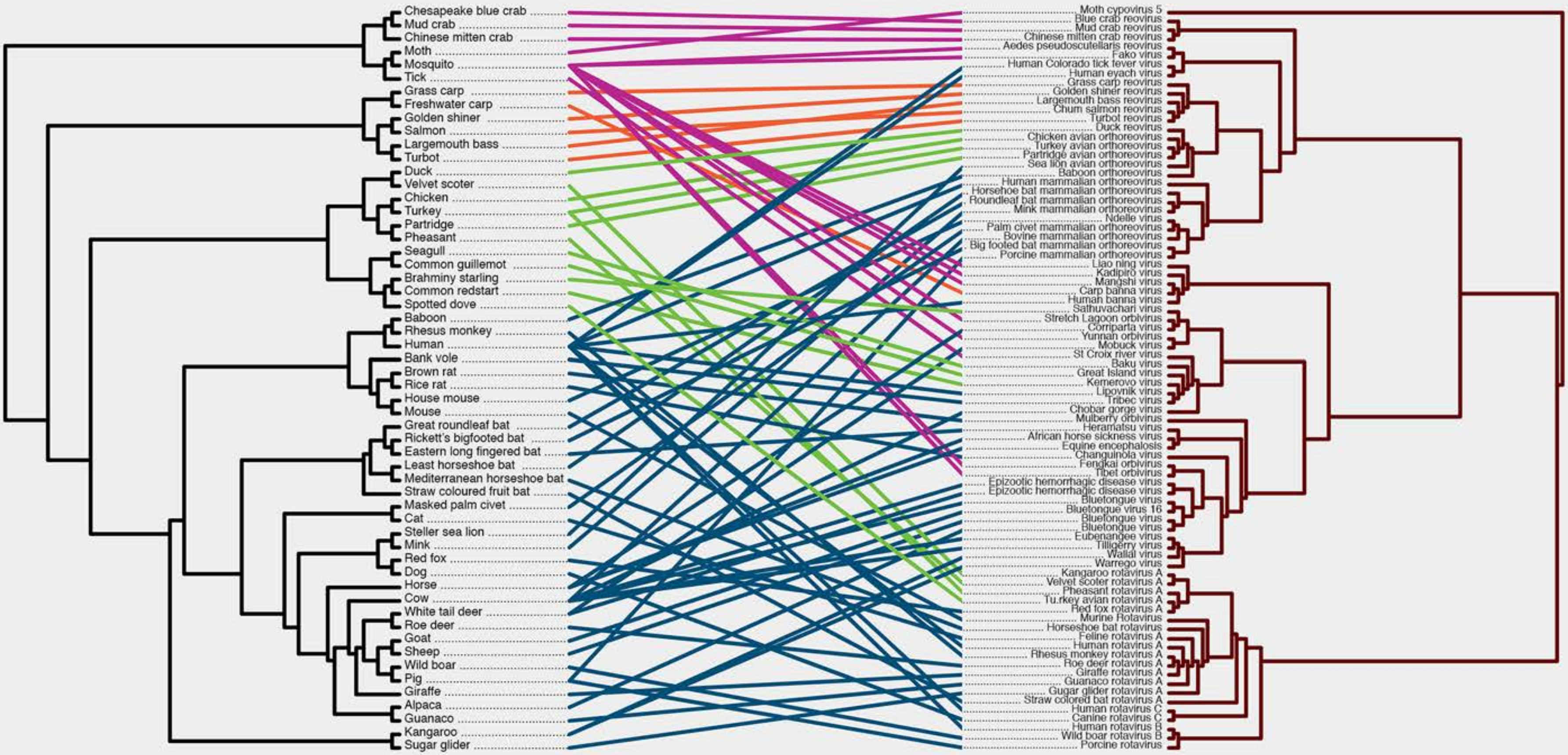

Chesapeake blue crab
Mud crab
Chinese mitten crab
Moth
Mosquito
Tick
Grass carp
Freshwater carp
Golden shiner
Salmon
Largemouth bass
Turbot
Duck
Velvet scoter
Chicken
Turkey
Partridge
Pheasant
Seagull
Common guillemot
Brahminy starling
Common redstart
Spotted dove
Baboon
Rhesus monkey
Human
Bank vole
Brown rat
Rice rat
House mouse
Mouse
Great roundleaf bat
Rickett's bigfooted bat
Eastern long fingered bat
Least horseshoe bat
Mediterranean horseshoe bat
Straw coloured fruit bat
Masked palm civet
Cat
Steller sea lion
Mink
Red fox
Dog
Horse
Cow
White tail deer
Roe deer
Goat
Sheep
Wild boar
Pig
Giraffe
Alpaca
Guanaco
Kangaroo
Sugar glider
Moth cypovirus 5
Blue crab reovirus
Mud crab reovirus
Chinese mitten crab reovirus
Aedes pseudoscutellaris reovirus
Fako virus
Human Colorado tick fever virus
Human eyach virus
Grass carp reovirus
Golden shiner reovirus
Largemouth bass reovirus
Chum salmon reovirus
Turbot reovirus
Duck reovirus
Chicken avian orthoreovirus
Turkey avian orthoreovirus
Partridge avian orthoreovirus
Sea lion avian orthoreovirus
Baboon orthoreovirus
Human mammalian orthoreovirus
Horsehoe bat mammalian orthoreovirus
Roundleaf bat mammalian orthoreovirus
Mink mammalian orthoreovirus
Ndelle virus
Palm civet mammalian orthoreovirus
Bovine mammalian orthoreovirus
Big footed bat mammalian orthoreovirus
Porcine mammalian orthoreovirus
Liao ning virus
Kadipiro virus
Mangshi virus
Carp banna virus
Human banna virus
Sathuvachari virus
Stretch Lagoon orbivirus
Corriparta virus
Yunnan orbivirus
Mobuck virus
St Croix river virus
Baku virus
Great Island virus
Kemerovo virus
Lipovnik virus
Tribec virus
Chobar gorge virus
Mulberry orbivirus
Heramatsu virus
African horse sickness virus
Equine encephalosis
Changuinola virus
Fengkai orbivirus
Tibet orbivirus
Epizootic hemorrhagic disease virus
Epizootic hemorrhagic disease virus
Bluetongue virus
Bluetongue virus 16
Bluetongue virus
Bluetongue virus
Eubenangee virus
Tilligerry virus
Wallal virus
Warrego virus
Kangaroo rotavirus A
Velvet scoter rotavirus A
Pheasant rotavirus A
Tu.rkey avian rotavirus A
Red fox rotavirus A
Murine Rotavirus
Horseshoe bat rotavirus
Feline rotavirus A
Human rotavirus A
Rhesus monkey rotavirus A
Roe deer rotavirus A
Giraffe rotavirus A
Guanaco rotavirus A
Gugar glider rotavirus A
Straw colored bat rotavirus A
Human rotavirus C
Canine rotavirus C
Human rotavirus B
Wild boar rotavirus B
Porcine rotavirus

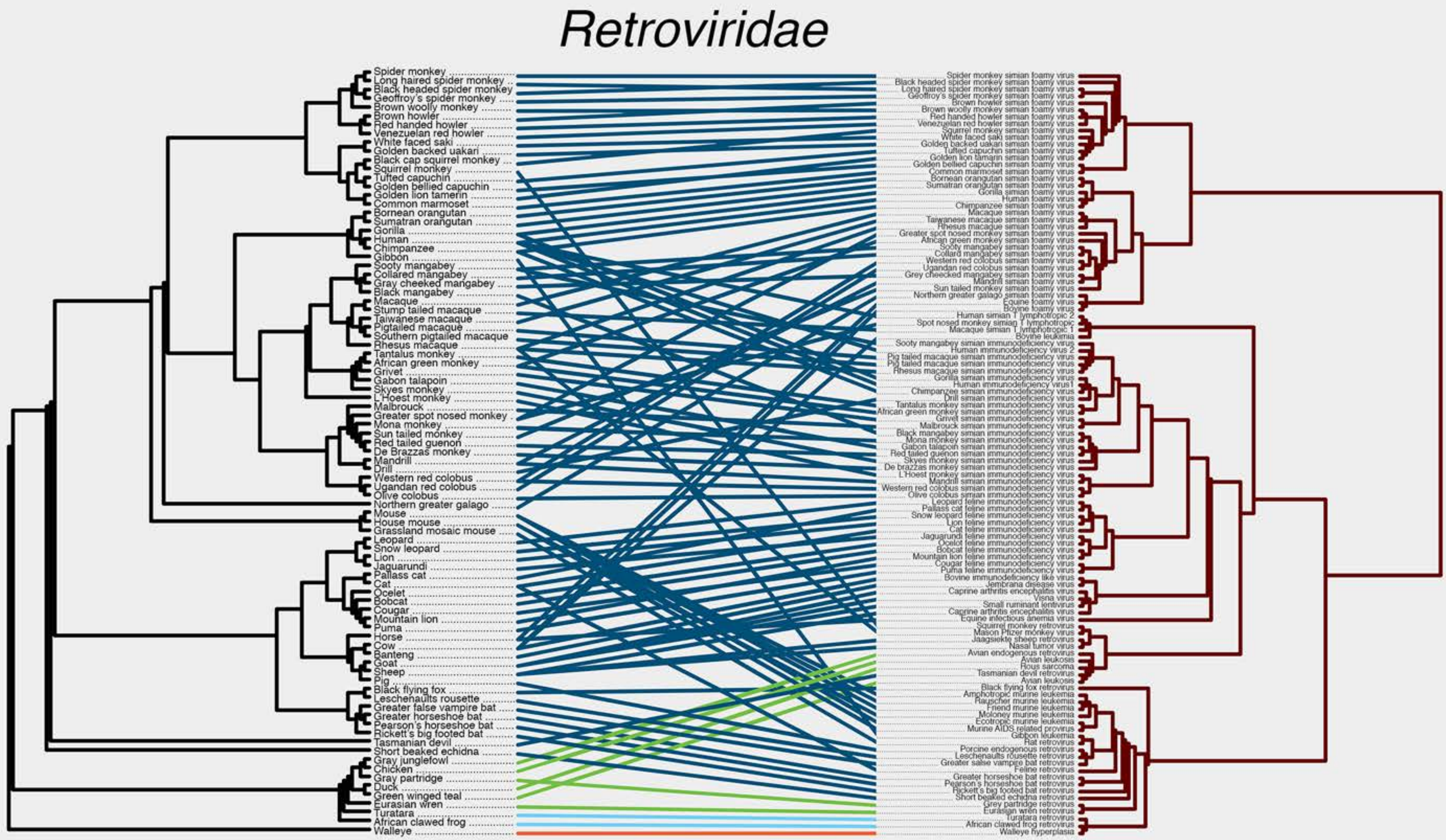
Retroviridae
Spider monkey
Long haired spider monkey
Black headed spider monkey
Geoffroy's spider monkey
Brown woolly monkey
Brown howler
Red handed howler
Venezuelan red howler
White faced saki
Golden backed uakari
Black cap squirrel monkey
Squirrel monkey
Tufted capuchin
Golden bellied capuchin
Golden lion tamerin
Common marmoset
Bornean orangutan
Sumatran orangutan
Gorilla
Human
Chimpanzee
Gibbon
Sooty mangabey
Collared mangabey
Gray cheeked mangabey
Black mangabey
Macaque
Stump tailed macaque
Taiwanese macaque
Pigtailed macaque
Southern pigtailed macaque
Rhesus macaque
Tantalus monkey
African green monkey
Grivet
Gabon talapoin
Skyes monkey
L'Hoest monkey
Malbrouck
Greater spot nosed monkey
Mona monkey
Sun tailed monkey
Red tailed guenon
De Brazzas monkey
Mandrill
Drill
Western red colobus
Ugandan red colobus
Olive colobus
Northern greater galago
Mouse
House mouse
Grassland mosaic mouse
Leopard
Snow leopard
Lion
Jaguarundi
Pallass cat
Cat
Ocelet
Bobcat
Cougar
Mountain lion
Puma
Horse
Cow
Banteng
Goat
Sheep
Pig
Black flying fox
Leschenaults rousette
Greater false vampire bat
Greater horseshoe bat
Pearson's horseshoe bat
Rickett's big footed bat
Tasmanian devil
Short beaked echidna
Gray junglefowl
Chicken
Gray partridge
Duck
Green winged teal
Eurasian wren
Turatara
African clawed frog
Walleye
Spider monkey simian foamy virus
Black headed spider monkey simian foamy virus
Long haired spider monkey simian foamy virus
Geoffroy's spider monkey simian foamy virus
Brown howler simian foamy virus
Brown woolly monkey simian foamy virus
Red handed howler simian foamy virus
Venezuelan red howler simian foamy virus
Squirrel monkey simian foamy virus
White faced saki simian foamy virus
Golden backed uakari simian foamy virus
Tufted capuchin simian foamy virus
Golden lion tamarin simian foamy virus
Golden bellied capuchin simian foamy virus
Common marmoset simian foamy virus
Bornean orangutan simian foamy virus
Sumatran orangutan simian foamy virus
Gorilla simian foamy virus
Human simian foamy virus
Chimpanzee simian foamy virus
Macaque simian foamy virus
Taiwanese macaque simian foamy virus
Rhesus macaque simian foamy virus
Greater spot nosed monkey simian foamy virus
African green monkey simian foamy virus
Sooty mangabey simian foamy virus
Collard mangabey simian foamy virus
Western red colobus simian foamy virus
Ugandan red colobus simian foamy virus
Grey cheecked mangabey simian foamy virus
Mandrill simian foamy virus
Sun tailed monkey simian foamy virus
Northern greater galago simian foamy virus
Equine foamy virus
Bovine foamy virus
Human simian T lymphotropic 2
Spot nosed monkey simian T lymphotropic
Macaque simian T lymphotropic 1
Bovine leukimia
Sooty mangabey simian immunodeficiency virus
Human immunodeficiency virus 2
Pig tailed macaque simian immunodeficiency virus
Pig tailed macaque simian immunodeficiency virus
Rhesus macaque simian immunodeficiency virus
Gorilla simian immunodeficiency virus
Human immunodeficiency virus1
Chimpanzee simian immunodeficiency virus
Drill simian immunodeficiency virus
Tantalus monkey simian immunodeficiency virus
African green monkey simian immunodeficiency virus
Grivet simian immunodeficiency virus
Malbrouck simian immunodeficiency virus
Black mangabey simian immunodeficiency virus
Mona monkey simian immunodeficiency virus
Gabon talapoin simian immunodeficiency virus
Red tailed guenon simian immunodeficiency virus
Skyes monkey simian immunodeficiency virus
De brazzas monkey simian immunodeficiency virus
L'Hoest monkey simian immunodeficiency virus
Mandrill simian immunodeficiency virus
Western red colobus simian immunodeficiency virus
Olive colobus simian immunodeficiency virus
Leopard feline immunodeficiency virus
Pallass cat feline immunodeficiency virus
Snow leopard feline immunodeficiency virus
Lion feline immunodeficiency virus
Cat feline immunodeficiency virus
Jaguarundi feline immunodeficiency virus
Ocelot feline immunodeficiency virus
Bobcat feline immunodeficiency virus
Mountain lion feline immunodeficiency virus
Cougar feline immunodeficiency virus
Puma feline immunodeficiency virus
Bovine immunodeficiency like virus
Jembrana disease virus
Caprine arthritis encephalitis virus
Visna virus
Small ruminant lentivirus
Caprine arthritis encephalitis virus
Equine infectious anemia virus
Squirrel monkey retrovirus
Mason Pfizer monkey virus
Jaagsiekte sheep retrovirus
Nasal tumor virus
Avian endogenous retrovirus
Avian leukosis
Rous sarcoma
Tasmanian devil retrovirus
Avian leukosis
Black flying fox retrovirus
Amphotropic murine leukemia
Rauscher murine leukemia
Friend murine leukemia
Moloney murine leukemia
Ecotropic murine leukemia
Murine AIDS related provirus
Gibbon leukemia
Rat retrovirus
Porcine endogenous retrovirus
Leschenaults rousette retrovirus
Greater salse vampire bat retrovirus
Feline retrovirus
Greater horseshoe bat retrovirus
Pearson's horseshoe bat retrovirus
Rickett's big footed bat retrovirus
Short beaked echidna retrovirus
Grey partridge retrovirus
Eurasian wren retrovirus
Turatara retrovirus
African clawed frog retrovirus
Walleye hyperplasia

Rhabdoviridae

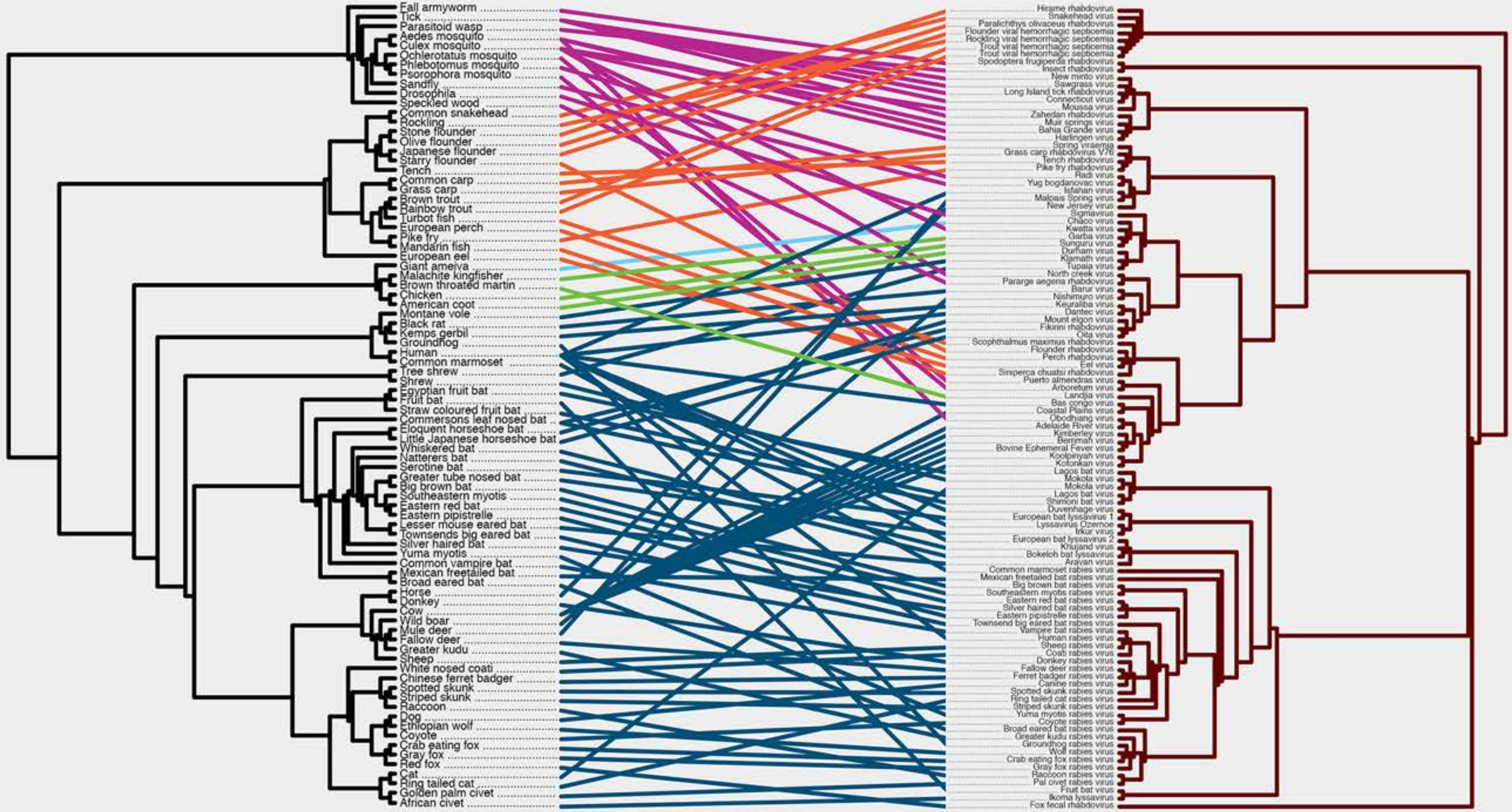

Togaviridae

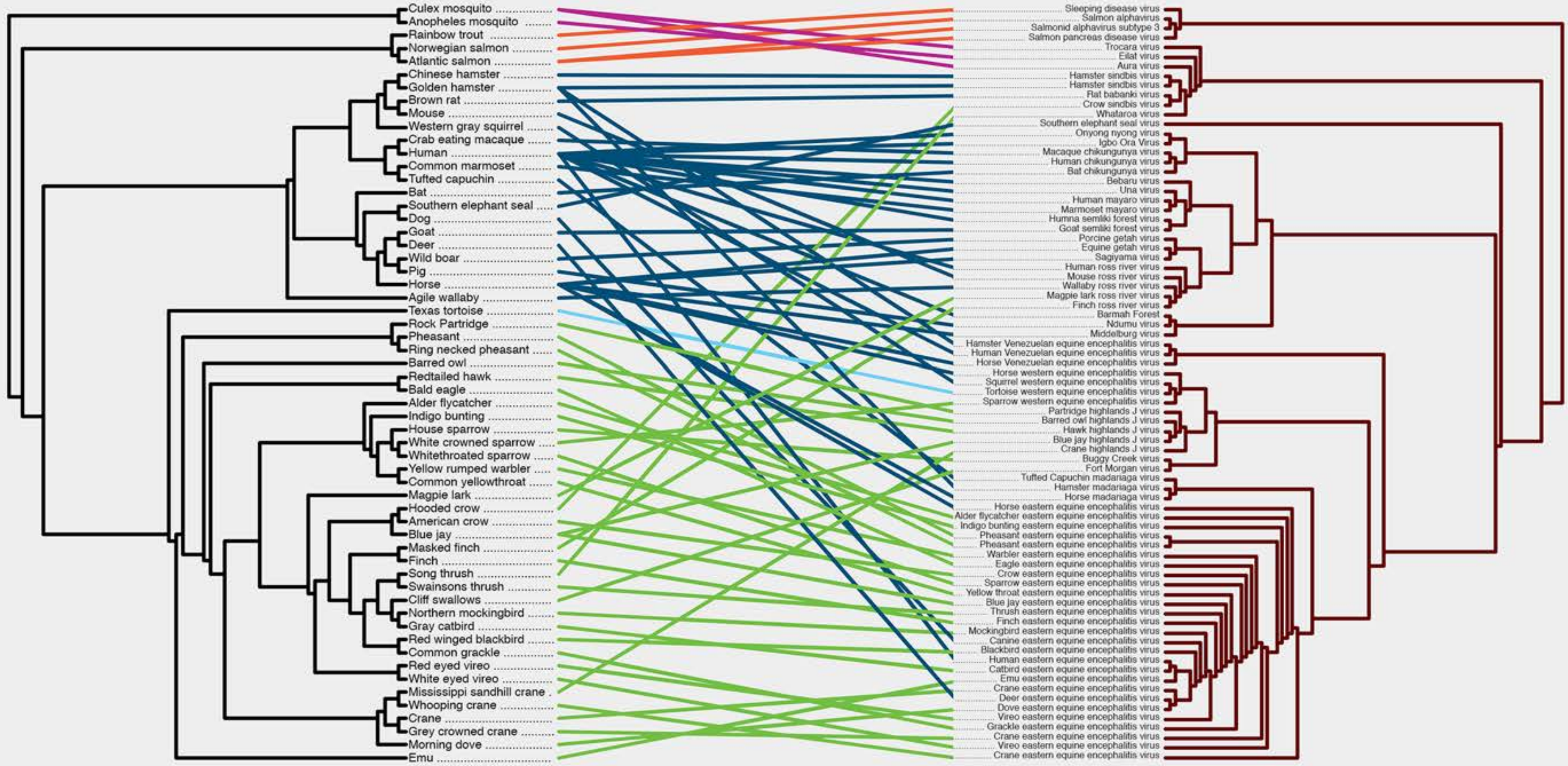

Supplement: S1 Fig — Common names for host species are used and virus names identify the host where appropriate. (PDF) [file ppat.1006215.s001.pdf]
