# Supplementary figures and images for: Comparative analysis estimates the relative frequencies of co-divergence and cross-species transmission within viral families
Source: PLoS Pathog. 2017 Feb 8;13(2):e1006215. doi: 10.1371/journal.ppat.1006215 (PMC5319820; doi:10.1371/journal.ppat.1006215)

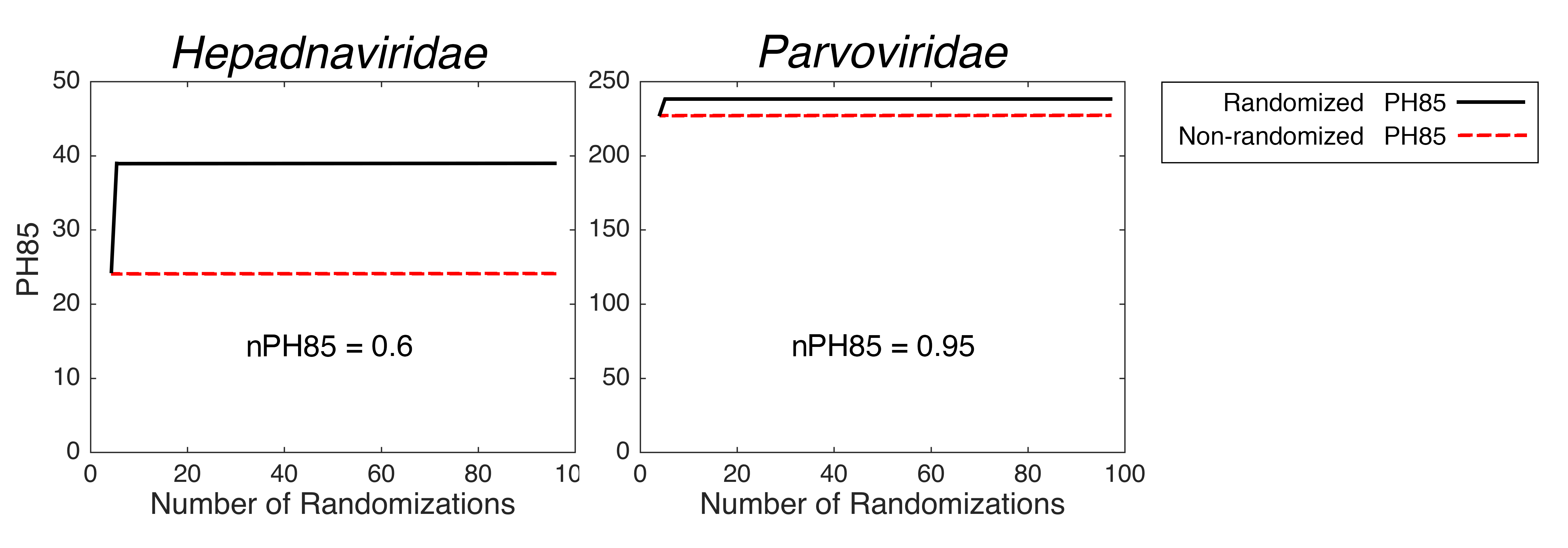

Supplement: S2 Fig — The red, dashed line illustrates the PH85 distance of the non-randomized data, while the black, solid line is the PH85 distance after randomizing the data after n randomizations. (TIF) [file ppat.1006215.s002.tif]
