# Supplementary material for: Comparative analysis estimates the relative frequencies of co-divergence and cross-species transmission within viral families
Source: PLoS Pathog. 2017 Feb 8;13(2):e1006215. doi: 10.1371/journal.ppat.1006215 (PMC5319820; doi:10.1371/journal.ppat.1006215)
Supplement: S1 Table — (DOCX) [file ppat.1006215.s003.docx]

**Supplementary Table 1.** GenBank accession numbers for the virus and host gene sequence data utilized here.

| **Virus Family** | **GenBank Accession Number** | **Virus Species** | **Host Species** |
| --- | --- | --- | --- |
| *Adenoviridae* | U77082 | Canine adenovirus 2 | Dog |
|  | U40839 | Ovine adenovirus 7 | Sheep |
|  | NC_030860 | Bat mastadenovirus WIV12 | Common bent wing bat |
|  | NC_030792 | Equine adenovirus 1 | Horse |
|  | NC_030116 | unidentified adenovirus | Penguin |
|  | NC_029899 | Bat mastadenovirus WIV10 | Chinese rufous horseshoe bat |
|  | NC_028107 | Simian adenovirus 19 | Yellow baboon |
|  | NC_028105 | Simian adenovirus 16 | Vervet monkey |
|  | NC_027708 | Skunk adenovirus PB1 | American hog nosed skunk |
|  | NC_027705 | Equine adenovirus 2 | Horse |
|  | NC_025962 | Psittacine adenovirus 3 | Mealy amazon parrot |
|  | NC_024684 | Lizard adenovirus 2 | Mexican beaded lizard |
|  | NC_024486 | Duck adenovirus 2 | Muscovy duck |
|  | NC_024474 | Pigeon adenovirus 1 | Pigeon |
|  | NC_021168 | Simian adenovirus C | Baboon |
|  | NC_017825 | Chimpanzee adenovirus Y25 | Chimpanzee |
|  | NC_016437 | Skua adenovirus 1 | South polar skua |
|  | NC_015932 | Bat adenovirus 2 | Common pipistrelle |
|  | NC_015455 | Raptor adenovirus 1 | Harris hawk |
|  | NC_014899 | Murine adenovirus 2 | House mouse |
|  | NC_012584 | Murine adenovirus 3 | Striped field mouse |
|  | NC_011202 | Human mastadenovirus B | Human |
|  | NC_009989 | Snake adenovirus 1 | Corn snake |
|  | NC_002702 | Porcine adenovirus 5 | Pig |
|  | NC_002685 | Bovine adenovirus D | Cow |
|  | NC_002513 | Bovine adenovirus 2 | Cow |
|  | NC_002501 | Frog adenovirus | Frog |
|  | NC_001958 | Turkey adenovirus 3 | Turkey |
|  | NC_001454 | Human adenovirus F | Human |
|  | KX077988 | Fowl aviadenovirus E | Chicken |
|  | KU746335 | Fowl aviadenovirus D | Chicken |
|  | KU245540 | Fowl aviadenovirus C | Chicken |
|  | KT013209 | Cynomolgus adenovirus 1 | Crab eating macaque |
|  | KP329562 | Simian adenovirus 11 | Rhesus macaque |
|  | KJ563221 | California sea lion adenovirus 1 | California sea lion |
|  | KJ452172 | Duck adenovirus 1 | Pekin duck |
|  | KF951595 | Human mastadenovirus C | Human |
|  | KC309438 | Gull adenovirus | European herring gull |
|  | JX625134 | Human adenovirus 7 | Human |
|  | JN226747 | Human adenovirus 13 | Human |
|  | JN226746 | Human adenovirus 10 | Human |
|  | JF510462 | Goose adenovirus 4 | Goose |
|  | HQ292614 | Gorilla gorilla adenovirus B7 | Gorilla |
|  | HQ241820 | Simian adenovirus 50 | Crab eating macaque |
|  | GU226970 | Bat adenovirus TJM | Ricketts big footed bat |
|  | FJ849795 | Great tit adenovirus 1 | Great tit |
|  | AY849321 | Avirulent turkey hemorrhagic enteritis virus | Pheasant |
|  | AY458656 | Human adenovirus E4 | Human |
|  | AP012302 | Human adenovirus 67 | Human |
|  | AF258784 | Tree shrew adenovirus | Tree shrew |
|  | AC_000191 | Bovine mastadenovirus A | Cow |
|  | AC_000017 | Human adenovirus 1 | Human |
|  | AC_000016 | Turkey siadenovirus A | Turkey |
|  | AC_000006 | Human adenovirus D | Human |
|  | AC_000003 | Canine adenovirus 1 | Dog |
| *Bunyaviridae* | AB620102 | Montano virus | Orizaba deer mouse |
|  | AB712372 | Hokkaido virus | Grey red backed vole |
|  | AB968525 | Akabane virus | Cow |
|  | AF005729 | Laguna negra virus | Small vesper mouse |
|  | AF288297 | Seoul virus | Human |
|  | AJ410618 | Saaremaa virus | Striped field mouse |
|  | DQ375434 | Rift Valley fever virus | Cow |
|  | EF397003 | Choclo virus | Fulvous pygmy rice rat |
|  | EF543525 | Cao bang virus | Chinese mole shrew |
|  | EF646763 | Prospect Hill virus | Meadow vole |
|  | EU257628 | Kupe virus | Sheep |
|  | EU697951 | Nairobi sheep disease virus | Sheep |
|  | EU929078 | Asama virus | Japanses shrew mole |
|  | FJ593497 | Oxbow virus | American shrew mole |
|  | FJ593501 | Jemez springs virus | Montane shrew |
|  | FJ809772 | Rio Mamore virus | Small eared pygmy rice rat |
|  | FJ858378 | Catacamas virus | Coues rice rat |
|  | GQ200821 | Cano delgadito virus | Alstons cotton rat |
|  | GQ244526 | Bayou virus | Marsh rice rat |
|  | GU566021 | Qian hu shan virus | Greater stripe backed shrew |
|  | GU997097 | Black creek canal virus | Hispid cotton rat |
|  | HM015222 | Rockport virus | Eastern mole |
|  | HPSVRPL | Sin nombre virus | Human |
|  | HQ541738 | Gouleako virus | Pig |
|  | HQ834697 | Jeju virus | Asian lesser white toothed shrew |
|  | JF784386 | Rift Valley fever virus | Sheep |
|  | JQ026206 | Dobrava Belgrade virus | Human |
|  | JQ082302 | Sangassou virus | African wood mouse |
|  | JQ083393 | Hantaan virus | Human |
|  | JX028271 | Muju virus | Royal vole |
|  | JX193697 | Uluguru virus | Geata mouse shrew |
|  | JX193700 | Kilimanjaro virus | Kilimanjaro mouse shrew |
|  | JX846600 | Batai virus | Anopheles mosquito |
|  | JX912953 | Xuan son virus | Roundleaf bat |
|  | KC344855 | Crimean Congo hemorrhagic | Human |
|  | KC490924 | Anjozorobe hantavirus | Black rat |
|  | KC521440 | Bhanja virus | Goat |
|  | KC631784 | Bowe virus | Doucets musk shrew |
|  | KC880349 | Asikkala virus | Eurasian pygmy shrew |
|  | KC978769 | Sedlec virus | Eurasian reed warbler |
|  | KF153118 | Shuni virus | Horse |
|  | KF254773 | Oriboca virus | Tufted capuchin |
|  | KF297903 | Gabek forest virus | Cairo spiny mouse |
|  | KF543244 | Cumuto virus | Culex mosquito |
|  | KF590574 | Tai virus | Culicidae mosquito |
|  | KF590577 | Kibale virus | Culex mosquito |
|  | KF590586 | Herbert virus | Pig |
|  | KF601560 | Akhtuba virus | Northern pintail |
|  | KF697150 | Manzanilla virus | Venezuelan red howler |
|  | KF697153 | Mermet virus | Purple martin |
|  | KF697157 | Utive virus | Brown throated sloth |
|  | KF711889 | FTLS virus | Human |
|  | KF974361 | Amga virus | Laxmanns shrew |
|  | KF981636 | Khurdun virus | Eurasian coot |
|  | KJ420567 | Imjin virus | Ussuri white toothed shrew |
|  | KJ420576 | Thottapalayam virus | Asian house shrew |
|  | KJ710425 | Abbey lake | Culex mosquito |
|  | KJ857311 | Khabarovsk virus | Maximowiczs vole |
|  | KJ857316 | Yuanjiang virus | Reed vole |
|  | KJ857320 | Kenkeme virus | Flat skulled shrew |
|  | KM102249 | Laibin virus | Black bearded tomb bat |
|  | KM496335 | Anadyr virus | Aedes mosquito |
|  | KM972719 | Tete virus | Village weaver |
|  | KP272043 | Capira virus | Sandfly |
|  | KP731882 | Schmallenberg virus | Cow |
|  | KR828815 | Schmallenberg virus | Blue wildebeest |
|  | KT004447 | Nova virus | European mole |
|  | KT160026 | Capim virus | Bare tailed woolly opossum |
|  | KU936057 | Phasi Charoen virus | Aedes mosquito |
|  | NC_003468 | Andes virus | Human |
|  | NC_004159 | Dugby virus | Human |
|  | NC_005225 | Puumala virus | Human |
|  | NC_005226 | Tula virus | European common vole |
|  | NC_029909 | Erve virus | Human |
| *Caliciviridae* | AF182760 | Porcine enteric virus | Pig |
|  | AF321298 | Walrus calicivirus | Walrus |
|  | AY134748 | Snow mountain virus | Human |
|  | AY144337 | Mink enteric calicivirus | Mink |
|  | AY694184 | Sapovirus | Human |
|  | EF363035 | RHDV | Rabbit |
|  | EF450827 | Norovirus | Lion |
|  | EF650480 | Murine norovirus 5 | Mouse |
|  | EF650481 | Murine norovirus 6 | House mouse |
|  | EU391643 | Tulane virus | Rhesus macaque |
|  | EU980609 | Feline calicivirus | Dog |
|  | FJ692500 | Norovirus | Dog |
|  | GU214989 | Feline calicivirus | Cheetah |
|  | HQ008349 | Bovine enteric virus | Cow |
|  | HQ392821 | Norovirus | Pig |
|  | JF781268 | Norovirus | Cat |
|  | JN204722 | Canine vesivirus | Dog |
|  | JN210887 | Feline calicivirus | Cat |
|  | JN418481 | Nebovirus | Cow |
|  | JN420370 | Sapovirus | California sea lion |
|  | JN975492 | Murine norovirus | Wood mouse |
|  | JQ347522 | Turkey calicivirus | Turkey |
|  | JQ745645 | Recovirus | Human |
|  | JX486102 | Norovirus | Norway rat |
|  | JX627575 | WUHARV Calicivirus 1 | Rhesus macaque |
|  | KC662364 | Recovirus | Rhesus macaque |
|  | KF204570 | Sapovirus | Pig |
|  | KJ577140 | Salmon calicivirus | Atlantic salmon |
|  | KJ641700 | Bat calicivirus | Myotis escalerai |
|  | KJ641701 | Bat calicivirus | Chinese rufous horseshoe bat |
|  | KJ790198 | Bat norovirus | Chinese rufous horseshoe bat |
|  | KJ858687 | Sapovirus chimp | Chimpanzee |
|  | KJ944377 | Feline calicivirus | Tiger |
|  | KM254170 | Chicken caliciviris | Chicken |
|  | KR072992 | Sapovirus fur seal | Fur seal |
|  | KU712497 | Bat calicivirus | Daubentons bat |
|  | KX356908 | Norovirus | Rhesus macaque |
|  | KX357707 | Rabbit calicivirus | Rabbit |
|  | LC011951 | Norovirus | Cat |
|  | NC_002551 | Vesicular exanthema | Pig |
|  | NC_002615 | European brown hare syndrome virus | Hare |
|  | NC_007916 | Newbury agent 1 | Cow |
|  | NC_008580 | Rabbit vesivirus | Rabbit |
|  | NC_011050 | Vesivirus | Steller sea lion |
|  | NC_017936 | Bat sapovirus | Fruit bat |
|  | NC_019712 | Mink calicivirus | Mink |
|  | NC_024078 | Goose calicivirus | Goose |
|  | NC_025676 | San Miguel sea lion virus | California sea lion |
|  | NC_027122 | Vesivirus | Ferret badger |
|  | NC_029647 | Norovirus | Human |
|  | NC_030793 | Bovine calicivirus | Cow |
|  | PCU52086 | Primate calicivirus | Chimpanzee |
|  | RCU52092 | Reptile calicivirus | Rock rattlesnake |
|  | SCU18743 | Skunk calicivirus | Skunk |
|  | SOUCAPPRO | Southampton virus | Human |
|  | X86557 | Lordsdale virus | Human |
| *Coronaviridae* | AF124989 | Human coronavirus OC43 | Human |
|  | AF124991 | Turkey coronavirus | Turkey |
|  | AY686864 | SARS coronavirus | Palm civet |
|  | DQ915164 | Bovine coronavirus isolate Alpaca | Alpaca |
|  | EF424621 | Sable antelope coronavirus | Antelope |
|  | EF424624 | giraffe coronavirus | Giraffe |
|  | FJ376619 | Bulbul coronavirus | Red whiskered bulbul |
|  | FJ376620 | Bulbul coronavirus | Chinese bulbul |
|  | FJ425190 | Sambar deer coronavirus | Sambar deer |
|  | FJ647227 | Murine coronavirus | House mouse |
|  | GU190216 | Bat coronavirus | Daubentons bat |
|  | GU190228 | Bat coronavirus | Mehelys horseshoe bat |
|  | GU190233 | Bat coronavirus | Greater horseshoe bat |
|  | GU190237 | Bat coronavirus | Mediterranean horseshoe bat |
|  | GU190238 | Bat coronavirus | Blasiuss horseshoe bat |
|  | GU190239 | Bat coronavirus | Lesser noctule |
|  | GU553364 | SARS coronavirus | Human |
|  | JF274479 | Infectious bronchitis virus | Chicken |
|  | JN234464 | Human coronavirus HKU1 | Human |
|  | JQ408981 | Feline infectious peritonitis virus | Cat |
|  | JQ731788 | Coronavirus | Great fruit eating bat |
|  | JQ731798 | Coronavirus | Sebas short tailed bat |
|  | JX104161 | Human Coronavirus NL63 | Human |
|  | JX503061 | Human coronavirus 229E | Human |
|  | KC243390 | Bat coronavirus | Soprano pipistrelle |
|  | KC243392 | Bat coronavirus | Nathusiuss pipistrelle |
|  | KC633195 | Betacoronavirus | Parnells mustached bat |
|  | KF294348 | Longquan Aa mouse coronavirus | Brown rat |
|  | KF294356 | Longquan Aa mouse coronavirus | Striped field mouse |
|  | KF294369 | Longquan Rl rat coronavirus | Lesser ricefield rat |
|  | KF294376 | Lushi Ml bat coronavirus | Greater tube nosed bat |
|  | KF294441 | SARS related bat coronavirus | Chinese rufous horseshoe bat |
|  | KF294443 | SARS related bat coronavirus | Pearsons horseshoe bat |
|  | KF294454 | SARS related bat coronavirus | Formosan lesser horseshoe bat |
|  | KF312399 | Betacoronavirus Eptesicus | Serotine bat |
|  | KF650375 | Porcine epidemic diarrhea virus | Pig |
|  | KM454473 | Duck coronavirus | Duck |
|  | KP209313 | MERS coronavirus | Human |
|  | KP696747 | Bat coronavirus | Madagascan flying fox |
|  | KP981644 | Canine coronavirus | Dog |
|  | KR270796 | Porcine respiratory coronavirus | Pig |
|  | KT253289 | Bat coronavirus | Aba roundleaf bat |
|  | KT254278 | Pigeon dominant Coronavirus | Pigeion |
|  | KT368891 | Camel coronavirus HKU23 | Camel |
|  | KU215428 | Feline coronavirus | Cat |
|  | KU343190 | Bat coronavirus | Eastern bent wing bat |
|  | KU343193 | Bat coronavirus | Southeast Asian long fingered bat |
|  | KU343194 | Bat coronavirus | Common bent wing bat |
|  | KU343199 | Bat coroanvirus | Intermediate horseshoe bat |
|  | KU343200 | Bat coronavirus | Pomona roundleaf bat |
|  | KU739074 | Alphacoronavirus | Common shrew |
|  | KU740200 | MERS coronavirus | Camel |
|  | LC061272 | Equine coronavirus | Horse |
|  | LC088095 | Bovine torovirus | Cow |
|  | LC119077 | Ferret coronavirus | Ferret |
|  | LN610099 | Guinea fowl coronavirus | Guinea fowl |
|  | NC_003045 | Bovine coronavirus | Cow |
|  | NC_008516 | White bream virus | White bream |
|  | NC_010646 | Beluga Whale coronavirus SW1 | Beluga whale |
|  | NC_011549 | Thrush coronavirus | Grey backed thrush |
|  | NC_011550 | Munia coronavirus | White rumped munia |
|  | NC_016992 | Sparrow coronavirus | Sparrow |
|  | NC_016993 | Magpie-robin coronavirus | Magpie robin |
|  | NC_016994 | Night heron coronavirus | Night heron |
|  | NC_016995 | Wigeon coronavirus | Wigeon |
|  | NC_016996 | Common moorhen coronavirus | Common moorhen |
|  | NC_017083 | Rabbit coronavirus | European rabbit |
|  | NC_022643 | Betacoronavirus Erinaceus | European hedgehog |
|  | NC_023760 | Mink coronavirus | Mink |
| *Flavivirirdae* | AB114858 | Yokose virus | Eastern bent wing bat |
|  | AB594829 | Japanese encephalitis | Horse |
|  | AF023425 | Pegivirus A | Three striped night monkey |
|  | AF031829 | GB virus C | Chimpanzee |
|  | AF144618 | Pestivirus | Reindeer |
|  | AF285080 | Tamana bat virus | Parnells mustached bat |
|  | AF482341 | Omsk hemorrhagic fever virus | Human |
|  | AF482344 | Omsk hemorrhagic fever virus | Muskrat |
|  | AJ299445 | Montana myotis leukoencephalitis | Little brown bat |
|  | AJ968413 | Dengue virus 2 | Human |
|  | AY149905 | Kamiti River virus | Africana mosquito |
|  | AY277252 | West Nile | Human |
|  | AY632540 | Kedougou virus | Human |
|  | AY701413 | West Nile | Horse |
|  | AY712945 | West Nile | Morning dove |
|  | AY898809 | Alfuy virus | Pheasant coucal |
|  | DQ235150 | Saumarez Reef virus | Sooty tern |
|  | DQ235151 | Turkish sheep encephalitis | Sheep |
|  | DQ235152 | Spanish sheep encephalitis | Sheep |
|  | DQ462443 | Karshi virus | Mouse |
|  | DQ837641 | Entebbe bat virus | Fruit bat |
|  | DQ859062 | Saboya virus | Gambian slit faced bat |
|  | DQ859064 | Spondweni virus | Human |
|  | EF105379 | Dengue virus 2 | Rhesus macaque |
|  | EF623988 | Japanese encephalitis | Human |
|  | FJ462441 | Hepatitis C virus | Human |
|  | FJ606789 | Lammi virus | Culex mosquito |
|  | FJ711167 | Nounane virus | Uranotaenia mosquito |
|  | GU270877 | Border disease virus | Chamois |
|  | HM488177 | West Nile | American crow |
|  | HM488183 | West Nile | Blue jay |
|  | HM488244 | West Nile | American kestrel |
|  | HM582851 | Yellow fever virus | Venezuelan red howler |
|  | HQ231415 | Powassan virus | Human |
|  | HQ235027 | Dengue virus 3 | Human |
|  | JF262780 | Dengue virus 4 | Rhesus macaque |
|  | JF416960 | Kyasanur forest disease virus | Northern plains gray langur |
|  | JF416967 | Alkhumra hemorrhagic fever virus | Human |
|  | JF744991 | Canine hepacivirus | Dog |
|  | JN711458 | Japanese encephalitis virus | Ricketts big footed bat |
|  | JN711459 | Japanese encephalitis virus | Lesser Asiatic yellow bat |
|  | JN860885 | Zika | Human |
|  | JQ219843 | Usutu virus | Blue tit |
|  | JQ289550 | Duck flavivirus | Duck |
|  | JQ582840 | Rio bravo virus | Mexican free tailed bat |
|  | JQ928189 | Tembusu virus | Common shelduck |
|  | JQ957875 | Nienokoue virus | Culex mosquito |
|  | JX123032 | Murray Valley encephalitis | Horse |
|  | JX297521 | Bovine viral diarrhea virus type 1b | Alpaca |
|  | KC145265 | Theilers disease associated virus | Horse |
|  | KC149991 | Classical swine fever virus | Wild boar |
|  | KC181923 | Aedes flavivirus | Aedes mosquito |
|  | KC333651 | Dengue virus 4 | Human |
|  | KC496020 | Barkedji virus | Culex mosquito |
|  | KC505248 | Palm Creek virus | Coquillettidia mosquito |
|  | KC695810 | Bovine viral diarrhea virus 1 | Bactrian camel |
|  | KC796074 | Bat hepacivirus | Striped leaf nosed bat |
|  | KC796083 | Bat pegivirus | Straw coloured fruit bat |
|  | KC796087 | Bat pegivirus | Sebas short tailed bat |
|  | KC796088 | Bat pegivirus | Little yellow shouldered bat |
|  | KC796091 | Bat hepacivirus | Large eared free tailed bat |
|  | KC835597 | Tick-borne encephalitis virus | Bank vole |
|  | KF056331 | Looping ill virus | Sheep |
|  | KF151173 | Tick-borne encephalitis virus | Yellow necked mouse |
|  | KF234529 | Simian pegivirus | Red tailed guenon |
|  | KF234530 | Simian pegivirus | Olive baboon |
|  | KF557893 | Tembusu virus | Wulong goose |
|  | KF711994 | Japanese encephalitis | Pig |
|  | KF751870 | Murray Valley encephalitis | Human |
|  | KF826767 | Tembusu virus | House sparrow |
|  | KF917537 | Israel turkey meningoencephalomyelitis virus | Domestic turkey |
|  | KF917538 | Naranjal virus | Hamster |
|  | KF917539 | Ntaya virus | Grey heron |
|  | KF917541 | Sokoluk virus | Common pipistrelle |
|  | KJ210048 | Nhumirim virus | Culex mosquito |
|  | KJ438715 | Usutu virus | House sparrow |
|  | KJ438755 | Usutu virus | Song thrush |
|  | KJ438781 | Usutu virus | Common black bird |
|  | KJ463423 | Border disease virus | Sheep |
|  | KJ469370 | Batu cave virus | Lesser short nosed fruit bat |
|  | KJ469372 | Phnom Penh bat virus | Greater short nosed fruit bat |
|  | KJ495985 | Looping ill virus | Human |
|  | KJ859683 | Usutu virus | Common pipistrelle |
|  | KJ914682 | Tick-borne encephalitis virus | Eurasian magpie |
|  | KJ950914 | Norway rat pestivirus | Norway rat |
|  | KM066945 | Tembusu virus | Chicken |
|  | KM267635 | St Louis encephalitis virus | Human |
|  | KP144332 | Spanish goat encephalitis virus | Bermeya goat |
|  | KP188568 | Dengue virus 1 | Human |
|  | KP233893 | Entebbe bat virus | Little free tailed bat |
|  | KP296858 | Simian pegivirus | African green monkey |
|  | KP710605 | GB virus C | Human |
|  | KR108246 | Bagaza virus | Red legged partridge |
|  | KR862330 | GB virus | Grivet |
|  | KR866116 | Bovine viral diarrhea virus 1 | Cow |
|  | KR996146 | Simian pegivirus | Yellow baboon |
|  | KT224355 | Negishi virus | Human |
|  | KT934796 | Kunjin virus | Horse |
|  | KU180708 | Pegivirus A | Human |
|  | KU726615 | Culex flavivirus | Culex mosquito |
|  | KX377335 | Zika virus | Rhesus macaque |
|  | NC_001564 | Cell fusing agent virus | Aedes mosquito |
|  | NC_003635 | Modoc virus | White footed deer mouse |
|  | NC_003676 | Apoi virus | Brown rat |
|  | NC_003678 | Pestivirus giraffe | Giraff |
|  | NC_003690 | Langat virus | Tick |
|  | NC_012671 | Quang Binh virus | Culex mosquito |
|  | NC_016997 | Donggang virus | Aedes mosquito |
|  | NC_017086 | Chaoyang virus | Culex mosquito |
|  | NC_021153 | Rodent hepacivirus | Peromyscus maniculatus |
|  | NC_021154 | Rodent pegivirus | Desert woodrat |
|  | NC_023424 | Tyuleniy virus | Common murre |
|  | NC_024018 | Pronghorn antelope pestivirus | Pronghorn antelope |
|  | NC_024377 | Simian pegivirus | Red colobus monkey |
|  | NC_024805 | Ilomantsi virus | Culex mosquito |
|  | NC_024889 | Equine hepacivirus | Horse |
|  | NC_026620 | Jutiapa virus | Hispid cotton rat |
|  | NC_026623 | Cacipacore virus | Black faced antthrush |
|  | NC_026624 | Sokoluk virus | Japanese house bat |
|  | NC_030291 | Pegivirus B | Indian flying fox |
|  | NC_030400 | Nakiwogo virus | Africana mosquito |
|  | NC_030401 | Hanko virus | Culex mosquito |
|  | U94421 | Pegivirus A | White lipped tamarin |
|  | V6182 | West Nile | Double crested cormorant |
|  | V6196 | West Nile | American white pelican |
|  | V6200 | West Nile | Least tern |
|  | V6205 | West Nile | Canadian goose |
|  | V6384 | West Nile | Great horned owl |
|  | V6413 | West Nile | Northern goshawk |
|  | V6441 | West Nile | Red tailed hawk |
|  | V6458 | West Nile | European starling |
|  | V6490 | West Nile | Golden eagle |
|  | V6532 | West Nile | Hawk |
|  | V6536 | West Nile | Herring gull |
|  | V6546 | West Nile | Ring billed gull |
|  | V6561 | West Nile | Short eared owl |
|  | V6562 | West Nile | Swainsons hawk |
|  | V6615 | West Nile | Black vulture |
|  | YFU54798 | Yellowfever | Human |
| *Hepadnaviridae* | 1803562C | Hepatitis B virus | Duck |
|  | AAA19183 | Hepatitis B virus | Woodchuck |
|  | AAD21995 | Hepatitis B virus | Snow goose |
|  | AAF33121 | Hepatitis B virus | Orangutan |
|  | AAO74855 | Hepatitis B virus | Woolly monkey |
|  | CAC80820 | Hepatitis B virus | Stork |
|  | CAD29588 | Hepatitis B virus | Crane |
|  | CCK86644 | Hepatitis B virus | Human |
|  | JX941466 | Hepatitis B virus | Eastern bent wing bat |
|  | KC790377 | Hepatitis B virus | Horseshoe bat |
|  | KC790381 | Hepatitis B virus | Tent making bat |
|  | KR229754 | Hepatitis B virus | White sucker |
|  | KX058433 | Hepatitis B virus | Bluegill |
|  | KX058434 | Hepatitis B virus | African cichlid |
|  | KX058435 | Hepatitis B virus | Tibetan frog |
|  | NC_024443 | Hepatitis B virus | Roundleaf bat |
|  | NP_040994 | Hepatitis B virus | Ground squirrel |
|  | NP_040998 | Hepatitis B virus | Heron |
|  | P12900 | Hepatitis B virus | Chimpanzee |
|  | P87744 | Hepatitis B virus | Gibbon |
|  | YP_004956864 | Hepatitis B virus | Parrot |
|  | YP_024968 | Hepatitis B virus | Rossgoose |
|  | YP_024974 | Hepatitis B virus | Sheldgoose |
| *Herpesviridae* | AF141887 | Tapirus terrestris gammaherpesvirus 1 | South American tapir |
|  | AF204167 | Macaca nemestrina rhadinovirus 2 | Southern pig tailed macaque |
|  | AF236050 | California sea lion herpesvirus | California sea lion |
|  | AF239684 | Green turtle herpesvirus | Turtle |
|  | AF376034 | Badger herpesvirus | Badger |
|  | AF478169 | Porcine lymphotropic herpesvirus | Pig |
|  | AF520812 | Passerid herpesvirus 1 | Gouldian finch |
|  | AF534227 | Mandrillus sphinx lymphocryptovirus 1 | Mandrill |
|  | AJ224971 | Feline herpesvirus 1 | Cat |
|  | AY129398 | Pongo pygmaeus lymphocryptovirus 1 | Orangutan |
|  | AY139026 | Callithrix penicillata lymphocryptovirus 1 | Black tufted marmoset |
|  | AY139028 | Ateles paniscus lymphocryptovirus 1 | Red faced spider monkey |
|  | AY177144 | Gorilla rhadinovirus 1 | Gorilla |
|  | AY197559 | Hexaprotodon liberiensis gammaherpesvirus 1 | Pygmy hippopotamus |
|  | AY197560 | Diceros bicornis gammaherpesvirus 1 | Black rhinoceros |
|  | AY197561 | Tupaia belangeri gammaherpesvirus 1 | Northern treeshrew |
|  | AY495965 | Equus zebra gammaherpesvirus 1 | Zebra |
|  | AY646889 | Fibropapilloma associated turtle herpesvirus | Loggerhead sea turtle |
|  | AY949827 | Canine herpesvirus | Dog |
|  | AY949828 | Blainvilles beaked whale gammaherpesvirus | Blainvilles beaked whale |
|  | AY949830 | Dwarf sperm whale gammaherpesvirus | Dwarf sperm whale |
|  | AY949831 | Bottlenose dolphin gammaherpesvirus | Bottlenose dolphin |
|  | DQ198083 | Ovine herpesvirus 2 | Sheep |
|  | DQ789369 | Rupicapra rupicapra gammaherpesvirus 1 | Chamois |
|  | DQ789370 | Panthera leo gammaherpesvirus 1 | Lion |
|  | DQ789371 | Crocuta crocuta gammaherpesvirus 1 | Spotted hyena |
|  | DQ821581 | Bandicota savilei rhadinovirus 1 | Saviles bandicoot rat |
|  | EF125063 | Apodemus flavicollis cytomegalovirus 2 | Yellow necked mouse |
|  | EU085377 | Macaca fascicularis rhadinovirus 2 | Crab eating macaque |
|  | EU085380 | Sorex araneus gammaherpesvirus 1 | Common shrew |
|  | EU118146 | Papio hamadryas lymphocryptovirus 3 | Hamadryas baboon |
|  | EU579860 | Muromegalovirus | Mouse |
|  | FJ538485 | Pan troglodytes cytomegalovirus | Chimpanzee |
|  | FJ538490 | Gorilla gorilla cytomegalovirus | Gorilla |
|  | GQ169129 | Wood mouse herpesvirus | Mouse |
|  | GQ921924 | Symphalangus syndactylus lymphocryptovirus 2 | Siamang |
|  | HM216460 | Ovine herpesvirus 2 | Pere Davids deer |
|  | HM216467 | Ovine herpesvirus 2 | Sika deer |
|  | HM216474 | Caprine herpesvirus 2 | Goat |
|  | HSKUL30H | Gallid herpesvirus 2 | Chicken |
|  | JN692430 | Myotis ricketti herpesvirus 2 | Bat |
|  | JQ673560 | Anatid herpesvirus 1 | Duck |
|  | JQ692312 | Equid herpesvirus 1 | Polar bear |
|  | JQ809328 | Suid herpesvirus 1 | Wild boar |
|  | JX195700 | Equid herpesvirus 9 | Zebra |
|  | KC618527 | Elephant endotheliotropic herpesvirus 1A | Elephant |
|  | KC924783 | Equid herpesvirus 1 | Horse |
|  | KF466473 | Harp seal herpesvirus | Seal |
|  | KJ191540 | Beluga whale alphaherpesvirus 1 | Beluga whale |
|  | KM258880 | Bovine herpesvirus | Cow |
|  | KM438002 | Bovine herpesvirus 6 | Water buffalo |
|  | KT698106 | Cheirogaleid herpesvirus 2 | Gray mouse lemur |
|  | NC_000898 | Human herpesvirus 6B | Human |
|  | NC_001664 | Human herpesvirus 6A | Human |
|  | NC_001716 | Human herpesvirus 7 | Human |
|  | NC_001798 | Human herpesvirus 2 | Human |
|  | NC_001806 | Human herpesvirus 1 | Human |
|  | NC_002531 | Alcelaphine herpesvirus 1 | Blue wildebeest |
|  | NC_004367 | Callitrichine herpesvirus 3 | Common marmoset |
|  | NC_004812 | Macacine herpesvirus 1 | Rhesus monkey |
|  | NC_007605 | Human herpesvirus 4 | Human |
|  | NC_009333 | Human herpesvirus 8 | Human |
|  | NC_012783 | Cercopithecine herpesvirus 5 | Grivet |
|  | NC_015049 | Cricetid herpesvirus 2 | Rat |
|  | NC_016447 | Aotine herpesvirus 1 | Three striped night monkey |
|  | NC_016448 | Saimiriine herpesvirus 4 | Common squirrel monkey |
|  | NC_024382 | Alcelaphine herpesvirus 2 | Topi |
| *Orthomyxoviridae* | AB731583 | Influenza A H1N2 | Pig |
|  | AY582057 | Influenza B virus | Human |
|  | CY043862 | Influenza A H9N2 | Eurasian Wigeon |
|  | CY063991 | Influenza A virus | Gadwall |
|  | CY064175 | Influenza A H10N7 | Ring necked duck |
|  | CY076651 | Influenza A H6N1 | Goose |
|  | CY077639 | Influenza A H4N8 | Red necked Stint |
|  | CY094909 | Influenza A H13N6 | Silver gull |
|  | CY095106 | Influenza A H7N2 | Chicken |
|  | CY103874 | Influenza A H17N10 | Little yellow shouldered bat |
|  | CY125943 | Influenza A H18N11 | Flat faced bat |
|  | CY130108 | Influenza A H15N9 | Shearwater |
|  | CY134245 | Influenza A H4N1 | Mallard |
|  | CY144184 | Influenza A H16N3 | Herring gull |
|  | CY144689 | Influenza A H7N3 | Sanderling |
|  | CY149490 | Influenza A H10N5 | Black headed gull |
|  | CY149506 | Influenza A H6N5 | Goose |
|  | CY149578 | Influenza A H10N7 | Black scoter |
|  | CY167100 | Influenza A H4N8 | Common goldeneye |
|  | CY167198 | Influenza A H5N3 | Green wing teal |
|  | CY172461 | Influenza A H3N2 | Human |
|  | CY178992 | Influenza A H4N6 | Snipe |
|  | CY179081 | Influenza A H1N6 | Common murre |
|  | CY179129 | Influenza A H1N6 | Horned puffin |
|  | CY179833 | Influenza A H6N1 | Canada goose |
|  | CY185567 | Influenza A H9N3 | Mediterranean gull |
|  | CY185639 | Influenza A H13N8 | Yellow legged gull |
|  | CY185859 | Influenza A H7N3 | Shorebird |
|  | CY185893 | Influenza A H15N9 | Wedge tailed shearwater |
|  | CY185901 | Influenza A H7N7 | Red knot |
|  | CY185919 | Influenza A H7N2 | Ruddy turnstone |
|  | CY185975 | Influenza A H7N1 | Blue wing teal |
|  | CY186435 | Influenza A | Bufflehead |
|  | CY187067 | Influenza A H10N7 | Redhead |
|  | CY196641 | Influenza A H4N6 | Northern shoveler |
|  | CY202775 | Influenza A H1N1 | Snow goose |
|  | CY204360 | Influenza A H5N2 | Green wing teal |
|  | CY206657 | Influenza A H4N6 | Blue wing teal |
|  | CY206956 | Influenza A H16N3 | Glaucous gull |
|  | DHVPB1 | Dhori virus | Tick |
|  | DQ124148 | Influenza A H3N8 | Dog |
|  | DQ124176 | Influenza A H3N8 | Horse |
|  | DQ145544 | Influenza A H3N1 | Pig |
|  | DQ822196 | Influenza A H6N8 | Bewicks swan |
|  | EU182279 | Influenza A H9N2 | Turkey |
|  | EU277847 | Influenza A H5N1 | Guinea fowl |
|  | FJ519989 | Influenza A H5N2 | Ostrich |
|  | FJ861695 | Quaranfil virus | Tick |
|  | FJ861697 | Johnston Atoll virus | Tick |
|  | FLAPB1PROB | Influenza A H2N2 | Human |
|  | GQ229276 | Influenza A H1N1 | Wild boar |
|  | GU050000 | Influenza A H5N2 | Northern pintail |
|  | GU051992 | Influenza A H5N9 | Glaucous gull |
|  | GU052305 | Influenza A H3N8 | American Black duck |
|  | GU052874 | Influenza A H5N9 | Mallard |
|  | HQ259672 | Infectious salmon anemia virus | Atlantic salmon |
|  | JN247617 | Influenza A H3N2 | Dog |
|  | JQ794460 | Influenza A H5N1 | Chicken |
|  | JQ928944 | Tjuloc virus | Argas arboreus tick |
|  | JQ973646 | Influenza A H7N1 | Baers pochard |
|  | JX307162 | Influenza A H7N4 | Duck |
|  | JX473008 | Influenza A H1N1 | Human |
|  | JX865420 | Influenza A H1N1 | Elephant seal |
|  | KC422454 | Influenza A H3N2 | Cat |
|  | KF260564 | Influenza A H9N2 | Pheasant |
|  | KF260695 | Influenza A H7N7 | Pigeon |
|  | KF609525 | Influenza A H7N9 | Tree sparrow |
|  | KJ372718 | Influenza A H13N2 | Pilot whale |
|  | KJ467570 | Influenza A H3N8 | Harbour seal |
|  | KJ847693 | Influenza A H4N6 | Caspian seal |
|  | KM015492 | Influenza D virus | Cow |
|  | KM507546 | Influenza C virus | Human |
|  | KP732605 | Influenza A H5N8 | Goose |
|  | KU201902 | Influenza A H5N8 | Peregrine falcon |
|  | KU708254 | Bourbon virus | Human |
|  | LC010982 | Thogoto virus | Tick |
|  | NC_025796 | Wellfleet bay virus | Common eider |
| *Papillomaviridae* | AF020905 | Papillomavirus 1 | Chimpanzee |
|  | AF443292 | Reindeer papillomavirus | Reindeer |
|  | AF502599 | Thetapapillomavirus 1 | African gray parrot |
|  | AJ243287 | Kappapapillomavirus 2 | Rabbit |
|  | AY904722 | Lynx rufus papillomavirus type 1 | Bobcat |
|  | AY904723 | Puma concolor papillomavirus type 1 | Florida panther |
|  | AY904724 | Panthera leo persica papillomavirus type 1 | Asian lion |
|  | DQ098913 | Bovine papillomavirus type 8 | Cow |
|  | DQ098917 | Bovine papillomavirus type 8EB | European bison |
|  | DQ180494 | Uncia uncia papillomavirus type 1 | Snow leopard |
|  | EF536349 | Ursus maritmus | Polar bear |
|  | EF546482 | Human papillomavirus type 53 | Human |
|  | EF591299 | Macaca fascicularis papillomavirus type 3b | Crab eating macaque |
|  | EF591300 | Rhesus papillomavirus type 1b | Crab eating macaque |
|  | EU490516 | Papillomavirus type 9 | Crab eating macaque |
|  | EU493091 | Chelonia mydas papillomavirus 1 | Green sea turtle |
|  | EU796884 | Feline papillomavirus 2 | Cat |
|  | FJ492742 | Canine papillomavirus 7 | Dog |
|  | FJ796965 | Ovine papillomavirus 3 | Sheep |
|  | GU117620 | Delphinus delphis papillomavirus | Short beaked common dolphin |
|  | GU117624 | Lagenorhynchus acutus papillomavirus | Atlantic white sided dolphin |
|  | HQ912791 | Camelus dromedarius papillomavirus type 2 | Camel |
|  | JN644142 | Human papillomavirus type 11 | Human |
|  | JQ692938 | Miniopterus schreibersii papillomavirus 1 | Common bent wing bat |
|  | JQ798171 | Bovine papillomavirus type 13 | Cow |
|  | JX174442 | Bos grunniens papillomavirus type 1 | Yak |
|  | JX972168 | Felis catus papillomavirus 3 | Cat |
|  | KC858266 | Rhinolophus ferrumequinum papillomavirus type 1 | Greater horseshoe bat |
|  | KF857586 | Vulpes vulpes papillomavirus 1 | Red fox |
|  | KP099578 | Papillomavirus type 1 | Djungarian hamster |
|  | KP861981 | Alouatta guariba papillomavirus 1 | Southern brown howler |
|  | KT626573 | Deltapapillomavirus 5 | Visayan spotted deer |
|  | KT944080 | Macaca fuscata papillomavirus 1 | Japanese macaque |
|  | KU053944 | Alphapapillomavirus 9 | Human |
|  | KU248828 | Papillomavirus JL74 | Greater long tailed hamster |
|  | KU519394 | Bovine papillomavirus 19 | Cow |
|  | KU684317 | Human papillomavirus type 16 | Human |
|  | NC_001354 | Human papillomavirus type 41 | Human |
|  | NC_001356 | Human papillomavirus 1 | Human |
|  | NC_001458 | Human papillomavirus 63 | Human |
|  | NC_001524 | European elk papillomavirus | Elk |
|  | NC_001531 | Human papillomavirus type 5 | Human |
|  | NC_001576 | Human papillomavirus type 10 | Human |
|  | NC_001605 | papillomavirus | Natal multimammate mouse |
|  | NC_001619 | Lambdapapillomavirus 2 | Dog |
|  | NC_001678 | Rhesus monkey papillomavirus | Rhesus monkey |
|  | NC_001789 | Ovine papillomavirus 1 | Sheep |
|  | NC_002232 | Kappapapillomavirus 1 | Rabbit |
|  | NC_003348 | Omikronpapillomavirus 1 | Burmeisters porpoise |
|  | NC_003748 | Equus caballus papillomavirus 1 | Horse |
|  | NC_004068 | Fringilla coelebs papillomavirus | Common chaffinch |
|  | NC_004765 | Felis catus papillomavirus 1 | Cat |
|  | NC_006951 | Erethizon dorsatum papillomavirus 1 | North American porcupine |
|  | NC_007612 | Bovine papillomavirus 7 | Cow |
|  | NC_008032 | Papillomavirus 1 | Goat |
|  | NC_008298 | Rousettus aegyptiacus papillomavirus 1 | Egyptian fruit bat |
|  | NC_008582 | Micromys minutus papillomavirus 1 | European harvest mouse |
|  | NC_011051 | Capreolus capreolus papillomavirus 1 | Roedeer |
|  | NC_011109 | Tursiops truncatus papillomavirus 1 | Bottlenose dolphin |
|  | NC_011280 | sus scrofa Papillomavirus 1 | Pig |
|  | NC_011530 | Caretta caretta papillomavirus 1 | Loggerhead sea turtle |
|  | NC_011765 | Erinaceus europaeus papillomavirus 1 | Western European hedgehog |
|  | NC_012123 | Equine papillomavirus 2 | Horse |
|  | NC_013117 | Francolinus leucoscepus papillomavirus 1 | Yellow necked spurfowl |
|  | NC_013237 | Canis familiaris papillomavirus 6 | Dog |
|  | NC_014143 | Bettongia penicillata papillomavirus 1 | Woylie |
|  | NC_014326 | Mus musculus papillomavirus type 1 | House mouse |
|  | NC_014469 | Gammapapillomavirus | Human |
|  | NC_015325 | Zalophus californianus papillomavirus 1 | Californian sea lion |
|  | NC_015691 | Macaca fascicularis papillomavirus 2 | Crab eating macaque |
|  | NC_015692 | Colobus guereza papillomavirus type 2 | Mantled guereza |
|  | NC_016013 | Morelia spilota papillomavirus 1 | Carpet Python |
|  | NC_016898 | Trichechus manatus latirostris papillomavirus 2 | West Indian manatee |
|  | NC_017716 | Papio hamadryas papillomavirus type 1 | Baboon monkey |
|  | NC_017862 | Equine papillomavirus 3 | Horse |
|  | NC_018074 | Phocoena phocoena papillomavirus 1 | Harbour porpoise |
|  | NC_018076 | Phocoena phocoena papillomavirus 4 | Harbour porpoise |
|  | NC_022253 | Ferret papillomavirus | Ferret |
|  | NC_022373 | Felis catus papillomavirus 4 | Cat |
|  | NC_022647 | Mesocricetus auratus papillomavirus 1 | Golden hamster |
|  | NC_023178 | Castor canadensis papillomavirus 1 | North American beaver |
|  | NC_023496 | Saimiri sciureus papillomavirus 1 | Squirrel monkey |
|  | NC_023852 | Canine papillomavirus 13 | Dog |
|  | NC_023873 | Enhydra lutris papillomavirus 1 | Sea otter |
|  | NC_023882 | Equus asinus papillomavirus | Donkey |
|  | NC_023894 | Pygoscelis adeliae papillomavirus | Adelie penguin |
|  | NC_023895 | Rupicapra rupicapra papillomavirus 1 | Chamois |
|  | NC_024300 | Fulmarus glacialis papillomavirus 1 | Northern fulmar |
|  | NC_024893 | Apodemus sylvaticus papillomavirus 1 | Wood mouse |
|  | NC_026640 | Canine papillomavirus 16 | Dog |
|  | NC_028267 | Trichechus manatus papillomavirus 4 | Florida manatee |
|  | NC_028492 | Rattus norvegicus papillomavirus 3 | Brown rat |
|  | NC_030151 | Cervus papillomavirus 2 | Red deer |
|  | NC_030839 | Sparus aurata papillomavirus 1 | Gilt head bream |
|  | PPDCG | Deer papillomavirus | White tailed deer |
| *Paramyxoviridae* | AB605247 | Newcastle | Chicken |
|  | AB844350 | Paramyxovirus | White toothed shrew |
|  | AB844369 | Rodent Paramyxovirus | Natal multimammate mouse |
|  | AF092942 | Respiratory syncytial virus | Cow |
|  | AF286043 | Snake paramyxovirus | Bush viper |
|  | AF286045 | Snake paramyxovirus | Neotropical rattlesnake |
|  | AF349405 | Reptilian paramyxovirus | Western diamondback rattlesnake |
|  | AF349408 | Reptilian paramyxovirus | Corn snake |
|  | AJ608288 | Dolphin morbillivirus | Dolphin |
|  | DQ172679 | Paramyxovirus | Pacific salmon |
|  | FJ215864 | Avian paramyxovirus 8 | Pintail duck |
|  | GQ277613 | Snake paramyxovirus | Montane egg eating snake |
|  | GQ277614 | Lizard paramyxovirus | Flathead knob scaled lizard |
|  | GQ277615 | Paramyxovirus | Hermanns tortoise |
|  | GQ277616 | Paramyxovirus | Beauty snake |
|  | GQ277617 | Paramyxovirus | Green iguana |
|  | GQ288381 | Newcastle disease virus | Cormorant |
|  | GU726899 | Paramyxovirus | Leopard tortoise |
|  | HE647823 | Eidolon paramyxovirus | Strawcoloured fruit bat |
|  | HG934338 | Avian pneumovirus | Muscovy duck |
|  | HM044321 | Hendra virus | Horse |
|  | HM159994 | Avian paramyxovirus 2 | Gadwell |
|  | HM159995 | Avian paramyxovirus 2 | Finch |
|  | HM439386 | Measles | Human |
|  | HM852904 | Canine distemper virus | Rhesus macaque |
|  | HQ660095 | Bat mumps virus | Wahlbergs epauletted fruit bat |
|  | HQ687899 | Avian paramyxovirus | Penguin |
|  | HQ896024 | Avian paramyxovirus 2 | Chicken |
|  | JF424833 | Avian pneumovirus | Turkey |
|  | JF939201 | Peste des petits ruminants virus | Goat |
|  | JN255801 | Hendra virus | Bat |
|  | JN255805 | Hendra virus | Human |
|  | JN808863 | Nipah | Human |
|  | JQ001776 | Cedar virus | Large flying fox |
|  | JQ388690 | Mumps | Human |
|  | JQ743328 | Parainfluenza 5 | Pig |
|  | JQ886098 | Paramyxovirus | Triaenops menamena bat |
|  | JQ886099 | Paramyxovirus | Miniopterus griveaudi bat |
|  | KC802221 | Phocine distemper virus | Harbour seal |
|  | KC853020 | Newcastle disease virus | Crested ibis |
|  | KF278639 | Mojiang virus | Sladens Rat |
|  | KF640637 | Respiratory syncytial virus | Human |
|  | KF640687 | Canine distemper virus | Dog |
|  | KF740478 | Newcastle disease virus | Japanese quail |
|  | KF871300 | Paramyxovirus | Grey headed flying fox |
|  | KF928254 | Bat paramyxovirus | Malagasy white bellied free tailed bat |
|  | KF928263 | Bat paramyxovirus | Madagascar free tailed bat |
|  | KF928264 | Bat paramyxovirus | Malagasy mouse eared bat |
|  | KJ147057 | Canine distemper virus | Racoon |
|  | KJ748514 | Bat paramyxovirus | Free tailed bat |
|  | KJ843144 | Caprine parainfluenza virus 3 | Goat |
|  | KJ956408 | Paramyxovirus | Green anaconda |
|  | KP762799 | Avian paramyxovirus 6 | Red crested pochard |
|  | KR732614 | Newcastle disease virus | Indian peafowl |
|  | KT071755 | Avian paramyxovirus 2 | Dark breasted rosefinch |
|  | KT071756 | Avian paramyxovirus 2 | Paddyfield pipit |
|  | KT071757 | Avian paramyxovirus 2 | Black faced bunting |
|  | KT426535 | Rubulavirus | Pig |
|  | KX100034 | Parainfluenza 5 | Red panda |
|  | KX119151 | Avian paramyxovirus 13 | White fronted goose |
|  | KX236100 | Newcastle disease virus | Pigeon |
|  | LC036586 | Feline morbillivirus | Cat |
|  | NC_003443 | Parainfluenza 2 | Human |
|  | NC_004074 | Tioman virus | Small flying fox |
|  | NC_005084 | Far de lance virus | Bothrops asper |
|  | NC_005339 | Mossman virus | Wild rat |
|  | NC_006428 | Simian virus 41 | Crab eating macaque |
|  | NC_007454 | J virus | House mouse |
|  | NC_009489 | Mapuera virus | Little yellow shouldered bat |
|  | NC_017937 | Nariva virus | Short tailed cane mouse |
|  | NC_021928 | Parainfluenza 4 | Human |
|  | NC_025347 | Avian paramyxovirus 7 | Dove |
|  | NC_025355 | Tailam virus | Sikkim rat |
|  | NC_025360 | Paramyxovirus | Atlantic salmon |
|  | NC_025361 | Avian paramyxovirus 5 | Budgerigar |
|  | NC_025374 | Avian paramyxovirus 8 | Canada goose |
|  | NC_025386 | Salem virus | Horse |
|  | NC_025407 | Avian paramyxovirus 11 | Common snipe |
|  | NC_025410 | Tuhoko virus | Fruit bat |
|  | PAFZSTR | Sendai virus | Mouse |
|  | X98291 | Rinderpest virus | Buffalo |
| *Parvoviridae* | AB982222 | Bufavirus 3 | Human |
|  | AF221123 | Pig tailed macaque parvovirus | Pig tailed macaque |
|  | AJ249557 | feline parvovirus | Cheetah |
|  | AY349010 | Snake adeno-associated virus | Snake |
|  | AY461507 | Mythimna loreyi densovirus | False Army Worm |
|  | AY506547 | Goose parvovirus | Goose |
|  | AY695374 | Adeno associated virus | Human |
|  | AY768535 | Human parvovirus B19 | Human |
|  | DQ100362 | Mouse adeno associated virus 1 | Mouse |
|  | DQ100363 | Rat adeno associated virus 1 | Brown rat |
|  | DQ196318 | Mouse parvovirus 3 | Mouse |
|  | DQ196319 | Mouse parvovirus 2 | Mouse |
|  | DQ335247 | Bovine parvovirus 1 | Cow |
|  | DQ340434 | Canine parvovirus | Dog |
|  | EF418569 | Feline panleukopenia virus | Lion |
|  | EF515837 | Goose parvovirus | Muscovy duck |
|  | EU145593 | Feline panleukopenia virus | Asian palm civet |
|  | EU659114 | Feline panleukopenia virus | Mountain lion |
|  | EU698028 | Blue fox parvovirus | Blue fox |
|  | EU872429 | Serpentine adeno associated virus 2 | Pit viper |
|  | FJ214110 | Canine minute virus | Dog |
|  | FJ231389 | Feline panleukopenia virus | Rhesus monkey |
|  | FJ405225 | Feline panleukopenia virus | Tiger |
|  | FJ688147 | Adeno associated virus | Pig |
|  | GQ368252 | Avian adeno associated virus | Chicken |
|  | GU048663 | Human bocavirus 2 | Human |
|  | GU048665 | Human bocavirus 3 | Human |
|  | GU214704 | Chicken parvovirus | Chicken |
|  | HM053672 | Bocavirus pig 6 | Pig |
|  | HM053673 | Bocavirus pig 7 | Pig |
|  | HQ223038 | Pig bocavirus | Pig |
|  | J02275 | Minute virus of mice | Mouse |
|  | JF504697 | Bovine hokovirus 2 | Cow |
|  | JF504702 | Ovine hokovirus | Sheep |
|  | JN040434 | Aleutian mink disease virus | Mink |
|  | JN202450 | Gray fox amdovirus | Gray fox |
|  | JN420364 | California sea lion bocavirus 1 | California sea lion |
|  | JN420365 | California sea lion bocavirus 3 | California sea lion |
|  | JN420366 | California sea lion bocavirus 2 | California sea lion |
|  | JN420372 | California sea lion adeno associated virus 1 | California sea lion |
|  | JN798195 | Human parvovirus 4 | Human |
|  | JN798203 | Parvovirus 4 like | Chimpanzee |
|  | JN798208 | Parvovirus 4 like | Western red colobus |
|  | JN798211 | Parvovirus 4 like | King colobus |
|  | JN867617 | Canine parvovirus | Raccoon |
|  | JN990269 | Porcine partetravirus | Pig |
|  | JQ249926 | Porcine parvovirus | Wild boar |
|  | JQ249927 | Porcine parvovirus | Pig |
|  | JX411926 | Canine parvovirus | Stone marten |
|  | JX475241 | Canine parvovirus | Mexican wolf |
|  | JX475288 | Canine parvovirus | Striped skunk |
|  | JX627317 | Rat minute virus | Brown rat |
|  | JX827169 | Rat parvovirus 1 | Brown rat |
|  | KC154061 | Batparvovirus | Common bent wing bat |
|  | KC339251 | Bat bocavirus | Eastern bent wing bat |
|  | KC478066 | Goose parvovirus | Swan |
|  | KC580640 | Canine bocavirus 3 | Dog |
|  | KC692368 | Fox parvovirus | Red fox |
|  | KC883978 | Junonia coenia densovirus | Common buckeye |
|  | KF225551 | Porcine hokovirus | Pig |
|  | KF373759 | Seal parvovirus | Harbor seal |
|  | KF661535 | Porcine parvovirus 5 | Pig |
|  | KJ396349 | Raccoon dog amdovirus | Arctic fox |
|  | KJ641663 | Bat parvovirus | Blyths horseshoe bat |
|  | KJ641665 | Bat parvovirus | Ricketts big footed bat |
|  | KJ641666 | Bat parvovirus | Pomona roundleaf bat |
|  | KJ641669 | Bat parvovirus | Eastern bent wing bat |
|  | KJ641671 | Bat parvovirus | Greater horseshoe bat |
|  | KJ641680 | Bat parvovirus | Daubentons bat |
|  | KJ641681 | Bat parvovirus | Beijing mouse eared bat |
|  | KJ641683 | Bat parvovirus | Chinese noctule |
|  | KJ813832 | Canine parvovirus | Fisher |
|  | KJ813837 | Canine parvovirus | River otter |
|  | KJ813863 | Canine parvovirus | Cougar |
|  | KJ813886 | Canine parvovirus | Coyote |
|  | KJ813893 | Feline panleukopenia virus | Bobcat |
|  | KJ813894 | Feline parvovirus | Raccoon |
|  | KM254173 | Protoparvovirus HK2014 | Chicken |
|  | KM580351 | Bufavirus 1 | Human |
|  | KM598417 | Gallus gallus enteric parvovirus | Chicken |
|  | KM598421 | Meleagris gallopavo enteric parvovirus | Turkey |
|  | KP019621 | Feline panleukopenia virus | Small Indian civet |
|  | KP033233 | Feline panleukopenia virus | African black footed cat |
|  | KP033243 | Feline panleukopenia virus | Ocelot |
|  | KP264981 | Bovine adeno associated virus | Cow |
|  | KP682520 | Feline panleukopenia virus | European badger |
|  | KP682526 | Feline panleukopenia virus | Beech marten |
|  | KP733795 | Corn snake parvovirus | Corn snake |
|  | KP733796 | Pygmy chameleon parvovirus | Short tailed pygmy chameleon |
|  | KP769859 | Feline panleukopenia virus | Cat |
|  | KR002805 | Canine parvovirus 2a | Dog |
|  | KR004179 | Protoparvovirus Udayana | Muscovy duck |
|  | KR014516 | Human bocavirus 1 | Human |
|  | KR265066 | Goose parvovirus | White goose |
|  | KT223502 | Primate bocaparvovirus 1 | Chimpanzee |
|  | KT268312 | Primate erythroparvovirus 1 | Human |
|  | KT343253 | Duck parvovirus | Cherry valley duck |
|  | KT751090 | Goose parvovirus | Peking duck |
|  | KU172423 | Bovine hokovirus 2 | Cow |
|  | KU569162 | Galliform aveparvovirus 1 | Chicken |
|  | KU867071 | Porcine bufavirus | Pig |
|  | LC090199 | Ungulate bocaparvovirus 3 | Pig |
|  | NC_001701 | Goose parvovirus | Greylag goose |
|  | NC_001899 | Diatraea saccharalis densovirus | Sugarcane Borer |
|  | NC_004287 | Bombyx mori densovirus 5 | Silkworm |
|  | NC_004295 | Human erythrovirus V9 | Human |
|  | NC_006260 | Adeno associated virus 7 | Rhesus monkey |
|  | NC_006261 | Adeno associated virus 8 | Rhesus monkey |
|  | NC_012685 | Culex pipiens densovirus | Culex mosquito |
|  | NC_012729 | Human bocavirus 4 | Human |
|  | NC_014358 | Bocavirus gorilla | Gorilla |
|  | NC_014468 | Bat adeno associated virus | Ricketts big footed bat |
|  | NC_015718 | Helicoverpa armigera densovirus | Cotton bollworm |
|  | NC_016031 | Porcine bocavirus 3 | Pig |
|  | NC_016032 | Porcine bocavirus 4 | Pig |
|  | NC_016647 | Porcine bocavirus 5 | Pig |
|  | NC_016744 | Eidolon helvum parvovirus 1 | Straw coloured fruit bat |
|  | NC_016752 | Artibeus jamaicensis parvovirus 1 | Jamaican fruit bat |
|  | NC_019492 | Pseudoplusia includens densovirus | Soybean Looper |
|  | NC_020499 | Canine bocavirus 1 | Dog |
|  | NC_022800 | Feline bocavirus 2 | Cat |
|  | NC_023842 | Danaus plexippus plexippus iteravirus | Monarch butterfly |
|  | NC_024454 | Turkey parvovirus | Turkey |
|  | NC_025825 | Raccoon dog amdovirus | Raccoon dog |
|  | NC_025891 | Slow loris parvovirus 1 | Slow loris |
|  | NC_026251 | Sesavirus | Sea lion |
|  | NC_026815 | Mpulungu bufavirus | Lesser red musk shrew |
|  | NC_027429 | Bearded dragon parvovirus | Bearded dragon |
|  | NC_028136 | Ungulate tetraparvovirus 1 | Yak |
|  | NC_028650 | Rat bufavirus | Black rat |
|  | NC_028973 | Rabbit bocaparvovirus | Rabbit |
|  | NC_029133 | Rat bocavirus | Brown rat |
|  | NC_029797 | Megabat bufavirus 1 | Large flying fox |
|  | NC_030837 | Sea otter parvovirus 1 | Sea otter |
|  | U22185 | Feline panleukopenia virus | Arctic fox |
|  | U22189 | Feline panleukopenia virus | Wildcat |
|  | U22191 | Mink enteritis virus | American mink |
|  | U22193 | Canine parvovirus | Raccoon dog |
|  | U26342 | Simian parvovirus | Crab eating macaque |
|  | U79033 | Kilham rat virus | Brown rat |
|  | U86868 | Chipmunk parvovirus | Chipmunk |
|  | X75093 | Muscovy duck parvovirus | Muscovy duck |
| *Picornaviridae* | AF326750 | Baboon enterovirus | Baboon |
|  | DQ404174 | Foot and mouth | Sheep |
|  | EU140838 | Simian hepatitis A | Rhesus macaque |
|  | EU236594 | Bovine rhinovirus | Cow |
|  | EU681179 | Cardiovirus | Human |
|  | EU787450 | Porcine kobuvirus | Pig |
|  | EU789367 | Simian sapelovirus | Human |
|  | FJ007373 | Enterovirus J | Southern pig tailed macaque |
|  | GQ415052 | Rhinovirus A | Human |
|  | GU109481 | Coxsackievirus B3 | Sichuan snub nosed monkey |
|  | GU182406 | Passerivirus A1 | Pale Thrush |
|  | HM153767 | Ovine hungarovirus | Sheep |
|  | HPAACG | Hepatitis A | Human |
|  | HQ020378 | Teschovirus | Pig |
|  | HQ246217 | Hepatitis A | Chimpanzee |
|  | HQ415759 | Enterovirus C96 | Human |
|  | HQ595341 | Bat picornavirus 1 | Common bent wing bat |
|  | HQ595345 | Bat picornavirus 3 | Great roundleaf bat |
|  | HQ832582 | Foot and mouth disease virus | Buffalo |
|  | HQ917060 | Bovine enterovirus type 2 | Cow |
|  | JN205461 | Rhinovirus C | Human |
|  | JN379039 | Salivirus CH virus | Chimpanzee |
|  | JN979570 | coxsackievirus B3 | Chimpanzee |
|  | JQ277724 | Ovine enterovirus | Sheep |
|  | JQ814852 | Bat picornavirus 1 | Great evening bat |
|  | JX177612 | Porcine kobuvirus | Wild boar |
|  | JX441355 | Human parechovirus 1 | Human |
|  | JX481738 | Coxsackievirus A16 | Human |
|  | JX627574 | Sapelovirus | Rhesus macaque |
|  | JX678288 | Human enterovirus C118 | Human |
|  | JX683808 | Cardiovirus | Norway rat |
|  | KC161964 | Canine kobuvirus | Dog |
|  | KC560801 | Pigeon picornavirus B | Pigeon |
|  | KC748420 | Enterovirus F | Alpaca |
|  | KC993890 | Duck hepatitis A virus 3 | Duck |
|  | KF293299 | Encephalomyocarditis virus | South china tiger |
|  | KF541640 | Coxsackievirus A24 | Gorilla |
|  | KF541641 | Echovirus E15 | Gorilla |
|  | KF831027 | Feline kobuvirus | Cat |
|  | KF836387 | Encephalomyocarditis virus | Boar |
|  | KF874490 | Fathead minnow picornavirus | Fathead minnow |
|  | KJ170677 | Human poliovirus 3 | Human |
|  | KJ641696 | Bat picornavirus | Asian particolored bat |
|  | KJ641699 | Bat picornavirus | Eastern bent wing bat |
|  | KJ686308 | Enterovirus A71 | Human |
|  | KJ821021 | Porcine sapelovirus | Pig |
|  | KM269482 | Encephalomyocarditis virus | Chimpanzee |
|  | KM269483 | Equine rhinitis A virus | Camel |
|  | KM396707 | Lesavirus 1 | Ring tailed lemur |
|  | KM873613 | Tortoise picornavirus | African spurred tortoise |
|  | KM873616 | Tortoise picornavirus | Spur thighed tortoise |
|  | KM873617 | Tortoise picornavirus | Spider tortoise |
|  | KM892501 | Enterovirus D68 | Human |
|  | KT003716 | Foot and mouth disease virus | Water buffalo |
|  | KT229612 | Hepatovirus A | Woodchuck |
|  | KT452644 | Rodent hepatovirus | Gray dwarf hamster |
|  | KT452685 | Rodent hepatovirus | Jaliscan cotton rat |
|  | KT452735 | Rodent hepatovirus | Rusty bellied rat |
|  | KU291242 | Foot and mouth | Cow |
|  | KU359214 | Senecavirus | Mouse |
|  | KX035096 | Hepatovirus A | Human |
|  | LC006971 | Bovine picornavirus | Cow |
|  | LC113907 | Swine picornavirus | Pig |
|  | NC_003976 | Ljungan virus | Bank vole |
|  | NC_003982 | Equine rhinitis A virus | Horse |
|  | NC_004421 | Bovine kobuvirus | Cow |
|  | NC_009891 | Seal picornavirus type 1 | Ringed seal |
|  | NC_010354 | Bovine rhinitis B virus | Cow |
|  | NC_012802 | Human cosavirus D | Human |
|  | NC_014412 | Oscivirus A1 | Oriental Magpie Robin |
|  | NC_014413 | Oscivirus A2 | Grey backed Thrush |
|  | NC_015934 | Bat picornavirus 3 | Chinese rufous horseshoe bat |
|  | NC_015936 | Mouse kobuvirus | Canyon mouse |
|  | NC_015940 | Bat picornavirus 1 | Small bent winged bat |
|  | NC_015941 | Bat picornavirus 2 | Western bent winged bat |
|  | NC_016156 | Feline picornavirus | Cat |
|  | NC_016403 | Quail picornavirus | Quail |
|  | NC_018668 | Bovine hungarovirus | Cow |
|  | NC_022332 | Eel picornavirus 1 | European eel |
|  | NC_023422 | Caprine kobuvirus | Black goat |
|  | NC_023857 | Chicken megrivirus | Chicken |
|  | NC_023858 | Turkey megrivirus | Turkey |
|  | NC_023987 | Mosavirus A2 | European roller |
|  | NC_023988 | Tortoise rafivirus A | Forstens tortoise |
|  | NC_024073 | Enterovirus | Mandrill |
|  | NC_025890 | Tortoise picornavirus | Hermanns tortoise |
|  | NC_026314 | Rabovirus A | Brown rat |
|  | NC_026470 | African bat icavirus A | Giant roundleaf bat |
|  | NC_026921 | Falcovirus A1 | Common kestrel |
|  | NC_027214 | Ampivirus A1 | Common newt |
|  | NC_027818 | Phopivirus | Harbor seal |
|  | NC_028364 | Shrew hepatovirus | Common shrew |
|  | NC_028365 | Hedgehog hepatovirus | Hedgehog |
|  | NC_028479 | Rabbit picornavirus | Rabbit |
|  | NC_028981 | Tupaia hepatovirus A | Northern tree shrew |
|  | NC_029854 | Yak enterovirus | Yak |
|  | NC_029905 | Enterovirus | Rhesus macaque |
| *Polyomaviridae* | AB048582 | JC virus | Human |
|  | EF186666 | Murine pneumotropic virus | Mouse |
|  | EF579803 | Simian virus 40 | Rhesus macaque |
|  | EU711058 | WU virus | Human 0 |
|  | FJ392560 | Merkel cell polyomavirus | Human |
|  | FN356910 | Polyomavirus | Bornean orangutan |
|  | GU345044 | Polyomavirus | Atlantic canary |
|  | HQ681903 | Goose hemorrhagic polyomavirus | Goose |
|  | HQ681905 | Goose hemorrhagic polyomavirus | Mule duck |
|  | JQ412134 | Equine polyomavirus | Horse |
|  | JQ958888 | Polyomavirus 1 | Little yellow shouldered bat |
|  | JQ958890 | Polyomavirus 3 | Flat faced fruit eating bat |
|  | JX159986 | Polyomavirus | Crab eating macaque |
|  | JX416853 | Polyomavirus | Hamster |
|  | KC660158 | Finch polyomavirus | Gouldian finch |
|  | KF468310 | BK virus | Human |
|  | KF651951 | STL polyomavirus | Human |
|  | KJ577598 | Polyomavirus | Anubis baboon |
|  | KJ641707 | Bat polyomavirus | Beijing mouse eared bat |
|  | KJ641708 | Polyomavirus | Greater horseshoe bat |
|  | KM282376 | Polyomavirus 1 | Sea otter |
|  | KM496323 | Bovine polyomavirus 1 | Cow |
|  | KM496324 | Bovine polyomaviridae 2 | Cow |
|  | KM496326 | Bovine polyomaviridae 3 | Cow |
|  | KR065722 | Polyomavirus 1 | Pig |
|  | KR612374 | Polyomavirus | Common vole |
|  | KT184857 | Polyomavirus 1 | Bonobo |
|  | KT203767 | Budgerigar fledgling disease | Budgerigar |
|  | KT987218 | Polyomavirus 2 | Mouse |
|  | KU596573 | Human polyomavirus 6 | Human |
|  | KU746835 | KI polyomavirus | Human |
|  | KX509984 | Polyomavirus | Western red colobus |
|  | LN846619 | Polyomavirus 1 | Bark scorpion |
|  | NC_004763 | Polyomavirus | African green monkey |
|  | NC_007922 | Polyomavirus | Eurasian jackdaw |
|  | NC_009951 | Squirrel monkey polyomavirus | Squirrel monkey |
|  | NC_013796 | Polyomavirus 1 | California sealion |
|  | NC_014361 | Trichodysplasia spinulosa | Human |
|  | NC_014407 | Human polyomavirus 7 | Human |
|  | NC_014743 | Polyomavirus | Chimpanzee |
|  | NC_015150 | Polyomavirus 9 | Human |
|  | NC_018102 | MW polyomavirus | Human |
|  | NC_019850 | Polyomavirus 1 | Tana River red colobus |
|  | NC_019853 | Polyomavirus 1 | Red faced spider monkey |
|  | NC_019854 | Polyomavirus 1 | White fronted capuchin |
|  | NC_020065 | Polyomavirus 1 | Duke of Abruzzis free tailed bat |
|  | NC_020067 | Polyomavirus 1 | Heart nosed bat |
|  | NC_020068 | Polyomavirus 1 | Straw coloured fruit bat |
|  | NC_020071 | Polyomavirus 1 | Large eared free tailed bat |
|  | NC_020890 | Polyomavirus 12 | Human |
|  | NC_022519 | Polyomavirus 1 | African bush elephant |
|  | NC_023008 | Butcherbird polyomavirus | Grey butcherbird |
|  | NC_023845 | Polyomavirus | Raccoon |
|  | NC_024118 | New Jersey polyomavirus | Human |
|  | NC_025380 | Polyomavirus | Gorilla |
|  | NC_025790 | Polyomavirus 1 | Seabass |
|  | NC_025892 | Polyomavirus | Red eared guenon |
|  | NC_025894 | Polyomavirus 1 | Yellow baboon |
|  | NC_025895 | Mastomys polyomavirus | Natal multimammate mouse |
|  | NC_025898 | Polyomavirus 3 | Vervet monkey |
|  | NC_025899 | Polyomavirus 1 | Short beaked common dolphin |
|  | NC_026015 | Polyomavirus | Mexican freetailed bat |
|  | NC_026141 | Polyomavirus | Adelie penguin |
|  | NC_026244 | Polyomavirus 1 | Giant guitarfish |
|  | NC_026473 | Polyomavirus 1 | European badger |
|  | NC_026762 | Bat polyomavirus 6a | Sulawesi flying fox |
|  | NC_026767 | Bat polyomavirus 5b | Large flying fox |
|  | NC_026770 | Bat polyomavirus 6b | Moluccan naked backed fruit bat |
|  | NC_026942 | Polyomavirus 1 | Sheep |
|  | NC_026944 | Polyomavirus 1 | Emerald rockcod |
|  | NC_027531 | Polyomavirus 1 | Brown rat |
|  | NC_028117 | Polyomavirus | Bank vole |
|  | NC_028120 | Polyomavirus 1 | Sebas short tailed bat |
|  | NC_028121 | Polyomavirus 1 | Parnells mustached bat |
|  | NC_028122 | Polyomavirus 1 | Common vampire bat |
|  | NC_028123 | Polyomavirus 1 | Velvety free tailed bat |
|  | NC_028127 | Polyomavirus | Sumatran orangutan |
|  | NC_030838 | Polyomavirus 1 | Gilt head bream |
| *Potyviridae* | A34978 | MDMV | Maize |
|  | AB583211 | Papaya ringspot virus | Calabash |
|  | AB701693 | Turnip mosaic virus | Monkey orchid |
|  | AB701696 | Turnip mosaic virus | Hoary stock |
|  | AF103787 | Turnip mosaic virus | Mustard greens |
|  | AF103788 | Turnip mosaic virus | Chinese cabbage |
|  | AF506856 | Papaya ringspot virus | Pumpkin |
|  | AF506897 | Papaya ringspot virus | Cucumber |
|  | AF539410 | Turnip mosaic virus | Radish |
|  | AJ224623 | Yellow mosaic virus | Barley |
|  | AJ239038 | Yellow mosaic virus | Wheat |
|  | AJ297629 | Turnip mosaic virus | Turnip |
|  | AJ297630 | lettuce mosaic virus | Lettuce |
|  | AJ420020 | Zucchini yellow mosaic virus | Zucchini |
|  | AJ515379 | Sweet potato feathery mottle virus | Sweet potato |
|  | AJ628752 | Soybean mosaic virus | Crow dipper |
|  | AM184113 | Henbane mosaic virus | Chinese lantern |
|  | DQ925417 | Bean common mosaic virus strain Blackeye | Black bean |
|  | DQ925446 | Chilli veinal mottle virus | Chilli |
|  | DQ925462 | Turnip mosaic virus | Chinese mustard |
|  | EU586124 | Pepper mottle virus | Tomato |
|  | EU586134 | Pepper mottle virus | Bell pepper |
|  | HM348781 | Banana bract mosaic virus | Banana |
|  | JF427623 | Passion fruit woodiness virus | Passion fruit |
|  | JN692173 | Dasheen mosaic virus | Taro |
|  | JN863232 | Sunflower chlorotic mottle virus | Proboscis flower |
|  | JN863233 | Sunflower chlorotic mottle virus | Wild teasel |
|  | JX070153 | Pennisetum mosaic virus | Maize |
|  | JX417421 | Brown streak virus | Cassava |
|  | KF114860 | Bean common mosaic virus | Common bean |
|  | KF417755 | Lily mottle virus | Lily |
|  | KF463320 | Turnip mosaic virus | Oilseed rape |
|  | KF597285 | Leek yellow stripe virus | Garlic |
|  | KF862691 | yellow dwarf virus | Onion |
|  | KJ152154 | Hardenbergia mosaic virus | Hardenbergia comptoniana |
|  | KJ789129 | Telosma mosaic virus | Passion fruit |
|  | KJ789138 | Japanese yam mosaic virus | Chinese yam |
|  | KT936508 | Turnip mosaic virus | False Starwort |
|  | KU315175 | Moroccan watermelon mosaic virus | Marrow |
|  | KU355553 | Zucchini shoestring virus | Zucchini |
|  | L22907 | Watermelon mosaic virus | Watermelmon |
|  | L32960 | Peanut mottle virus | Peanut |
|  | LC114511 | Dasheen mosaic virus | Konjac |
|  | MSCCP | Mosaic virus | Sugarcane |
|  | NC_008558 | Blackberry virus Y | Blackberry |
|  | NC_014252 | Panax virus Y | Notoginseng |
|  | NC_018572 | Caladenia virus A | Pink fairy orchid |
|  | NC_019412 | Yam mild mosaic virus | Indian yam |
|  | NC_029051 | Jasmine ringspot virus | Jasmine |
|  | PTWCOPOL | Vein banding mosaic virus | Tobacco |
|  | PVYCPA | potato virus | Potato |
|  | X63630 | Tulip mosaic virus | Tulip |
|  | X66027 | Severe mosaic virus | Bell pepper |
|  | X67672 | Ringspot virus | Papaya |
|  | X76944 | G virus | Sweet potato |
|  | X96665 | Mosaic virus | Soybean |
|  | Y18634 | Cowpea aphid-borne mosaic virus | Cowpea |
|  | AB576080 | Plum pox virus | Plum |
| *Poxviridae* | AJ293568 | Yabalike | Human |
|  | AY386371 | Yaba monkey tumor virus | Vervet monkey |
|  | AY463007 | Dolphin poxvirus 1 | Indo Pacific bottlenose dolphin |
|  | AY780678 | Pinniped parapoxvirus | Spotted seal |
|  | AY952939 | Harbor seal parapoxvirus | Harbor seal |
|  | AY952948 | Steller sea lion parapoxvirus | Steller sea lion |
|  | DQ011153 | Monkeypox virus | Prairie dog |
|  | DQ066527 | Taterapox virus | Gerbil |
|  | DQ066528 | Cowpox | Human |
|  | DQ071862 | Cetacean poxvirus 1 | Rough toothed dolphin |
|  | DQ792504 | Horsepox virus | Horse |
|  | FJ807738 | Volepox | Vole |
|  | FJ807747 | Raccoonpox | Raccoon |
|  | FJ807756 | Skunkpox | Skunk |
|  | HF679132 | Entomopoxvirus | Moth |
|  | JN112370 | Cervidpoxvirus | Gazelle |
|  | KC017850 | Avipox | Chicken |
|  | KC017851 | Avipox | Blue eared pheasant |
|  | KC017852 | Avipox | Eastern imperial eagle |
|  | KC017855 | Avipox | Great bustard |
|  | KC017857 | Avipox | Indian peafowl |
|  | KC017866 | Avipox | Turkey |
|  | KC017868 | Avipox | Rock dove |
|  | KC017886 | Avipox | Oriental turtle dove |
|  | KC017888 | Avipox | Eurasian eagle owl |
|  | KC017891 | Avipox | Booted eagle |
|  | KC017892 | Avipox | Red legged partridge |
|  | KC017898 | Avipox | Southern giant petrel |
|  | KC017899 | Avipox | Pelagic cormorant |
|  | KC017905 | Avipox | Magellanic penguin |
|  | KC017934 | Avipox | Bald eagle |
|  | KC409037 | Cetacean poxvirus 1 | Striped dolphin |
|  | KC660085 | Myxoma virus | Tapeti |
|  | KF425535 | Sea otter poxvirus | Sea otter |
|  | KJ642619 | Monkeypox virus | Chimpanzee |
|  | KJ801920 | Pigeon pox | Pigeon |
|  | KM595078 | Cotia virus | Mouse |
|  | KP010356 | Orfvirus | Sheep |
|  | KT013210 | Vaccinia virus | Human |
|  | MOCDPOL | Molluscum contagiosum virus | Human |
|  | NC_001266 | Rabbit fibroma virus | Rabbit |
|  | NC_001993 | Entomopoxvirus | Grasshopper |
|  | NC_002520 | Entomopoxvirus | Moth |
|  | NC_003027 | Lumpy skin disease virus | Cow |
|  | NC_003310 | Monkeypox virus | Human |
|  | NC_003389 | Swinepox | Pig |
|  | NC_003391 | Camelpox | Camel |
|  | NC_004002 | Sheeppox | Sheep |
|  | NC_004003 | Goatpox | Goat |
|  | NC_004105 | Ectromelia virus | Mouse |
|  | NC_005309 | Canary pox | Canary |
|  | NC_005336 | Orfvirus | Goat |
|  | NC_005337 | Bovine papular stomatitis | Cow |
|  | NC_006966 | Deerpox | Muledeer |
|  | NC_008030 | Crocodile pox | Nile crocodile |
|  | NC_009888 | Tanapox | Human |
|  | NC_016924 | Cotia virus SPAn232 | Mouse |
|  | NC_021249 | Entomopoxvirus | Leaf roller |
|  | NC_022563 | Squirrelpox | Red squirrel |
|  | NC_025963 | Parapoxvirus | Red deer |
|  | NC_030656 | Pteropox virus | Little red flying fox |
|  | VARCG | Variola major virus | Human |
| *Reoviridae* | AB792653 | Rotavirus A | Cat |
|  | AB971760 | Rotavirus A | Sugar glider |
|  | AF133429 | Kadipiro virus | Mosquito |
|  | AF133431 | St Croix river virus | Tick |
|  | AF168005 | Banna virus | Human |
|  | AF368033 | Ndelle virus | House mouse |
|  | AF418295 | Chum salmon reovirus | Salmon |
|  | AY317099 | Liao ning virus | Mosquito |
|  | DQ664184 | Mammalian orthoreovirus | Human |
|  | EU636924 | Rotavirus A | Rhesus monkey |
|  | FJ347100 | Rotavirus A | Guanaco |
|  | GQ428141 | Rotavirus A giraffe | Giraffe |
|  | GQ468266 | Mammalian orthoreovirus | Masked palm civet |
|  | HM222980 | Avian orthoreovirus | Steller sea lion |
|  | HM543475 | Lipovnik virus | Human |
|  | HM543478 | Tribec virus | Bank vole |
|  | HM989931 | Reovirus | Turbot |
|  | HQ414127 | Reovirus | Mud crab |
|  | HQ630922 | Equine encephalosis | Horse |
|  | HQ642769 | Mammalian orthoreovirus | Pig |
|  | HQ896710 | Rotavirus C | Human |
|  | JQ070366 | Tilligerry virus | Cow |
|  | JQ070376 | Eubenangee virus | Cow |
|  | JX204811 | Rotavirus A | Pheasant |
|  | JX204822 | Rotavirus A | Turkey |
|  | JX478251 | Duck reovirus | Duck |
|  | JX947843 | Banna | Freshwater carp |
|  | KC288130 | Kemerovo virus | Common redstart |
|  | KC432629 | Sathuvachari virus | Brahminy starling |
|  | KC462149 | Mammalian orthoreovirus | Mink |
|  | KC588357 | Cypovirus 5 | Moth |
|  | KC669539 | Heramatsu virus | Eastern long fingered bat |
|  | KC853042 | Corriparta virus | Horse |
|  | KF017509 | Bluetongue virus | Sheep |
|  | KF296322 | Mobuck virus | White tail deer |
|  | KF664133 | Bluetongue virus 16 | Goat |
|  | KF712476 | Reovirus | Grass carp |
|  | KF746187 | Tibet orbivirus | Mosquito |
|  | KF791261 | Mammalian orthoreovirus | Least horseshoe bat |
|  | KJ191550 | Baku virus | Seagull |
|  | KJ476700 | Avian orthoreovirus | Chicken |
|  | KJ477127 | Murine Rotavirus | Mouse |
|  | KJ495745 | Wallal virus | Kangaroo |
|  | KJ495755 | Warrego virus | Kangaroo |
|  | KJ676379 | Mammalian orthoreovirus | Cow |
|  | KJ740726 | Reovirus | Largemouth bass |
|  | KJ746836 | Changuinola virus | Rice rat |
|  | KJ865902 | Avian orthoreovirus | Turkey |
|  | KJ950937 | Mulberry orbivirus | Brown rat |
|  | KM099522 | Bluetongue virus | Cow |
|  | KM214456 | Bat rotavirus | Mediterranean horseshoe bat |
|  | KM391741 | Epizootic hemorrhagic disease virus | Cow |
|  | KP006506 | Rotavirus A | Human |
|  | KP196603 | Bluetongue virus | Alpaca |
|  | KP638402 | Reovirus | Chinese mitten crab |
|  | KP988013 | Rotavirus C | Dog |
|  | KR052714 | Rotavirus B | Wild boar |
|  | KR349187 | Mangshi virus | Mosquito |
|  | KR476800 | Avian orthoreovirus | Partridge |
|  | KT030360 | African horse sickness virus | Horse |
|  | KT444522 | Mammalian orthoreovirus | Ricketts bigfooted bat |
|  | KT444562 | Mammalian orthoreovirus | Great roundleaf bat |
|  | KT873808 | Rotavirus A | Red fox |
|  | KT934648 | Avian rotavirus A | Spotted dove |
|  | KT962027 | Rotavirus | Pig |
|  | KU140720 | Epizootic hemorrhagic disease virus | White tail deer |
|  | KU311708 | Reovirus | Chesapeake blue crab |
|  | KU708259 | Rotavirus A | Roe deer |
|  | KX268765 | Rotavirus A | Straw coloured fruit bat |
|  | LC088096 | Rotavirus A | Velvet scoter |
|  | NC_003696 | Eyach virus | Human |
|  | NC_004181 | Colorado tick fever virus | Human |
|  | NC_005167 | Reovirus | Golden shiner |
|  | NC_007656 | Yunnan orbivirus | Mosquito |
|  | NC_007667 | Aedes pseudoscutellaris reovirus | Mosquito |
|  | NC_012754 | Stretch Lagoon orbivirus | Mosquito |
|  | NC_014522 | Great Island virus | Common guillemot |
|  | NC_015878 | orthoreovirus | Baboon |
|  | NC_021541 | Rotavirus B | Human |
|  | NC_025486 | Fako virus | Mosquito |
|  | NC_027553 | Chobar gorge virus | Cow |
|  | NC_027803 | Fengkai orbivirus | Mosquito |
| *Retroviridae* | AB559882 | RD114 retrovirus | Cat |
|  | AF014793 | Hyperplasia | Walleye |
|  | AF016316 | Equine infectious anemia | Horse |
|  | AF075269 | Simian immunodeficiency | LHoest monkey |
|  | AF201902 | Equine foamy | Horse |
|  | AF334679 | Simian immunodeficiency | Sooty mangabey |
|  | AF411814 | Amphotropic murine leukemia | Mouse |
|  | AJ225236 | RV Tuatara | Turatara |
|  | AJ236119 | Retrovirus | Short beaked echidna |
|  | AJ236126 | Retrovirus | Grey partridge |
|  | AJ236134 | Retrovirus | Eurasian wren |
|  | AJ292966 | Avian endogenous retrovirus | Grey junglefowl |
|  | AJ544579 | Simian foamy | Bornean orangutan |
|  | AJ627546 | Simian foamy | Sumatran orangutan |
|  | AM745105 | Simian immunodeficiency | Western red colobus |
|  | AY101611 | Visna | Goat |
|  | AY221515 | Simian immunodeficiency | Southern pigtailed macaque |
|  | AY340701 | Simian immunodeficiency | Mona monkey |
|  | AY518534 | Simian immunodeficiency | Black mangabey |
|  | AY523866 | Simian immunodeficiency | De Brazzas monkey |
|  | AY523867 | Simian immunodeficiency | Sykes monkey |
|  | AY590142 | Simian T lymphotropic 1 | Stump tailed macaque |
|  | AY655744 | Simian immunodeficiency | Gabon talapoin |
|  | AY713445 | Feline immunodeficiency | Pallass cat |
|  | AY878194 | Feline immunodeficiency | Ocelot |
|  | AY878195 | Feline immunodeficiency | Jaguarundi |
|  | AY878242 | Feline immunodeficiency | Snow leopard |
|  | BLVGPE | Bovine leukimia | Cow |
|  | DQ192583 | Feline immunodeficiency | Cougar |
|  | DQ201173 | Simian immunodeficiency | Rhesus macaque |
|  | EU010385 | Simian foamy | Spider monkey |
|  | EU117992 | Feline immunodeficiency | Lion |
|  | FJ006946 | Simian foamy | Ugandan red colobus |
|  | FJ402534 | Simian immunodeficiency | Pigtailed macaque |
|  | FJ424871 | Simian immunodeficiency | Gorilla |
|  | FJ957880 | Simian T lymphotropic | Greater spot nosed monkey |
|  | FM165200 | Simian immunodeficiency | Olive colobus |
|  | FN859999 | Simian foamy | Western red colobus |
|  | GQ381130 | Caprine arthritis encephalitis | Goat |
|  | GU356395 | Simian foamy | Common marmoset |
|  | GU980187 | Porcine endogenous retrovirus | Pig |
|  | HIV2D194 | Human immunodeficiency 2 | Human |
|  | HIVMCK1 | Human immunodeficiency 1 | Human |
|  | HM210570 | Small ruminant lentivirus | Goat |
|  | HM245790 | Simian foamy | Gorilla |
|  | HQ378594 | Simian immunodeficiency | African green monkey |
|  | HQ450608 | Simian foamy | Mandrill |
|  | HQ450613 | Simian foamy | Grey cheeked mangabey |
|  | HQ450614 | Simian foamy | Sun tailed monkey |
|  | HQ450615 | Simian foamy | Collared mangabey |
|  | JDVCG | Jembrana disease | Banteng |
|  | JF502417 | Caprine arthritis encephalitis | Sheep |
|  | JF810903 | Simian foamy | Sooty mangabey |
|  | JN801175 | Simian foamy | Rhesus macaque |
|  | JQ292910 | Retrovirus | Blackflyingfox |
|  | JQ292912 | Retrovirus | Ricketts big footed bat |
|  | JQ292914 | Retrovirus | Pearsons horseshoe bat |
|  | JQ303225 | Retrovirus | Greater horseshoe bat |
|  | JQ867466 | Simian foamy | Greater spot nosed monkey |
|  | JQ951956 | Retrovirus | Greater false vampire bat |
|  | JQ951958 | Retrovirus | Leschenaults rousette |
|  | JX178450 | Simian immunodeficiency | Chimpanzee |
|  | JX848322 | Avian leukosis | Green winged Teal |
|  | KC189895 | Nasal tumor | Sheep |
|  | KC331072 | Simian foamy | Venezuelan red howler |
|  | KC331076 | Simian foamy | Red handed howler |
|  | KC331079 | Simian foamy | Tufted capuchin |
|  | KC331082 | Simian foamy | Brown howler |
|  | KC845928 | Retrovirus | Tasmanian devil |
|  | KF026286 | Simian foamy | Taiwanese macaque |
|  | KF572484 | Retrovirus | Grassland mosaic tailed rat |
|  | KF906156 | Feline immunodeficiency | Bobcat |
|  | KF906169 | Feline immunodeficiency | Mountain lion |
|  | KF906194 | Feline immunodeficiency | Puma |
|  | KJ461716 | Simian immunodeficiency | Red tailed guenon |
|  | KJ668270 | Ecotropic murine leukemia | Mouse |
|  | KM233624 | Simian foamy | Northern greater galago |
|  | KM378566 | Simian immunodeficiency | Drill |
|  | KP143760 | Simian foamy | Golden bellied capuchin |
|  | KP284572 | Avian leukosis | Chicken |
|  | KP691837 | Jaagsiekte sheep retrovirus | Sheep |
|  | KR528438 | Simian foamy | Golden backed uakari |
|  | KR528446 | Simian foamy | Golden lion tamarin |
|  | KR902440 | Simian foamy | Black headed spider monkey |
|  | KR902443 | Simian foamy | Geoffroys spider monkey |
|  | KR902452 | Simian foamy | Long haired spider monkey |
|  | KR902480 | Simian foamy | Brown woolly monkey |
|  | KR902481 | Simian foamy | White faced saki |
|  | KR902487 | Simian foamy | Black capped squirrel monkey |
|  | KT724050 | Leukemia | Gibbon |
|  | KU705348 | Feline immunodeficiency | Leaopard |
|  | LC114462 | Simian immunodeficiency | Malbrouck |
|  | LM999945 | Simian immunodeficiency | Tantalus monkey |
|  | M30931 | Simian immunodeficiency | Grivet |
|  | MLFCG | Friend murine leukemia | Mouse |
|  | MLMCG | Moloney murine leukemia | House mouse |
|  | NC_001413 | Bovine immunodeficiency like | Cow |
|  | NC_001514 | Retrovirus | Squirrel monkey |
|  | NC_001550 | Mason Pfizer monkey | Rhesus macaque |
|  | NC_001815 | simian T lymphotropic 2 | Human |
|  | NC_001831 | Bovine foamy | Cow |
|  | NC_010819 | Simian foamy | Macaque |
|  | NC_010820 | Simian foamy | African green monkey |
|  | NC_010955 | Retrovirus | African clawed frog |
|  | RMU94692 | Rauscher murine leukemia | Mouse |
|  | S80082 | Murine AIDS related provirus | House mouse |
|  | SIVMNDGB1 | Simian immunodeficiency | Mandrill |
|  | U04327 | Simian foamy | Chimpanzee |
|  | X57002 | Feline immunodeficiency | Cat |
|  | X68524 | Rous sarcoma | Duck |
|  | Y07724 | Human foamy | Human |
| *Rhabdoviridae* | AB490792 | Viral hemorrhagic septicemia virus | Japanese flounder |
|  | AB519642 | Rabies virus | Common vampire bat |
|  | AB609604 | Nishimuro virus | Wild boar |
|  | AB618036 | Rabies virus | Sheep |
|  | AB635373 | Rabies virus | Golden palm civet |
|  | AB645847 | Rabies virus | Groundhog |
|  | AY450644 | Rhabdovirus | Starry flounder |
|  | EF614259 | Aravan virus | Lesser mouse eared bat |
|  | EF614260 | Irkur virus | Greater tube nosed bat |
|  | EF614261 | Khujand virus | Whiskered bat |
|  | EU259198 | Lagos bat virus | Straw coloured fruit bat |
|  | EU293116 | Rabies virus | Mexican freetailed bat |
|  | EU293117 | Mokola virus | Shrew |
|  | FJ376982 | Hirame rhabdovirus | Stone flounder |
|  | FJ866835 | Rabies virus | Dog |
|  | FJ905105 | Lyssavirus Ozernoe | Human |
|  | FJ952155 | Durham virus | American coot |
|  | FN665788 | Viral hemorrhagic septicemia virus | Brown trout |
|  | GU170202 | Lagos bat virus | Egyptian fruit bat |
|  | HQ003891 | Scophthalmus maximus rhabdovirus | Turbot fish |
|  | JF911700 | Yug bogdanovac virus | Sandfly |
|  | JN935380 | Adelaide River virus | Cow |
|  | JN986749 | Duvenhage virus | Human |
|  | JQ595377 | Rabies virus | Yuma myotis |
|  | JQ595378 | Rabies virus | Townsends big eared bat |
|  | JQ647510 | Rabies virus | Donkey |
|  | JQ685893 | Rabies virus | Striped skunk |
|  | JQ685895 | Rabies virus | Silver haired bat |
|  | JQ685899 | Rabies virus | Gray fox |
|  | JQ685919 | Rabies virus | Eastern red bat |
|  | JQ685921 | Rabies virus | Southeastern myotis |
|  | JQ685922 | Rabies virus | Eastern pipistrelle |
|  | JQ685925 | Rabies virus | Big brown bat |
|  | JQ685929 | Rabies virus | Spotted skunk |
|  | JQ685937 | Rabies virus | Ring tailed cat |
|  | JQ685953 | Rabies virus | Human |
|  | JQ685963 | Rabies virus | White nosed coati |
|  | JQ685973 | Rabies virus | Coyote |
|  | JX297815 | Bas congo virus | Human |
|  | JX473840 | Rabies virus | Greater kudu |
|  | JX827265 | Eel virus | European eel |
|  | KC171645 | Rabies virus | Raccoon |
|  | KC676792 | Fikirini rhabdovirus | Commersons leaf nosed bat |
|  | KC685626 | Paralichthys olivaceus rhabdovirus | Olive flounder |
|  | KC762941 | Rabies virus | Chinese ferret badger |
|  | KC778774 | Viral hemorrhagic septicemia virus | Rainbow trout |
|  | KC994644 | Arboretum virus | Psorophora mosquito |
|  | KF155007 | Mokola virus | Cat |
|  | KF360973 | North creek virus | Culex mosquito |
|  | KF395226 | Sunguru virus | Chicken |
|  | KF534749 | Puerto almendras virus | Ochlerotatus mosquito |
|  | KJ396935 | Long Island tick rhabdovirus | Tick |
|  | KJ564280 | Rabies virus | Fallow deer |
|  | KJ830812 | Zahedan rhabdovirus | Tick |
|  | KM012169 | New Jersey virus | Horse |
|  | KM204982 | Garba virus | Malachite kingfisher |
|  | KM204983 | Barur virus | Black rat |
|  | KM204985 | Kwatta virus | Culex mosquito |
|  | KM204990 | Muir springs virus | Aedes mosquito |
|  | KM204998 | Oita virus | Little Japanese horseshoe bat |
|  | KM204999 | Klamath virus | Montane vole |
|  | KM205000 | Chaco virus | Giant ameiva |
|  | KM205003 | Harlingen virus | Culex mosquito |
|  | KM205006 | Dantec virus | Human |
|  | KM205009 | New minto virus | Tick |
|  | KM205010 | Landjia virus | Brown throated martin |
|  | KM205013 | Sawgrass virus | Tick |
|  | KM205018 | Bahia Grande virus | Aedes mosquito |
|  | KM205020 | Connecticut virus | Tick |
|  | KM205021 | Keuraliba virus | Kemps gerbil |
|  | KM205024 | Radi virus | Phlebotomus mosquito |
|  | KM205026 | Mount elgon virus | Eloquent horseshoe bat |
|  | KM244767 | Viral hemorrhagic septicemia virus | Rockling |
|  | KM276084 | BEFV | Cow |
|  | KM594024 | Rabies virus | Common marmoset |
|  | KM594034 | Rabies virus | Broad eared bat |
|  | KM594039 | Rabies virus | Crab eating fox |
|  | KP723638 | Rabies virus | Ethiopian wolf |
|  | KR822811 | Sigmavirus | Drosophila |
|  | KR822826 | Pararge aegeria rhabdovirus | Speckled wood |
|  | NC_000903 | Snakehead virus | Common snakehead |
|  | NC_002803 | Spring viraemia | Common carp |
|  | NC_007020 | Tupaia virus | Tree shrew |
|  | NC_008514 | Siniperca chuatsi rhabdovirus | Mandarin fish |
|  | NC_009527 | European bat lyssavirus 1 | Serotine bat |
|  | NC_009528 | European bat lyssavirus 2 | Human |
|  | NC_017685 | Obodhiang virus | Aedes mosquito |
|  | NC_017714 | Kotonkan virus | Cow |
|  | NC_018629 | Ikoma lyssavirus | African civet |
|  | NC_020803 | Perch rhabdovirus | European perch |
|  | NC_020806 | Isfahan virus | Human |
|  | NC_025251 | Bokeloh bat lyssavirus | Natterers bat |
|  | NC_025356 | Pike fry rhabdovirus | Pike fry |
|  | NC_025358 | Berrimah virus | Cow |
|  | NC_025359 | Moussa virus | Culex mosquito |
|  | NC_025364 | Malpais Spring virus | Mule deer |
|  | NC_025365 | Shimoni bat virus | Commersons leaf nosed bat |
|  | NC_025371 | Tench rhabdovirus | Tench |
|  | NC_025376 | Grass carp rhabdovirus V76 | Grass carp |
|  | NC_025377 | Fruit bat virus | Fruit bat |
|  | NC_025382 | Spodoptera frugiperda rhabdovirus | Fall armyworm |
|  | NC_025396 | Kimberley virus | Cow |
|  | NC_025397 | Coastal Plains virus | Cow |
|  | NC_028239 | Koolpinyah virus | Cow |
|  | NC_028867 | Fox fecal rhabdovirus | Red fox |
|  | NC_030451 | Rhabdovirus | Parasitoid wasp |
| *Togaviridae* | AB032553 | Sagiyama virus | Horse |
|  | AB859822 | Getah virus | Wild boar |
|  | AF079457 | Igbo Ora Virus | Human |
|  | AY604238 | Alphavirus | Norwegian salmon |
|  | BFU73745 | Barmah Forest | Human |
|  | DQ149204 | Salmon pancreas disease virus | Atlantic salmon |
|  | EU350586 | Semliki forest virus | Human |
|  | FJ445511 | Chikungunya virus | Human |
|  | GQ227789 | Highlands J virus | Blue jay |
|  | GQ433354 | Ross river virus | Human |
|  | GQ433356 | Ross river virus | Masked finch |
|  | GQ433357 | Ross river virus | Agile wallaby |
|  | GQ433360 | Ross river virus | Magpie lark |
|  | GU167952 | Highlands J virus | Barred owl |
|  | HM045821 | Chikungunya virus | Bat |
|  | HM147984 | Babanki virus | Brown rat |
|  | HM147985 | Bebaru virus | Human |
|  | HM147986 | Buggy Creek virus | House sparrow |
|  | HM147991 | Trocara virus | Culex mosquito |
|  | HM147992 | Una virus | Human 0 |
|  | HM147993 | Whataroa virus | Song thrush |
|  | HM210093 | Eastern equine encephalitis virus | Pheasant |
|  | JN989957 | Ndumu virus | Pig |
|  | JQ799139 | Salmonid alphavirus subtype 3 | Atlantic salmon |
|  | JX570540 | Sindbis virus | Hooded crow |
|  | KC344509 | Venezuelan equine encephalitis virus | Golden hamster |
|  | KC344528 | Venezuelan equine encephalitis virus | Human |
|  | KF283988 | Semliki Forest virus | Goat |
|  | KF680222 | Middelburg virus | Horse |
|  | KJ409555 | Highlands J virus | Mississippi Sandhill Crane |
|  | KJ469579 | Eastern equine encephalitis virus | Dog |
|  | KJ469599 | Eastern equine encephalitis virus | Red eyed vireo |
|  | KJ469623 | Madariaga virus | Golden hamster |
|  | KJ554965 | Western equine encephalitis virus | Horse |
|  | KJ554969 | Western equine encephalitis virus | White crowned sparrow |
|  | KJ554970 | Western equine encephalitis virus | Western gray squirrel |
|  | KJ554971 | Western equine encephalitis virus | Texas tortoise |
|  | KM400591 | Mayaro virus | Human |
|  | KM923920 | Chikungunya virus | Crab eating macaque |
|  | KR132531 | Madariaga virus | Horse |
|  | KR260737 | Venezuelan equine encephalitis virus | Horse |
|  | KT121724 | Sindbis virus | Chinese hamster |
|  | KT121726 | Sindbis virus | Golden hamster |
|  | KT429026 | Highlands J virus | Rock Partridge |
|  | KT754168 | Mayaro virus | Common marmoset |
|  | KU840310 | Eastern equine encephalitis virus | Northern mockingbird |
|  | KU840320 | Eastern equine encephalitis virus | Morning dove |
|  | KU840341 | Eastern equine encephalitis virus | Grey crowned crane |
|  | KU840349 | Eastern equine encephalitis virus | Yellow rumped warbler |
|  | KU840372 | Eastern equine encephalitis virus | Whooping crane |
|  | KU840373 | Eastern equine encephalitis virus | Blue jay |
|  | KU840374 | Eastern equine encephalitis virus | Deer |
|  | KU840375 | Eastern equine encephalitis virus | Common grackle |
|  | KU840377 | Eastern equine encephalitis virus | Crane |
|  | KU840378 | Eastern equine encephalitis virus | Emu |
|  | KU840379 | Eastern equine encephalitis virus | Finch |
|  | KX000078 | Eastern equine encephalitis virus | Whitethroated sparrow |
|  | KX000079 | Eastern equine encephalitis virus | Red winged blackbird |
|  | KX000086 | Eastern equine encephalitis virus | Gray catbird |
|  | KX000099 | Eastern equine encephalitis virus | Ring necked pheasant |
|  | KX000102 | Eastern equine encephalitis virus | Swainsons thrush |
|  | KX000141 | Eastern equine encephalitis virus | American crow |
|  | KX000153 | Eastern equine encephalitis virus | Indigo bunting |
|  | KX000163 | Eastern equine encephalitis virus | Common yellowthroat |
|  | KX000164 | Eastern equine encephalitis virus | Human |
|  | KX000169 | Eastern equine encephalitis virus | White eyed vireo |
|  | KX000182 | Eastern equine encephalitis virus | Bald eagle |
|  | KX000225 | Eastern equine encephalitis virus | Alder flycatcher |
|  | KX000230 | Eastern equine encephalitis virus | Horse |
|  | LC079089 | Getah virus | Horse |
|  | NC_001512 | Onyong nyong virus | Human |
|  | NC_001544 | Ross river virus | Mouse |
|  | NC_003433 | Sleeping disease virus | Rainbow trout |
|  | NC_003900 | Aura virus | Culex mosquito |
|  | NC_012561 | Highlands J virus | Redtailed hawk |
|  | NC_013528 | Fort Morgan virus | Cliff swallows |
|  | NC_016960 | Southern elephant seal virus | Southern elephant seal |
|  | NC_018615 | Eilat virus | Anopheles mosquito |
|  | NC_023812 | Madariaga virus | Tufted capuchin |
