# Supplementary material for: Comparative analysis estimates the relative frequencies of co-divergence and cross-species transmission within viral families
Source: PLoS Pathog. 2017 Feb 8;13(2):e1006215. doi: 10.1371/journal.ppat.1006215 (PMC5319820; doi:10.1371/journal.ppat.1006215)
Supplement: S2 Table — Virus genera were excluded either due to lack of available data or because we were unable to obtain a reliable alignment of sufficient length for phylogenetic analysis (i.e. at least 100 amino acids after trimAl pruning). (DOCX) [file ppat.1006215.s004.docx]

**Supplementary Table 2.** Summary of the virus and host diversity included and excluded in this study. Virus genera were excluded either due to lack of available data or that they were unable to be aligned with accuracy.

| Virus  Family | Genome  Type | Virus Genera Included | Virus Genera Excluded | Host Classes Included |
| --- | --- | --- | --- | --- |
| *Adenoviridae* | DNA | Atadenovirus  Aviadenovirus  Mastadenovirus  Siadenovirus | Ichtadenovirus | Reptiles / amphibians  Birds  Mammals |
| *Bunyaviridae* | RNA | Hantavirus  Nairovirus  Orthobunyavirus  Phlebovirus | Tospovirus | Invertebrates  Birds  Mammals |
| *Caliciviridae* | RNA | Lagovirus  Nebovirus  Norovirus  Sapovirus  Vesivirus | -- | Fish  Reptiles / amphibians  Birds  Mammals |
| *Coronaviridae* | RNA | Alphacoronavirus  Betacoronavirus  Deltacoronavirus  Gammacoronavirus  Bafinivirus  Torovirus | -- | Fish  Birds  Mammals |
| *Flaviviridae* | RNA | Hepacivirus  Flavivirus  Pegivirus  Pestivirus | -- | Invertebrates  Birds  Mammals |
| *Hepadnaviridae* | DNA | Orthohepadnavirus  Avihepadnavirus | -- | Fish  Reptiles / amphibians  Birds  Mammals |
| *Herpesviridae* | DNA | Iltovirus  Proboscivirus  Cytomegalovirus  Mardivirus  Rhadinovirus  Macavirus  Roseolovirus  Simplexvirus  Scutavirus  Varicellovirus  Percavirus  Lymphocryptovirus  Muromegalovirus | -- | Reptiles / amphibians  Birds  Mammals |
| *Orthomyxoviridae* | RNA | Influenza virus A  Influenza virus B  Influenza virus C  Influenza virus D  Isavirus  Thogotovirus  Quaranjavirus | -- | Invertebrates  Fish  Birds  Mammals |
| *Papillomaviridae* | DNA | Dyoxipapillomavirus  Omikronpapillomavirus  Dyodeltapapillomavirus  Omegapapillomavirus  Nupapillomavirus  Dyomupapillomavirus  Dyozetapapillomavirus  Kappapapillomavirus  Upsilonpapillomavirus  Dyoetapapillomavirus  Sigmapapillomavirus  Lambdapapillomavirus  Taupapillomavirus  Betapapillomavirus  Xipapillomavirus  Dyoepsilonpapillomavirus  Thetapapillomavirus  Etapapillomavirus  Rhopapillomavirus  Dyothetapapillomavirus  Dyoomikronpapillomavirus  Gammapapillomavirus  Alphapapillomavirus  Zetapapillomavirus  Deltapapillomavirus  Dyolambdapapillomavirus  Dyosigmapapillomavirus  Dyorhopapillomavirus  Psipapillomavirus  Dyokappapapillomavirus  Pipapillomavirus  Iotapapillomavirus  Epsilonpapillomavirus  Phipapillomavirus  Dyonupapillomavirus  Dyopipapillomavirus  Dyoiotapapillomavirus  Mupapillomavirus | -- | Fish  Reptiles / amphibians  Birds  Mammals |
| *Paramyxoviridae* | RNA | Aquaparamyxovirus  Avulavirus  Ferlavirus  Henipavirus  Morbillivirus  Respirovirus  Rubulavirus | -- | Fish  Reptiles / amphibians  Birds  Mammals |
| *Parvoviridae* | DNA | Ambidensovirus  Iteradensovirus  Amdoparvovirus  Aveparvovirus  Bocaparvovirus  Dependoparvovirus  Erythroparvovirus  Protoparvovirus  Tetraparvovirus | Brevidensovirus  Hepandensovirus  Penstyldensovirus  Copiparvovirus | Invertebrates  Reptiles / amphibians  Birds  Mammals |
| *Picornaviridae* | RNA | Aphthovirus  Aquamavirus  Avihepatovirus  Cardiovirus  Cosavirus  Enterovirus  Hepatovirus  Kobuvirus  Megrivirus  Parechovirus  Piscevirus  Salivirus  Sapelovirus  Senecavirus  Teschovirus | Avisivirus  Dicipivirus  Erbovirus  Tremovirus | Fish  Reptiles / amphibians  Birds  Mammals |
| *Polyomaviridae* | DNA | Alphapolyomavirus  Betapolyomavirus  Gammapolyomavirus  Deltapolyomavirus | -- | Invertebrates  Fish  Birds  Mammals |
| *Potyviridae* | RNA | Brambyvirus  Bymovirus  Ipomovirus  Macluravirus  Poacevirus  Potyvirus | Rymovirus  Tritimovirus | Plants |
| *Poxviridae* | DNA | Betaentomopoxvirus  Yatapoxvirus  Cervidpoxvirus  Leporipoxvirus  Suipoxvirus  Molluscipoxvirus  Crocodylidpoxvirus  Alphaentomopoxvirus  Capripoxvirus  Orthopoxvirus  Avipoxvirus  Parapoxvirus | Gammaentomopoxvirus | Invertebrates  Reptiles / amphibians  Birds  Mammals |
| *Reoviridae* | RNA | Aquareovirus  Rotavirus  Seadornavirus  Coltivirus  Orbivirus  Cypovirus  Orthoreovirus  Dinovernavirus  Cardoreovirus | Fijivirus  Phytoreovirus  Mimoreovirus  Idnoreovirus  Oryzavirus  Mycoreovirus | Invertebrates  Fish  Birds  Mammals |
| *Retroviridae* | RNA | Alpharetrovirus  Betaretrovirus  Deltaretrovirus  Epsilonretrovirus  Gammaretrovirus  Lentivirus | -- | Fish  Reptiles / amphibians  Birds  Mammals |
| *Rhabdoviridae* | RNA | Ephemerovirus  Lyssavirus  Novirhabdovirus  Perhabdovirus  Sigmavirus  Sprivivirus  Tibrovirus  Tupavirus  Vesiculovirus | Cytorhabdovirus  Dichorhavirus  Nucleorhabdovirus  Varicosavirus | Invertebrates  Fish  Reptiles / amphibians  Birds  Mammals |
| *Togaviridae* | RNA | Alphavirus | Rubivirus | Invertebrates  Fish  Reptiles / amphibians  Birds  Mammals |
