# Supplementary material for: Comparative analysis estimates the relative frequencies of co-divergence and cross-species transmission within viral families
Source: PLoS Pathog. 2017 Feb 8;13(2):e1006215. doi: 10.1371/journal.ppat.1006215 (PMC5319820; doi:10.1371/journal.ppat.1006215)
Supplement: S3 Table — The overall nPH85 distances are illustrated in Fig 2 in the main text. (DOCX) [file ppat.1006215.s005.docx]

**Supplementary Table 3.** Overall nPH85 distances, means and 95% percentiles between two unrooted phylogenetic trees for each virus family by normalizing the [Penny and Hendy (14](#_ENREF_14)) tree topology distance method, implemented in in NELSI v0.1 ([40](#_ENREF_40)). The overall nPH85 distances are illustrated in Fig. 2 in the main text.

| **Virus Family** | **Standardized Topology Distance** | **Mean Standardized Topology Distance** | **Lower 95%** | **Upper 95%** |
| --- | --- | --- | --- | --- |
| *Adenoviridae* | 0.875 | 0.953541666 | 0.9265625 | 0.9583333 |
| *Bunyaviridae* | 0.9694656 | 0.992824427 | 0.969465649 | 1 |
| *Caliciviridae* | 0.8924731 | 0.892473118 | 0.892473118 | 0.892473118 |
| *Coronaviridae* | 0.90625 | 0.9315625 | 0.921875 | 0.9375 |
| *Flaviviridae* | 0.9665272 | 0.965188285 | 0.958158996 | 0.966527197 |
| *Hepadnaviridae* | 0.6 | 0.9905 | 0.95 | 1 |
| *Herpesviridae* | 0.9349593 | 0.963252033 | 0.951219512 | 0.967479675 |
| *Orthomyxoviridae* | 0.9714286 | 0.995571429 | 0.971428571 | 1 |
| *Papillomaviridae* | 0.8674699 | 0.949638554 | 0.933433735 | 0.951807229 |
| *Paramyxoviridae* | 0.9470199 | 0.962913907 | 0.933774834 | 0.973509934 |
| *Parvoviridae* | 0.95 | 0.9885 | 0.975 | 0.991666667 |
| *Picornaviridae* | 0.9757576 | 0.975757576 | 0.975757576 | 0.975757576 |
| *Polyomaviridae* | 0.826087 | 0.952898551 | 0.942028986 | 0.956521739 |
| *Potyviridae* | 0.9090909 | 0.977090909 | 0.945454545 | 0.981818182 |
| *Poxviridae* | 0.8392857 | 0.925 | 0.910714286 | 0.928571429 |
| *Reoviridae* | 0.9714286 | 0.968571429 | 0.957142857 | 0.971428571 |
| *Retroviridae* | 0.9613527 | 0.998067633 | 0.990338164 | 1 |
| *Rhabdoviridae* | 0.9894737 | 0.988526316 | 0.978947368 | 0.989473684 |
| *Togaviridae* | 0.9562044 | 0.96379562 | 0.948540146 | 0.97080292 |
